# Multi-Omic, Multi-Tissue Responses to Acute Exercise in Sedentary Adults: Findings from the Molecular Transducers of Physical Activity Consortium

**DOI:** 10.64898/2026.02.27.702183

**Published:** 2026-03-02

**Authors:** MoTrPAC Study Group, Daniel H. Katz, Christopher A. Jin, Gina M. Many, Gregory R. Smith, Hasmik Keshishian, Natalie M. Clark, Gayatri Iyer, Cheehoon Ahn, Malene E. Lindholm, Tyler J. Sagendorf, David Amar, Jacob L. Barber, Anna R. Brandt, Paul M. Coen, Yongchao Ge, Patrick Hart, Fang-Chi Hsu, Byron C. Jaeger, David Jimenez-Morales, Damon T. Leach, D. R. Mani, Samuel Montalvo, Hanna Pincas, Prashant Rao, James A. Sanford, Kevin S. Smith, Nikolai G. Vetr, Joshua N. Adkins, Euan A. Ashley, Steven A. Carr, Michael E. Miller, Stephen B. Montgomery, Venugopalan D. Nair, Jeremy M. Robbins, Michael P. Snyder, Lauren M. Sparks, Russell Tracy, Martin J. Walsh, Matthew T. Wheeler, Ashley Y. Xia, Stuart C. Sealfon, Robert E. Gerszten, Scott Trappe, Charles F. Burant, Bret H. Goodpaster

**Keywords:** exercise physiology, multi-omics, skeletal muscle, adipose tissue, biomarkers, endurance exercise, resistance exercise, physical activity, proteomics, metabolomics, transcriptomics, exerkines, systems biology, precision medicine, acute exercise response, molecular mechanisms, cross-tissue analysis, phosphoproteomics, epigenomics

## Abstract

Regular physical activity represents one of the greatest mechanisms for maintaining human health, yet the underlying molecular transducers of these benefits remain incompletely understood. Multi-omic assays now provide new opportunities to study the coordinated molecular responses of body tissues to different exercise modalities. The Molecular Transducers of Physical Activity Consortium (MoTrPAC) was established to address this need by creating a molecular map of the response to physical activity. Described here is the first human cohort of MoTrPAC: sedentary adults enrolled prior to study suspension during the COVID-19 pandemic (N=175) randomized to either endurance or resistance exercise, or non-exercise control. From these participants, we detail their global acute molecular response in skeletal muscle, adipose tissue, and blood, integrated at multiple levels: tissue, exercise modality, timepoint, and omic category. These analyses characterize key molecular pathways, identify central regulators, and implicate novel candidate exerkines in mediating multi-organ exercise effects.

## Introduction

Physical activity is known to improve human health and prevent illness, with benefits extending to multiple domains, including cognitive, metabolic, cardiovascular, musculoskeletal, gastrointestinal, and reproductive health.^1^ However, the mechanisms by which physical activity and exercise can prevent and alleviate many disease conditions need further delineation.^2^

For health benefits to accrue with regular exercise, each acute bout of exercise must initiate a cascade of molecular events that, when repeated over time, leads to the long-term effects of exercise training. However, the precise nature of the acute molecular response to exercise remains incompletely understood. Several molecular responses have been described, including flux in intracellular factors (e.g. ATP, NAD) in the contracting muscle and other tissues,^3,4^ provision of fuel for contracting muscle and protein accretion,^5^ and alterations in circulating signaling molecules (e.g. exerkines).^6^ Further, acute exercise effects are known to impact cellular behavior across molecular classes, including the methylome, the accessible chromatin landscape, the transcriptome, the proteome, the phosphoproteome, and the metabolome.^7^

While a number of studies have examined the epigenomic,^8–11^ transcriptomic,^12–17^ proteomic/phosphoproteomic,^18–22^ and/or metabolomic/lipidomic responses to acute exercise,^23,24^ the majority have focused on a single “ome” and a single tissue. Studies frequently examine skeletal muscle (SKM), with only a handful examining adipose tissue (AT)^25,26^ or blood.^27–31^ Thus, there is a critical need for an integrated multi-omic, cross-tissue, temporally resolved approach to elucidate the molecular responses to multiple exercise modalities. Such data can further identify novel therapeutic targets and inform more precise exercise recommendations for specific groups.^32^

The Molecular Transducers of Physical Activity Consortium (MoTrPAC), a National Institutes of Health Common Fund program, was created to build an extensive molecular “map” of exercise. MoTrPAC has previously released a multi-omic, multi-tissue dataset mapping the molecular response to endurance exercise training in rats.^33^ Here, we provide the first human data, derived from participants enrolled prior to study suspension during the COVID-19 pandemic. These sedentary adult volunteers were randomized to undergo an acute bout of either endurance or resistance exercise (EE, RE) or to remain supine or seated as non-exercise controls (CON). Each provided multiple blood (PAXgene and plasma), SKM (vastus lateralis), and AT (periumbilical) samples for multi-omic profiling at multiple timepoints. These multi-tissue, multi-omic, multi-modality, multi-timepoint data provide the basis for an integrative map of the acute response to resistance and endurance exercise. Herein we provide a global landscape of the multi-tissue response; a detailed accounting of the clinical intervention (ref Clinical Landscape, in preparation/submission) and companion works highlighting each individual tissue (ref Blood, Adipose, Muscle companions, in preparation/submission) as well as a detailed analysis of splicing variation (ref Splicing companion, in preparation/submission) are available separately.

## Results

### Study design and participant characteristics

**Figure 1 -:**
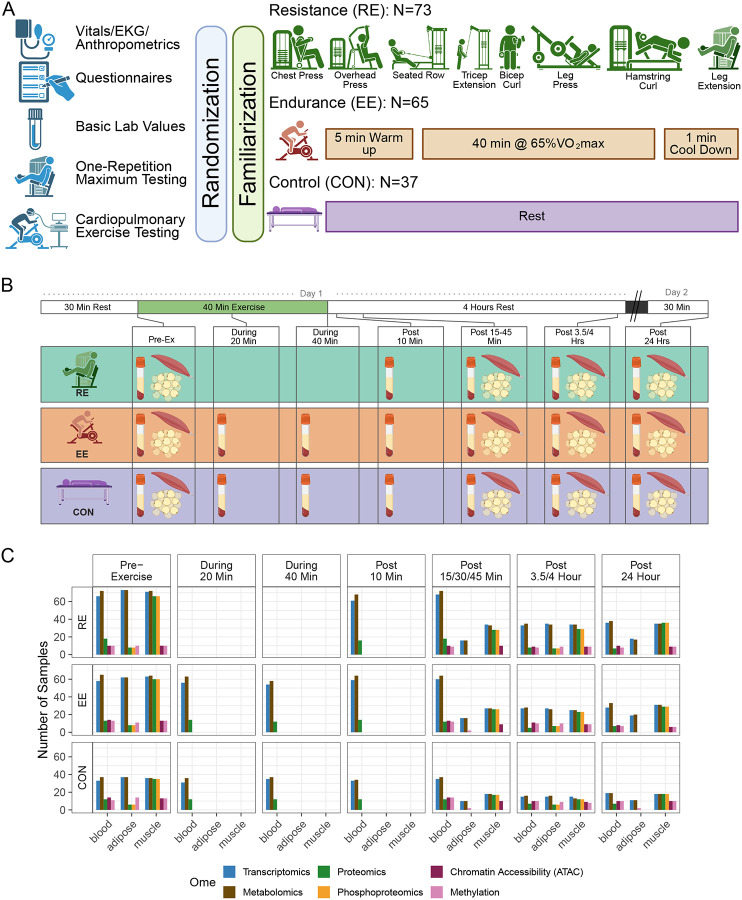
Study overview (A) Overview of the acute exercise bout. Baseline testing is completed before randomization. After randomization, a familiarization period for exercise is completed. Patients arrive in the morning, fasted, to complete their respective interventions. Created in https://BioRender.com. (B) Schematic of muscle, blood, and adipose biospecimen collection. No samples are collected during resistance exercise. Created in https://BioRender.com. (C) Number of samples measured in each omic category (on any assay) by tissue, exercise modality and timepoint. See Supplementary Figure S1 to see overlapping measurements for all participants. ATAC = Assay for Transposase-Accessible Chromatin

We analyzed 175 previously sedentary individuals who participated in an initial acute exercise intervention (or rest) and provided at least one tissue sample for analysis that resulted in data from at least one molecular assay.^34^ The trial components and acute exercise bout were standardized and performed across clinical centers with further details in a separate manuscript (Methods, Figure 1A and 1B, ref Clinical landscape, in preparation/submission). In brief, participants first underwent baseline testing including anthropometrics, vitals, clinical labs, cardiopulmonary exercise testing (CPET), and strength testing. Participants were randomized in an approximate 8:8:3 ratio to EE, RE, or CON groups and also to temporal profiles of biospecimen collection (Methods). A non-exercising group was deemed critical to control for the molecular effects of circadian rhythm, fasting, tissue sampling, and any other non-exercise intervention stimulus. The majority of participants were female (72%). Females represented a larger percentage of the CON group as compared to EE and RE. Mean age was 41 ± 15 years, the average BMI was 26.9 ± 4.0 kg/m^2^, average waist circumference was 92 ± 12 cm. Baseline CPET testing by cycle ergometer showed an average VO_2_peak of 24 ± 7.0 ml/kg/min (Females = 22.2 ± 5.12; Males = 31.3 ± 7.09). Participant surveys and whole genome sequencing derived ancestry analysis showed a diversity of racial, ethnic, and genetic backgrounds (Table 1, Figure S1A).

After randomization, sedentary participants assigned to EE or RE had 2 or 3 familiarization sessions that were completed 5 to 8 days before the acute exercise test. Participants arrived at the testing facility in the morning, following an overnight fast, then completed their required intervention. Biospecimens for multi-omic analysis were collected from three tissues (blood, SKM, and AT) at multiple timepoints before, during, and after exercise (see Methods). All participants had pre-exercise sampling from all three tissues (Figure 1B). Several factors influenced the number of samples collected and profiled at any given combination of group, tissue, timepoint, and assay, including participant burden and technical feasibility (Figures 1B–C, Figure S1B, Tables S1, Methods). As an example, proteome and phosphoproteome profiling was performed at all available timepoints on all available samples in SKM, while in AT, proteomic profiling was limited to just the middle timepoint. Thus combinations of tissue-ome-timepoint have varying power to detect differences in molecular abundance.

For each tissue-ome combination (i.e. SKM metabolomics), principal components of variation, using all feature measurements, were correlated to technical and phenotypic variables. While exercise group, timepoint, and covariates like age and sex accounted for a large proportion of the variation in molecular features, for most tissue-ome combinations, effects attributable to individual differences as captured by participant ID (PID) accounted a larger percentage of variation (Figures S1C,D).

### Exercise induced multi-omic changes

We utilized a difference-in-changes model to estimate the effects of exercise independent of control effects and identify differentially abundant (DA) molecular features. The model compares the change in abundance of any given feature from pre-exercise to each post-exercise timepoint in the exercise groups, relative to the corresponding change in the control group (see Methods). Comparison to the control group reduced false attribution of non-exercise effects (see below, Figures S2A–B).

**Figure 2 -:**
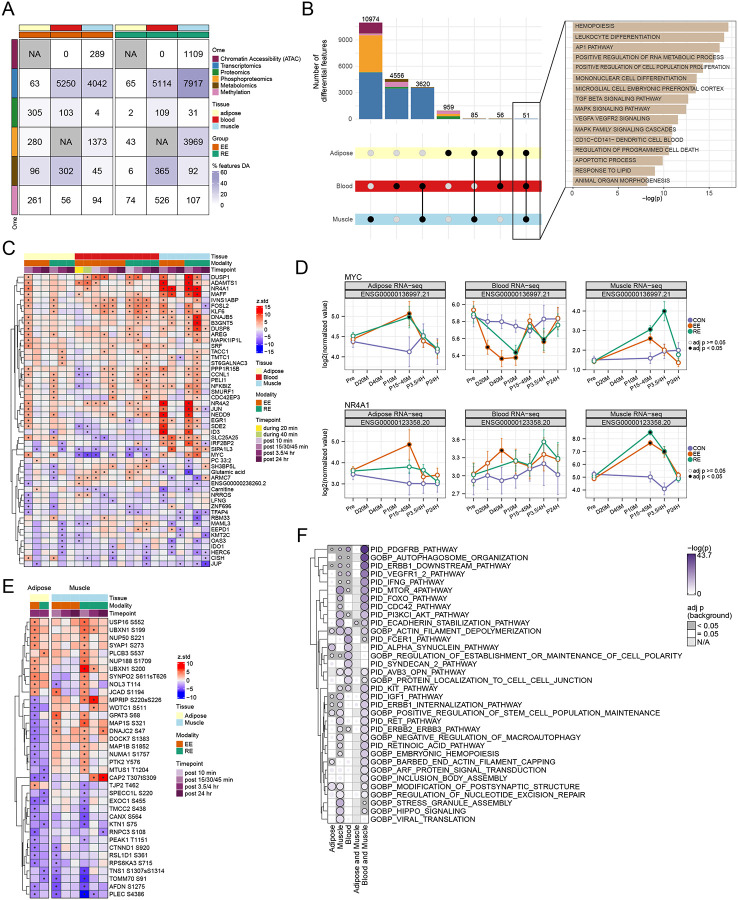
Exercise-induced multi-omic changes (A) The heatmap displays the number (annotated in each cell) of differentially abundant (DA) features (adj. p-value < 0.05) by ome, tissue, and exercise modality combination at any timepoint. The color intensity of each box reflects the percentage of DA features relative to total assayed features according to omic layer (see color bar), where gray indicates not analyzed. [Abbreviations: EE (Endurance Exercise); RE (Resistance Exercise)]. (B) UpSet plot of DA features across all tissues, exercise modalities and omes. Black dots/lines represent the intersection of DA omic features by tissue. Number of DA features of any ome are indicated on top of the bars. Over-representation analysis of 48 shared DA transcripts between all three tissues (right). (C) Heatmap of all 51 DA features (transcripts and metabolites) present in all three tissues presented according to timepoint and exercise modality. Z-scores of data are used for the heatmap. (*) indicates adj. p-value < 0.05. (D) Temporal trajectories of MYC and NR4A1 transcripts that are DA in all three tissues. EE = Endurance exercise, RE = resistance exercise, CON = control group, Pre = pre-exercise, D20M = during 20 minutes, D40M = during 40 minutes, P10M = 10 minutes post exercise, P15–45M = 15, 30, or 45 minutes post exercise (depending on tissue), P3.5–4H = 3.5 or 4 hours post exercise (depending on tissue), P24H = 24 hours post exercise. Data is shown as group-timepoint mean ± 95% confidence interval for the group mean with a black circle indicating model significance relative to control at adj. p-val<0.05. Plots indicate Ensembl ID. (E) Heatmap of 36 DA phosphosites shared between the SKM and AT. Phosphoproteomics was only performed at the 3.5 hr timepoint in the AT. Z-scores of data are used for the heatmap. (*) indicates adj. p-value < 0.05. (F) Heatmap showing ORA of DA features in each tissue and for features which overlap between two tissue pairs (SKM & blood and SKM & AT) using Molecular Signatures Database (MSigDB) C2 and C5 databases. Pathways shown are significant in at least one of the groups (adj. p-val < 0.05, as indicated by the grey background of each cell).

Exercise impacted thousands of molecular features, with significant variation between exercise modality, tissue, and ome (Figure 2A, Table S2). The majority of DA features were observed in only one tissue considering either exercise modality (Figure 2B). A total of 3,620 features were DA in both blood and SKM: driven by shared transcripts, while SKM and AT shared just 85 unique features. Overlap in DA features depended on ome. For example, for transcriptomics and metabolomics, agreement in SKM and blood was higher than SKM and AT. Of the 8929 DA transcripts in SKM, 40% were also DA in blood while just 1% were DA in AT. Of 106 DA metabolites in SKM, 68% were also DA in blood, but just 11% in AT. Phosphoproteomic agreement between SKM and AT was also quite low, less than 1% of 4231 DA phosphosites in SKM were DA in AT (Table S2). Differentially accessible peaks could only be detected in SKM (n = 1230 via ATAC-seq), while the greatest number of differentially methylated regions (n = 580) were observed in blood (Figure S2C). Across tissues, there was a single methylated region observed in both SKM and AT (Figure S2C). This peak was annotated to a long non-coding RNA (lncRNA) gene (ENSG00000245482) that is an antisense RNA to *ALG10* (Alpha-1,2-Glucosyltransferase), a protein involved in N-linked glycosylation.^35^

Despite largely tissue-specific DA feature responses, 51 DA features were shared across all three tissues–with 48 being transcripts (Figure 2B). Over-representation analysis (ORA) was used to assess their biological relevance and revealed pathway enrichments related to hemopoiesis, RNA metabolic processes, TGFβ, MAPK and VEGFR signaling, apoptosis, and animal organ morphogenesis (Figure 2B; Table S2). Among shared DA features, the direction of exercise effect varied across tissues and modalities (Figure 2C). For example, the proto-oncogene, *MYC*, which promotes VEGF-mediated angiogenesis,^36^ was decreased in the blood but increased in SKM and AT (Figures 2C–2D), whereas expression of the epithelial cell activator, *SIPA1L3*, decreased in blood and AT, but increased in SKM (Figures 2C–2D and S2D). Transcripts that shared directionality across tissues included the nuclear receptors *NR4A1* (aka *NUR77*) and *NR4A2*, the scaffold protein *NEDD9* (Enhancer of filamentation 1), and the NFkB inhibitor, *NFKBIZ*, which was acutely downregulated during EE in the blood, but then displayed elevated expression following both EE and RE in all tissues (Figures 2C–2D and S2D).

Among 36 shared DA phosphosites between SKM and AT, 24 shared directionality across tissues (14 up, 10 down) (Figure 2E). For example, pS920 of CTNND1, a protein involved in c-MYC and CDK1 signaling,^37^ decreased with EE in AT and SKM, suggesting a multi-tissue role for this phosphosite in acute exercise. The pS68 of GPAT3, an enzyme involved in lipid synthesis,^38,39^ decreased with EE in AT and increased with both exercise modalities in SKM, supportive of altered substrate flux between AT and SKM in response to the energetic demands of acute exercise.

To further investigate phosphospecific signatures that were distinct or shared between SKM and AT, we performed over-representation analysis (ORA) on DA phosphosites using PTMSigDB, which is a site-level pathway database designed for use with phosphoproteomics data (Figure S2E, Table S2).^40^ The SKM DA phosphosites were annotated to 87 enriched pathways that included kinase pathways such as MAPKs, MAPKAPKs, ERK1/2, MTOR, CDK1/2/5, and GSK3A/B. AT and SKM shared 3 enriched pathway signatures that included perturbations related to BYL719, a PI3K inhibitor, and RO4929097, a gamma secretase inhibitor that targets NOTCH signaling^41^, and MAP2K2 signaling.

Metabolomic effects were most likely to be detected in blood, with substantial overlap in DA metabolites between blood and SKM (n=69) and blood and AT (n=48) (Figure S2C). All three tissues shared three DA metabolites: carnitine, glutamic acid, and PC 33:2 (Figure 2C).

Given the paucity of shared individual features across tissues, we investigated shared biology at the pathway level via ORA of DA features. Analysis was done using DA features observed in each individual tissue as well as on features which overlapped in blood/SKM and AT/SKM (i.e. tissue pairs where overlap was sufficient for analysis). Pathway enrichment analysis revealed enrichment of PDGFRB, actin filament depolarization, and regulation of establishment of cell polarity across all three tissues, and MTOR, retinoic acid, KIT, VEGFR, ERBB1 and IFN transcriptomic signaling pathways among those shared between the SKM and blood (Figure 2F, Table S2). The observation of shared pathways contrasted with relatively little overlap between actual DA molecules across tissues and timepoints (Figures 2B and S2C), suggesting conserved pathway level responses to exercise, but tissue-specific molecular regulation.

Within tissues, gene-by-gene agreement was assessed across omes where data was available. While most DA features were DA in only one omic-layer, there were notable numbers of genes that were DA in 2 or more. In particular, 826 genes in SKM were DA by transcriptomics and by phosphoproteomics. In blood, 63 genes had changes in transcriptional levels and plasma proteomic levels (Figure S2F). These data are explored more in tissue specific companions (ref muscle and adipose companions, in preparation/submission) and in later sections of this paper.

Finally, the effect of the control group on results was also explored in greater detail. Specifically, the main difference-in-changes model was compared to the simple effect of timepoint in each group (EE/RE/CON). Due to the presence of non-exercising controls, molecules which changed in concentration during exercise (such as *PPARGC1A* in exercising SKM, Figure S2B) could be differentiated from those that did not actually change per se, but were nonetheless affected by exercise. As an example, the transcript *PER2* was unchanged in SKM-RE relative to baseline, but was significantly higher compared to control (Figure S2B) at 3.5 hours post exercise, indicating an alteration of normal circadian signaling in RE.

### Temporal molecular responses with acute exercise

**Figure 3 -:**
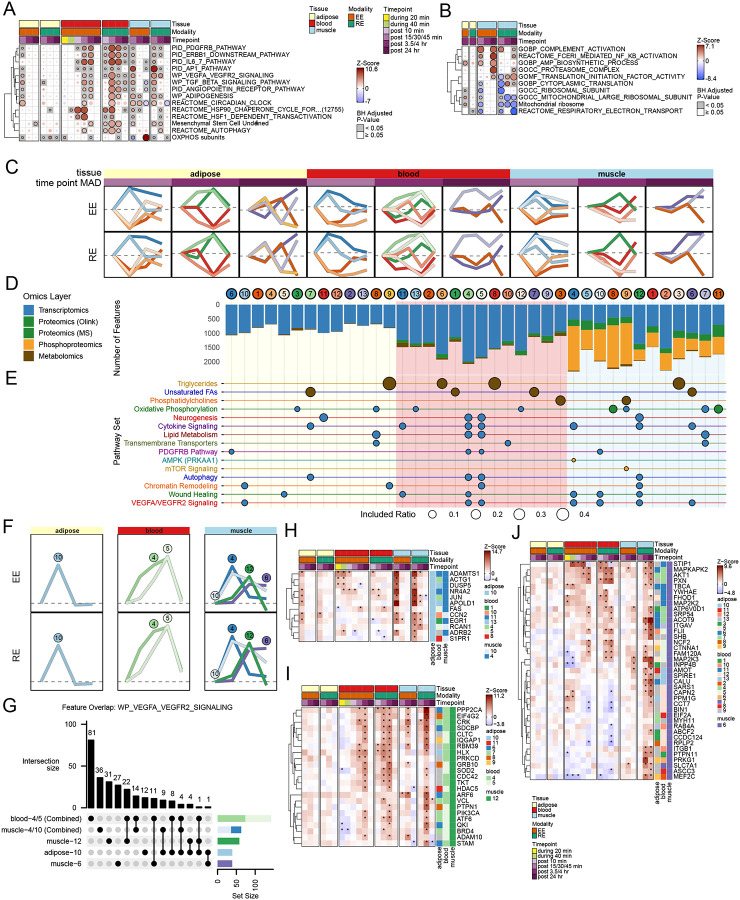
Temporal mapping of the molecular response to exercise across tissues A-B) Pathway Enrichment for each tissue-timepoint in transcriptome and proteome utilizing pathway enrichment via Pre-ranked Correlation-Adjusted MEan-RAnk (CAMERA-PR). (C) Temporal trajectories (sparklines) of all cluster centroids identified by fuzzy c-means clustering. Each line represents the average Z-score profile of a single cluster. Line color indicates the cluster, to be matched to the colored circles below. Clusters are organized by tissue and then by timepoint of maximal response (maximal absolute deviation [MAD] from zero); light purple indicates Early (15–45 minutes [and/or 10 minutes for blood]), medium purple indicates Mid (3.5–4 hours), and dark purple indicates Late (24 hours). Plots are split top and bottom by exercise modality (Endurance, EE; Resistance, RE). (D) Omics composition of hard-clustered features. Each column represents a distinct cluster, ordered by tissue and temporal profile. The bar chart shows the total number of features assigned to each cluster (with a membership probability > 0.3), with bar segments colored by their omics layer of origin. Circles at the top display the cluster ID, with fill color corresponding to the temporal trajectory profile shown in (C). Background shading indicates the tissue of origin (adipose, blood, or muscle). (E) Functional enrichment of clusters by tissue-ome. The dot plot displays significant pathway enrichments (FDR < 0.05) for each cluster shown in (D). The size of each point is proportional to the “included ratio” (the proportion of features within the cluster that belong to the pathway set). The fill color of each point indicates the omics layer of the enriched features. Pathway abbreviations: Cytokine Signaling = REACTOME_CYTOKINE_SIGNALING_IN_IMMUNE_SYSTEM, VEGFA/VEGFR2 Signaling = WP_VEGFA_VEGFR2_SIGNALING, Wound Healing = GOBP_WOUND_HEALING, PDGFRB Pathway = PID_PDGFRB_PATHWAY, Chromatin Remodeling = GOBP_CHROMATIN_REMODELING, Neurogenesis = GOBP_NEUROGENESIS, Lipid Metabolism = GOBP_LIPID_METABOLIC_PROCESS, Unsaturated FAs = REFMET_Unsaturated FA, Triglycerides = REFMET_TG, Phosphatidylcholines = REFMET_PC, Transmembrane Transporters = GOMF_ACTIVE_TRANSMEMBRANE_TRANSPORTER_ACTIVITY, Oxidative Phosphorylation = MITOCARTA_OXPHOS, Autophagy = GOBP_PROCESS_UTILIZING_AUTOPHAGIC_MECHANISM, mTOR Signaling = PSP_MTOR, AMPK (PRKAA1) = PSP_PRKAA1 (F) Sparklines for c-means cluster trajectories which are enriched for VEGF pathway features. These are identical to the trajectories in C and are reprinted for clarity. (G) UpSet plot comparing feature membership in each cluster shown in (G). Clusters with matching timepoint of maximal absolute deviation are paired for brevity. (H-J) Multi-tissue heatmaps of Z-scores for transcriptomic features belonging to the VEGFA-VEGFR2 signaling pathway. Columns represent timepoints grouped by tissue and exercise modality. The right-hand annotation indicates the c-means cluster assignment for each gene in adipose, blood, and muscle, with colors corresponding to the cluster profiles in (C,D, and F). (H) Features assigned to adipose cluster 10 and muscle clusters 4 or 10. (I) Features assigned to blood cluster 4 or 5 and muscle cluster 10. (J) Features assigned to muscle cluster 6.

To explore how molecular responses to acute exercise evolve over time, we next examined pathway enrichments in each tissue at each timepoint, separately. While hundreds of pathways were affected across tissues and timepoints, we focused on shared pathway enrichments across all three tissues for the transcriptome (Figure 3A), proteome (Figure 3B) and metabolome (Figure S3A). Consistent with DA feature ORA analysis (Figure 2B), key pathways including VEGF signaling, adipogenesis, AP1 signaling, EGFR signaling (e.g. ERBBA), oxidative phosphorylation, and circadian clock genes emerged as top enriched pathways across all three tissues at the transcriptomic level.

In SKM at the proteomic level, both modalities resulted in proteasome complex enrichment at 24 hours post exercise. Interestingly, following RE, the late increase in proteasome complex proteins co-occurred with a significant decrease in oxidative phosphorylation (OXPHOS) proteins. When contrasting RE and EE, the proteomic vs. transcriptomic effects of exercise on OXPHOS features appears to be inverse: in EE, late transcription of OXPHOS genes is strongly induced, while proteomic levels remain stable. However, in RE, enrichment for OXPHOS transcripts is less robust, and OXPHOS protein levels decline significantly (Figures 3A and 3B). This suggests increased gene transcription may offset proteasome-based clearance of OXPHOS proteins in EE, but not in RE. As a central fuel source for OXPHOS, triglycerides decreased across all tissues in response to EE and RE, while unsaturated fatty acids decreased during and early post exercise in blood, to then rebound and increase slightly at later post exercise timepoints across all tissues, primarily in response to RE (Figure S3A).

To better understand multi-omic trajectories, we analyzed temporal molecular signatures across the post-exercise timepoints using fuzzy c-means clustering, considering EE and RE *simultaneously*, meaning that any given cluster represents a combined EE and RE temporal signature. To balance dimension reduction with specificity, we identified 13 clusters in AT and blood and 12 in SKM (Figure 3C, see Methods). The resulting c-means clusters showed highly consistent patterns between EE and RE; the algorithm did not identify a prominent cluster of features which showed modality specific but markedly different behaviors between EE and RE.

Omic contributions to each cluster varied widely (Figure 3D); in SKM, phosphoproteomic changes were more prevalent in trajectories with early maximal responses (muscle clusters 4, 5, 10, 8, and 9). Transcriptomic features were more likely to show a maximal response at 3.5 hours (muscle clusters 12, 1, 2, and 3). Proteomic changes were relatively evenly distributed. In blood, metabolic features appeared more prevalent in trajectories with early maximal responses, though trajectories with late maximal responses also frequently contained metabolites.

When ORA was applied to features assigned to each cluster, a more nuanced picture of common pathway enrichments emerged (Figure 3E, Table S3). For example, in SKM, cytokine signaling transcripts were enriched in clusters with early, middle, and late peaking trajectories. Alternatively, autophagy transcriptomic features were significantly enriched in temporal clusters with mid-peaking signatures across all three tissues. Triglycerides, as a fuel source, were enriched in temporal trajectories that were downtrending, but each tissue favored different time signatures; triglycerides in SKM and AT favored patterns with later depletion, while blood triglycerides were enriched within clusters with early to mid response patterns. Taken together, these results demonstrate that known pathways and molecular feature sets can display multiple temporal patterns in response to an exercise stimulus.

The VEGF signaling pathway, critical to many of the regenerative properties of exercise,^42–44^ provides an illustrative example of this temporal diversity within a single pathway. Seven temporal clusters were enriched with VEGF signalling features, 4 within SKM (Figure 3E). In SKM, these clusters demonstrated maximal deviations from 0 spanning the temporal spectrum (Figure 3C, 3D, 3F). Notably, 3 of the SKM clusters (10, 12, and 6) were elevated at the middle timepoint (3.5 hours in SKM, Figure 3F) but each followed different trajectories, highlighting the importance of tracing these trajectories, rather than simply analyzing each timepoint. Within each tissue, cluster membership is non-overlapping by definition, but between tissues we observed very modest overlap (Figure 3G), consistent with global analyses (Figure 2B). Nonetheless, there were some overlapping features. For example, shared between adipose-10 and muscle-4/10 were transcription factors (TFs) including *NR4A2*, *EGR1*, and *JUN*, as well as key angiogenic regulators like *APOLD1* and *ADAMTS1* (Figure 3H). Importantly, ADAMTS1 plays an anti-angiogenic role,^45^ highlighting that molecular brakes are stimulated by exercise temporally along with molecular activators. In AT, *CCN2* displayed the most significant modality-consistent upregulation at 45 minutes post-exercise; the pattern in SKM was similar, although *CCN2* activity persisted to 3.5 hours with RE. Also known as connective tissue growth factor (CTGF), *CCN2* expression is induced in response to VEGF in endothelial cells.^46^

There was also overlap between features in blood and SKM whose trajectories were highest at 3.5 hours post exercise (Figure 3I). These transcripts include key regulators for a variety of adaptive processes relevant to both tissues. For example, growth and differentiation factors like *CRK* and *PI3KCA* are upregulated along with factors to balance growth signals, such as *GRB10* and *PTPN1*.^47–50^. This group also contains a handful of novel findings. *SDCBP* which encodes syntenin-1 plays a role in exosome formation,^51^ positioning it as a central regulator for general exerkine signaling. *HLX* is a hematopoietic TF linked to leukemia; in the setting of exercise it may be resetting blood and SKM for differentiation and regeneration.^52^

Our analysis also identified a trajectory enriched for VEGF pathway transcripts which persist at their highest levels in SKM to 24 hours post exercise (cluster 6, Figure 3F). These features are candidates for setting in motion long term adaptation, once the initial stress of the acute bout has passed. Of note, muscle-6 features have some increased transcription at 3.5 hours as well (Figure 3J). These features appear to have no clear role in AT, but in blood seem to roughly split into an up and down regulated group. For example, *INPP4B* is upregulated in SKM, but downregulated in blood during exercise (EE only). This fits with the known function of *INPP4B* as both tumor suppressor and oncogene, depending on context.^53^ *INPP4B* was previously shown in a small study to induce with exercise in SKM, but here we show the opposing behavior in blood.^13^ As a suppressor of the PI3K-Akt pathway, it may act as counter-regulatory in SKM, but activating in blood.^54^ Angiomotin, encoded by the *AMOT* gene, is a key factor in angiogenic patterning,^55^ though is not previously described in human exercise adaptation; in rats, exercise is known to affect isoform balance.^56^ Finally, *ASCC3* may be one of the more important novel findings as it is involved in DNA repair, and thus may be a key component of exercise-induced DNA defense. The role may be particularly important in SKM, as a rare form of neuromuscular disease has been described in persons with biallelic variants in *ASCC3*.^57^

Taken together, these analyses not only reveal novel exercise response molecules, but also a nuanced and often tissue-specific temporal regulation of key exercise response pathways. These data can be explored across a range of other enriched pathways, such as autophagy (Figures S3D–F), where blood and SKM exhibited temporally similar pathway induction, but with largely divergent transcriptional programs.

### Coordinated molecular behaviors across tissues

**Figure 4 -:**
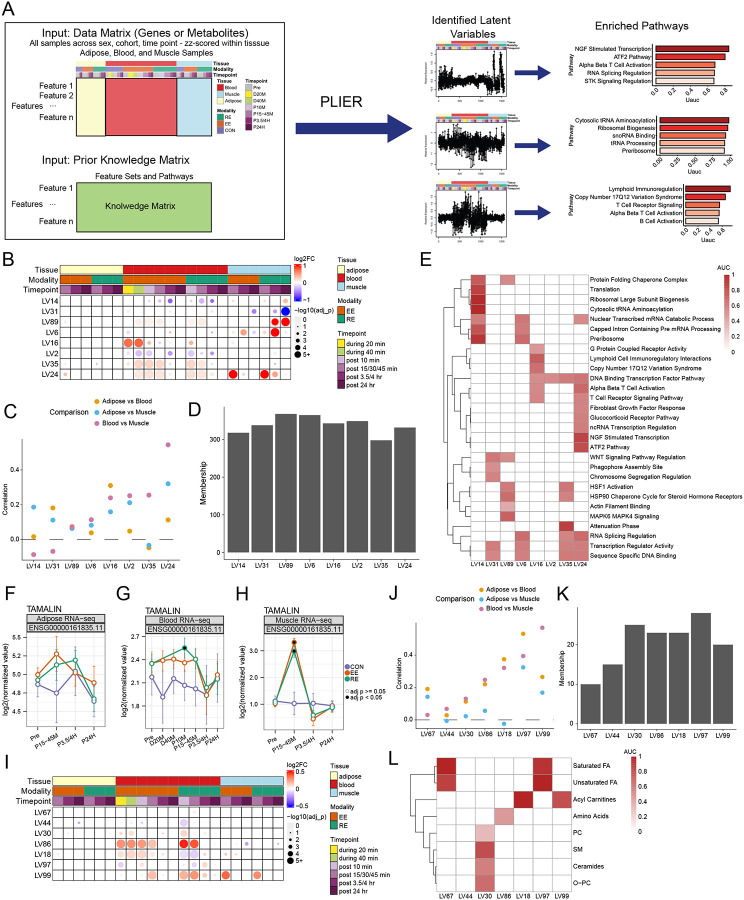
Coordinated tissue responses (A) Schematic of PLIER method design as applied. PLIER used as input Z-scored normalized RNAseq data from all three tissues concatenated into a single input matrix where rows were the overlapping feature set and columns were the samples in each of the three tissues, as well as a matrix of prior knowledge for the inputted feature set. PLIER outputs a series of Latent Variables (LVs) reflecting distinct patterns of behavior across the measured samples with a set of associated features that align with the pattern and specific pathway enrichments based upon the prior knowledge and associated feature set. PLIER was run separately on the cross-tissue RNAseq and cross-tissue metabolomics data. (B) Bubble heatmap of the significance (size = adjusted p-value) and directionality (color = log2 fold-change) of the EE or RE response in the transcriptomic data in each tissue described by a subset of LVs with the most significant identified transcriptomic exercise responses. (C,D) The cross-tissue Pearson correlation (C) and count of highly associated genes (D) for each of the RNAseq LVs described in (B). PLIER outputs a membership score for each feature to each LV. Membership is determined by a z-scored membership score > 3 for a given feature relative to all features tested. (E) Top enriched pathways for the RNAseq LVs described in (B). Scale is the AUC of a statistical test of association between pathway and LV associated genes. The top five significant pathways are included for each LV, if they exist. (F,G,H) Feature plots of gene TAMALIN response to acute exercise in adipose (F), blood (G), and muscle (H). (I) Bubble heatmap of the significance (size = adjusted p-value) and directionality (color = log2 fold-change) of the EE or RE response in the metabolomic data in each tissue described by a subset of LVs with the most significant identified metabolomic exercise responses. (J,K) The cross-tissue Pearson correlation (J) and count of highly associated metabolites (K) for each of the metabolomics LVs described in (I). (L) Significantly enriched metabolite classifications for the metabolomics LVs described in (I). Scale is the AUC of a statistical test of association between classification and LV-associated genes.

To capture coordinated multi-tissue responses, Pathway Level Information ExtractoR (PLIER)^58^ was applied. PLIER is an analytical approach that identifies groups of genes or metabolites that respond consistently in blood, SKM, and AT (Figure 4A). This method differs from the unsupervised, tissue-by-tissue c-means analysis by examining all three tissues simultaneously and incorporating known biological pathway information. Feature groups in PLIER often show similar multi-tissue behaviors, though the trajectory the features take in each tissue may differ.

We considered transcripts and metabolites separately and first defined transcript response patterns (called latent variables, or LVs), and highlighted 8 which showed significant exercise-induced changes across tissues (Figures 4B and Figure S4A–4H; Table S4). Each pattern showed varying cross-tissue correlation, summarized approximately 300 genes (Figure 4C and 4D), and was associated with at least one known biological pathway (Figure 4E).

At least one group (LV24) revealed genes that increased in coordinated fashion across all three tissues following both exercise types (Figure 4B–C, S4E). This group generally highlighted important TFs, including *NR4A1 and NR4A2,* which are well-known regulators of SKM adaptation,^59^ and are identified in our analyses above (Figures 2C–2D and 3H). While their role in SKM is well known,^59^ they have also been implicated in mediating inflammatory responses in macrophages, suggesting a role for macrophage mobilization and activation.^60,61^ The nerve growth factor (NGF) stimulated transcription pathway was also enriched in this LV (Figure 4E). NGF acts as a bridge between the nervous and immune systems: it is involved in nerve cell growth and also rapidly generated at inflammation sites. Both NGF and NR4A1 regulate NF-κβ, a master regulator of inflammation, consistent with exercise's known ability to trigger controlled inflammatory responses.^62,63^ Many NGF member genes are known to be connected to exercise, but *TAMALIN* is a novel exercise responsive gene in LV24 (Figures 4F–4H and S4E). While *TAMALIN* is known to be a neuronal scaffold protein, linking receptors to neuronal proteins, it is not part of the established NGF pathway. The observed co-variation may be unmasked uniquely by exercise, and warrants further exploration.

Several other biological pathways are highlighted by each LV. One LV was strongly correlated with ribosomal biogenesis (LV14), a process that has been linked to SKM hypertrophy^64^, and displayed contrasting patterns of expression in SKM vs. blood, with an increased expression in SKM at the late timepoint (24 h) after RE, and a decreased expression in blood at the intermediate timepoint (3.5 h) in both exercise modalities (Figure S4C).

Three response patterns (LV6, LV24, LV35) highlighted alterations in splicing machinery in multiple tissues (Figure 4E, S4B, S4E, S4G, Ref. MoTrPAC muscle and splicing papers [in preparation/submission]). Key splicing factor genes, including *HNRNPU* and *DYRK1A* (LV6), increased in blood and SKM at 3.5 hours in both EE and RE.

MAPK signaling and heat shock-associated LV89 (Figure S4H) reflected an RE-specific increase in expression at 3.5 and 24 hour timepoints in SKM. RE-specific MAPK signaling has been linked to significant EE vs RE response discrepancies in SKM atrophy-associated genes (Ref. MoTrPAC muscle paper [in preparation/submission]).

In the metabolomic analysis, we highlight 7 LVs (Figures 4I–4J and S4I–O; Table S4). Between 10 and 30 metabolites were associated with each highlighted LV (Figure 4K). Six of the 7 LVs were enriched for at least one known metabolite classification (Figure 4L).

The metabolic patterns revealed fundamental differences in how the body mobilizes energy during different exercise types. Represented by LV18, long-chain acylcarnitines—byproducts of fatty acid oxidation—increased after EE but decreased after RE in both blood and SKM (Figure S4I).^65^ Consistent with this observation, we identify EE-specific upregulation of key fat-burning genes *HADHA*, *MLYCD*, and *ACAA2* in a SKM-focused companion analysis, emphasizing this key metabolic fuel switch. (Ref muscle companion in preparation/submission).

Two other LVs emerged emphasizing blood-SKM interaction. LV86 included a range of metabolic factors such as amino acids, lactate, pyruvate, inosine, and hypoxanthine (Figure S4M). This group collectively increased in blood during and after both exercise interventions, but in SKM, values decreased slightly at 24 hours. Many of these metabolites are byproducts of energy production (TCA intermediates, glycolysis products) showing up in the plasma.^66^ The small dip in SKM at 24 hours may point to an overcompensation in clearance. LV99 represented an alternative blood-SKM interaction. This metabolite set included citrate, isocitrate, malate, 3-hydroxybutyrate and short chain acylcarnitines (Figure S4O). In contrast to LV86, these features peaked in blood *and* SKM early after both exercise modalities, suggesting weaker clearance from SKM as compared to LV86. As a whole, these groups of features may represent a useful marker of exercise demand and adaptation.

### Exercise modality-specific effects across tissues and omes

**Figure 5 -:**
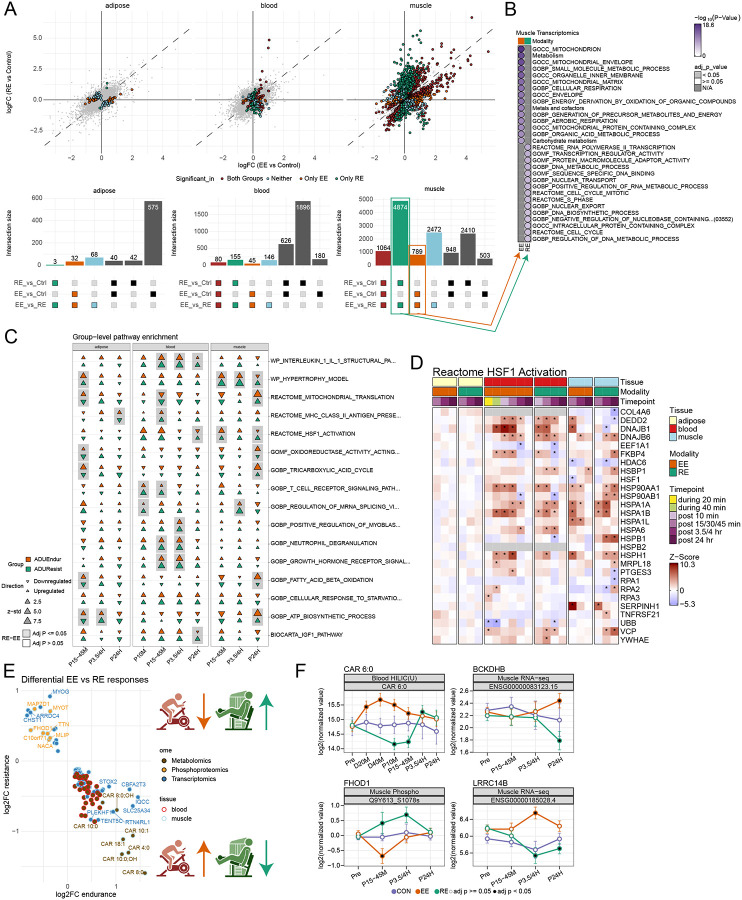
Differential responses to endurance and resistance exercise (A) Top panel: For all measured features, the log fold change (logFC) for EE vs control (x-axis) and RE vs control (y-axis) are plotted by points. Molecules which are significant (adj p-value <0.05) in the EE vs. RE comparison are indicated by colored points, and colors indicate the status of significance in EE vs control and RE vs control comparisons. Features which are non-significant in the EE vs RE comparison are indicated in light grey. For each feature, only the timepoint with the most significant difference between EE and RE is included to improve visualization. Bottom panel: UpSet plots display the intersection of features included in the top panel significant in EE vs. RE, EE vs. CON, and RE vs. CON comparisons. Colored intersection labels match the colors described in the top panel. (B) Overrepresentation analysis of muscle transcriptomic features from panel A specific to each exercise modality (green and orange points) using C2 and C5 MSigDB database. No enrichments were shared between the endurance-specific (orange) features and resistance-specific (green) features. Top 15 terms for each by p-value are shown. (C) CAMERA-PR (Correlation Adjusted MEan RAnk gene set test—PreRanked) pathway enrichment heatmaps highlighting pathways that are different between EE and RE. The CAMERA-PR results for EE vs Control and RE vs Control are described by orange and green triangles, respectively. The directionality of the triangle indicates upregulation in EE or RE relative to control. Any pathways that are significant when directly compared between EE and RE are highlighted with a light grey background. The top 5 pathways by lowest p-value in each tissue are visualized. (D) Multi-tissue heatmap of Z-scores for transcriptomic features belonging to the HSF1 pathway. Columns represent timepoints grouped by tissue and exercise modality. (E) Oppositely regulated features in the two exercise modalities (relative to control, adj. p-val<0.05). Inclusion requires features are also significant in a direct comparison between EE and RE (adj. p-val<0.05). Shown are blood and SKM (no features met these criteria in AT). (F) Timewise abundance changes of 4 specific features included in D. Pre = pre-exercise, D20M = during 20 minutes, D40M = during 40 minutes, P10M = 10 minutes post exercise, P15–45M = 15, 30, 45 minutes post exercise (depending on tissue), P3.5–4H = 3.5 or 4 hours post exercise (depending on tissue), P24H = 24 hours post exercise. Data is shown as group-timepoint mean ± 95% confidence interval with a black circle indicating significance relative to control at adj. p-val<0.05. CAR 6:0 = C6 Carnitine, BCKDHB = Branched Chain Keto Acid Dehydrogenase E1 Subunit Beta, FHOD1 = Formin Homology 2 Domain Containing 1, LRRC14B = Leucine Rich Repeat Containing 14B. Plots indicate Ensembl for RNA or Uniprot IDs for proteins where appropriate. HILIC(U) = Hydrophilic Interaction Liquid Chromatography (Untargeted) metabolomics platform.

Given findings of modality-specific differences in DA features and pathways, we sought next to explore these differences in depth. When compared to controls, a larger number of DA features were detected in RE for blood and SKM, whereas a larger number were detected in EE for AT (Figure 2A and S5A). A sizable proportion of DA features overlapped in the SKM (~22%) and blood (~23%), with very few features demonstrating DA effects in both modalities in AT (~2%, Figure S5A). To better understand modality differences, we modeled exercise-induced changes between EE and RE using a direct comparison at each timepoint. Thus, for any given molecular feature, statistically significant differences can be described for EE vs Control, RE vs Control, and EE vs RE. Figure 5A shows the relationship between EE vs Control effects and RE vs Control effects and highlights features which are statistically different in the EE vs RE comparison. Overall blood and AT exhibited few features that differed statistically between the modalities. In contrast, the majority of DA features in SKM were different between the two modalities: 4874 features were “RE-specific” meaning they were DA in RE, but not in EE, when compared to control. The number of “EE-specific” features was far smaller, just 789 (Figure 5A). ORA of these modality-specific subgroups demonstrated enrichments for mitochondrial, carbohydrate, and fat metabolism pathways in EE, vs enrichments for heat stress responses, cell cycle, and DNA biosynthesis in RE (Figure 5B).

To further illustrate pathway-level differences between EE and RE, we applied pathway enrichment via Pre-ranked Correlation-Adjusted MEan-RAnk (CAMERA-PR), which accounts for intergene correlations, to all EE vs RE model comparisons (Figure 5C). At the transcript level in SKM, RE showed stronger enrichment in terms related to muscle remodeling (e.g. hypertrophy, HSF1 activation, and RNA splicing). The HSF1 pattern highlights a temporal offset in activity between EE and RE, with RE upregulation persisting to 24 hours (Figure 5C). At the feature level, this is exemplified by *HSPH1* which is strongly induced by EE at 30 minutes post exercise in SKM, but not until 3.5 hours in RE (Figure 5D and S5B). The pattern of HSF1 modality differences was similar in blood at 24 hrs (Figure 5C), suggesting greater systemic stress-induced response following RE, though these differences are less evident at the feature level (Figure 5D).^67^ EE displayed enhanced mitochondrial enrichments to include fatty acid beta oxidation and ATP biosynthetic processes in the SKM. In the AT, EE-specific increases in enrichment for mitochondria-related pathways were observed earlier than in the SKM, at the 30 min post-exercise timepoint. In the blood, RE induced a more pronounced immune response indicated by enrichment in terms related to neutrophil degranulation and T-cell receptor signaling. RE enrichment was also present for the glycoprotein VI mediated activation cascade, a response to blood vessel damage (Table S5).

We next analyzed divergent EE and RE effects where features had opposing directions (e.g. upregulated relative to control in EE and downregulated relative to control in RE, adj. p-val <0.05). Based on direct contrast, 111 features were oppositely affected (38 in blood, 73 in SKM, and 0 in AT) (Figure 5E; Table S5). In blood, only metabolites were oppositely regulated; all increased in EE and decreased in RE (see e.g. CAR 6:0, Figure 5F), while SKM displayed divergent features across transcriptome, metabolome and phosphoproteome (Figure S5C). In blood and SKM, 3 acylcarnitines (CAR 10:1, CAR 11:0, CAR 18:1) increased in response to EE but decreased in response to RE at the same early post exercise timepoint, indicating accelerated fatty acid turnover dynamics in response to EE but not RE, consistent with the PLIER analysis.^68,69^

Other divergent responses reflect the dynamics of exercise metabolism in EE and RE. For example, expression of the *HIF1A* transcriptional corepressor *CBFA2T3* (Figure S5D) was increased in EE and decreased in RE, and several key HIF1A glycolytic response genes (e.g. *LDHA, PDK4* and *PFKP (data not shown)*) were upregulated in response to RE, suggesting modality-divergent mechanisms for fuel handling. Amino acid metabolism was also oppositely regulated in SKM in response to EE and RE, with expression of *BCKDHB*, a subunit of the rate-limiting branched chain amino acid metabolism BCKDH complex, increasing in response to EE and decreasing in response to RE (Figure 5F).

Divergent SKM transcriptional and phosphoproteomic level feature responses to EE and RE included known transcriptional regulators of SKM regeneration such as Myogenin (*MYOG)* (Figure S5D) and Atrogin-1 (*FBXO32*, ref Muscle companion paper, in preparation/submission). Here, the atrogene *FBXO32* (Atrogin-1), a component of the E3 ubiquitin ligase, was upregulated with EE and downregulated with RE; following a similar modality pattern was *ZBTB4*, a transcriptional repressor that has not been previously associated with exercise (Figure S5D).

Divergent phosphoproteomic signatures included pT114 of the anti-apoptotic factor NOL3 (Figure S5D), which increased in RE, with implications for muscle injury prevention.^70^ RE also increased phosphorylation of two proteins involved in structural integrity of the Z-disc: FHOD1 (Formin Homology Domain Protein 1, Figure 5F) and Myotilin (Figure S5D), which were decreased in EE and indicative of sarcomeric protection mechanisms following RE. The same pattern was observed for a novel lncRNA intronic to the cytoskeleton gene *LIMA1* (Figure S5D). Finally, *LRRC14B* – a gene with minimal experimental data, but which was recently identified as a regulator of striated muscle function and genetically associated with cardiac dysfunction –^71,72^ increased in EE and decreased in RE (Figure 5F). Taken together, these results highlight molecules that define the divergent phenotypic responses to EE and RE, and are thus critical targets for future investigation.

### Transcription factor response in acute exercise

In view of the substantial transcriptional response to exercise identified in this study, we sought to identify TF candidates underlying the transcriptomic responses to EE and RE in each tissue. Hence, we combined TF binding motif enrichment analysis of exercise-responsive genes with an assessment of the direct TF response to acute exercise.

**Figure 6 -:**
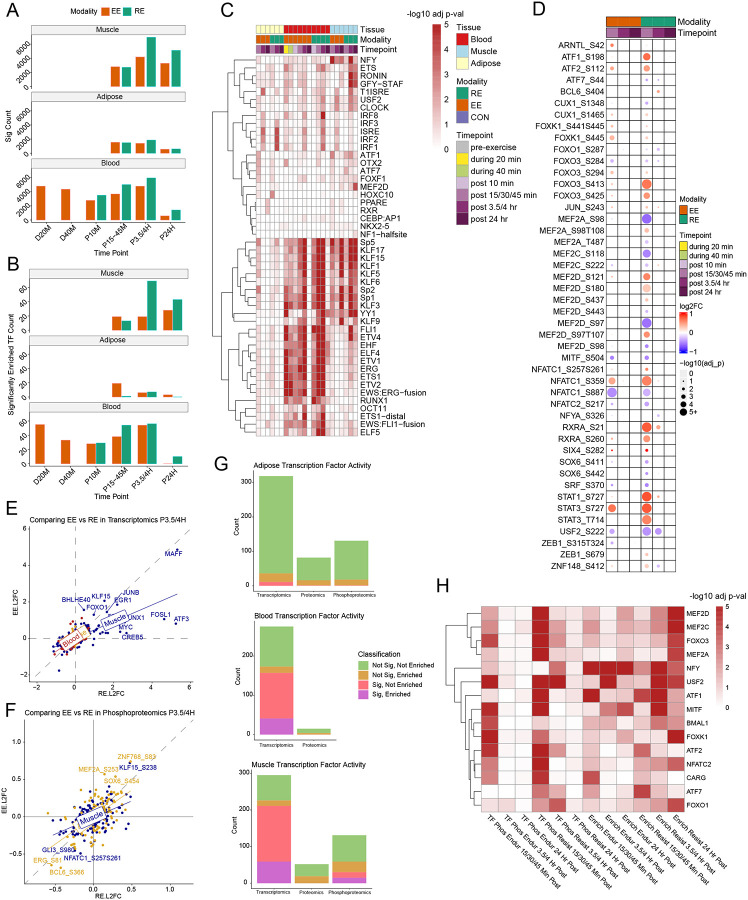
Transcription factor analysis A) Barplot of counts of significant (nominal p-val < 0.05) genes for each tissue-modality-timepoint combination used as input for transcription factor (TF) enrichment analysis. B) Barplot of counts of significantly (adjusted p-val < 0.05) enriched TFs for the inputted significant genes for each tissue, modality, timepoint combination. C) Heatmap of selected top enriched TFs for each tissue, modality and timepoint combination. Heatmap entries show −log10 adjusted p-values of TF motif enrichment and TFs are hierarchically clustered by similar enrichment significance across conditions. D) Bubble heatmap of the most significant, adjusted p-val < 0.05, TF phosphoproteomic responses to acute exercise in muscle. E-F) Scatter plots of the L2FC (log2 fold change) of transcriptomic (E) and phosphoproteomic (F) responses to EE vs RE for all three tissues, a linear model used to generate the fit line for each tissue. G) Stacked barplots for each tissue and ome reflecting the proportion of TFs with data in that tissue/ome combination that can be classified as significantly responding to acute exercise and/or significantly enriched for target genes significantly responding to acute exercise. H) Heatmap of select TFs which exhibited significant muscle phosphoproteomic responses to exercise and were enriched for target significant genes in muscle. The first six columns from the left represent the significance of the TF phosphoproteomic response to either EE or RE at each post exercise timepoint. The last six columns represent the significance of enrichment for the TF among the significant genes responding to either EE or RE at each post exercise timepoint. Endur = Endurance, Resist = Resistance.

We applied Hypergeometric Optimization of Motif EnRichment (HOMER) to the differentially expressed genes (DEGs) identified in each tissue in each exercise modality at a given timepoint relative to control.^73^ Most significant enrichments were found with the larger DEG sets in SKM and blood (Figure 6A–6B; Table S6). Based on the DEG patterns, Sp/KLF family members were among the most highly enriched TF motifs (Figure 6C). Some Sp/KLF TFs are ubiquitously expressed,^74^ and in our data were over-represented across all three tissues and at a majority of timepoints measured in the study. In contrast, interferon-stimulated response elements (ISRE) and members of the interferon regulatory family (IRF) were enriched in all tissues, primarily 3.5–4 hours post RE and EE. ISRE, IRF1, and IRF2 were the most significantly enriched TFs in AT and PPARE and RXR were specifically enriched at 24 hours post RE in AT. The circadian TF CLOCK, along with NFY, USF2, ETS, and RONIN were among a set of TFs generally enriched across tissues, with the highest significance in SKM. MEF2D, ATF1, ATF7 and FOXF1 were specifically enriched in SKM. The erythroblast transformation-specific (ETS) family of TFs, which are essential for the development and function of many innate and adaptive immune cell types^75^ were most significantly enriched in blood.

Next, we examined the transcriptomic, proteomic, and/or phosphoproteomic datasets of each tissue to identify exercise-responsive TFs. Over 200 TFs from the HOMER database were evaluated. At the transcriptome level, we noted that in any given tissue, the proportion of exercise-responsive TFs relative to all expressed TFs was higher than the proportion of exercise-responsive genes (DEGs) relative to all expressed genes: 211 of 295 identified TFs (71.52%) versus 8929 of 16509 transcripts (54.09%) in SKM, 157 of 275 identified TFs (57.09%) versus 7066 of 20526 transcripts (34.42%) in blood, and 11 of 319 identified TFs (3.45%) versus 106 of 20090 transcripts (0.53%) in AT (Supplemental Figure S6). With limited DA features in the proteomic analysis, we identified no exercise-regulated TF at the protein level in either blood or SKM, and only a single TF (E2F4) in AT. At the phosphoproteome level, we identified 47 differential phosphosites originating from 30 distinct TFs in SKM, roughly an equal proportion to the frequency of significance in all SKM measured phosphosites (22.9% TF vs 23.6% total). There was only one differential phosphosite in a TF in AT: p366 of BCL6. Significant changes in the SKM phosphoproteome are heavily skewed towards the 15 minutes post RE timepoint (Fig 6D) with multiple differential phosphorylation sites identified for FOX and MEF families of TFs. NR4A1 contains the most significant differential phosphosite at 3.5 hour post EE and RE, notably preceded by significant NR4A1 transcriptomic changes 15 minutes post EE and RE. In comparing tissue TF responses at the P3.5/4H timepoint, transcriptomic changes in SKM far outpace AT and blood and are more skewed towards RE (Figure 6E). The skew towards RE in SKM is also seen in the phosphoproteomics data, relative to AT, where the largest phosphorylation changes are greater after EE compared to RE (Figure 6F). Supplemental Table S6 includes, for each TF tested, motif enrichment significance and, where available, gene expression, protein abundance and phosphorylation significance for each measured tissue, modality and timepoint combination.

We assessed the overlap between the TFs having enriched motifs among exercise-regulated genes and the TFs regulated by exercise at either the RNA, protein, or protein phosphorylation level (Figure 6G). In total, we found 59 motif-enriched TFs in SKM, 41 in blood, and 0 in AT that showed significant RNA expression changes, whereas none of them showed significant protein level changes in any tissue. However, 15 motif-enriched TFs in SKM showed differential phosphorylation in response to exercise. As changes in phosphorylation were most likely to be associated with changes in TF activity on an acute timescale, we focused our analysis on the motif-enriched TFs presenting significant SKM phosphorylation changes. In examining those 15 TFs showing both motif enrichment in target DEGs and differential protein phosphorylation, we identified timescale patterns in TF and target activity. Phosphorylation changes tended to occur at 15 minutes post-exercise in SKM, whereas motif enrichment was skewed toward later timepoints, either 3.5 hours or 24 hours post exercise (Figure 6H). The MEF and FOX families of TFs exhibited this pattern especially with motif enrichment peaking 24 hours post exercise. This pattern was also found with USF2, MITF, the circadian TF BMAL1, and NFATC2. NFY motif enrichment was observed across all post-exercise timepoints for both exercise modalities, with a higher significance in EE than RE, and the temporal patterns of NFY phosphorylation varied between the two exercise modalities. Both the motif enrichment and phosphorylation patterns of the ATF family of TFs and CARG occurred at 15 minutes post exercise, which could denote a more rapid acting TF response.

Taken together, these analyses reveal conserved and tissue-specific TF responses to acute exercise, with SKM showing direct connections between phosphorylation changes and motif enrichment, providing insight into the temporal scales of TF activity.

### Multi-omic regulatory networks reveal TFEB as an exercise response hub

**Figure 7 -:**
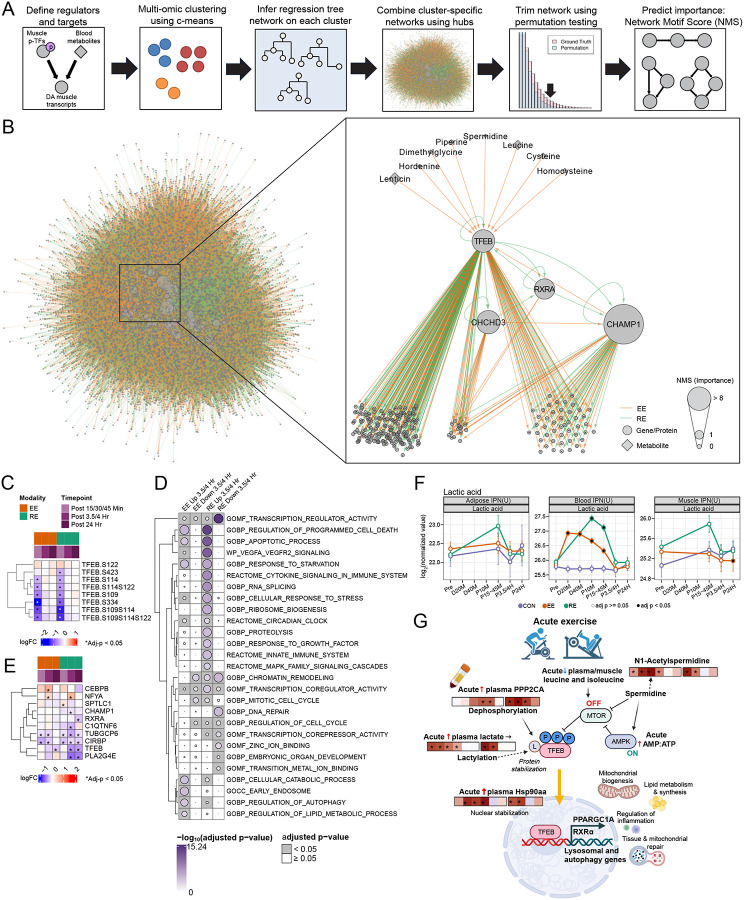
Gene regulatory network analysis A) SC-ION workflow. B) (left) Network merged across exercise modalities. (right) First-neighbor network of *TFEB*, filtered for upstream metabolites and ChIP-seq validated TFEB targets. Some relevant metabolites and targets are labeled. Size represents importance (NMS). C) Heatmap of TFEB phosphosites log fold change in SKM. D) ORA on TFEB-bound target genes in EE or RE using the C5 database. Genes were separated by fold change (up: positive, down: negative) prior to ORA. Dot plot shows selected significant terms with adjusted p-value < 0.05 in any group. E) Heatmap of RNA-seq log2 fold-change of the shared targets of *TFEB*, *CHCD3*, and *RXRA*. F) Temporal expression of lactate (quantified as lactic acid) in response to exercise across all three tissues. Data are shown as group-timepoint mean ± 95% confidence interval with a black circle indicating significance relative to control at FDR<0.05. G) Schematic illustrating the predicted multi-omics, multi-tissue TFEB signaling module in response to exercise.

Transcription factors are often key regulators of biological responses, but other types of molecules such as metabolites can also have key roles in mediating coordinated molecular signaling events and subsequent physiological responses.^76,77^ We used Spatiotemporal Clustering and Inference of Omics Networks (SC-ION)^78^ to combine blood metabolomics, SKM phosphoproteomics, and SKM transcriptomics into one multi-omics network and identify regulatory hubs (Figure 7A, STAR Methods), anticipating that circulating, small molecules drive muscular transcriptomic responses with phosphorylated TFs as mediators. Using the Network Motif Score (NMS),^78^ we identified several TFs and metabolites as important regulators, including TFs also identified by HOMER such as FOXO1, FOXO3, and NFY (Table S7). Others were not identified by HOMER such as PITX3, a protein expressed in SKM and important in myogenic regulation,^79^ and STAT5B, a key regulator of myogenic IL-15 and growth hormone signaling.^80^

In particular, we focused on TFEB, which was the 7th most important feature and a key regulator of lysosomal and autophagy genes, with recently identified roles in mitochondrial biogenesis and metabolic profiling based on murine data.^81,82^ Additionally, TFEB is in the same TF family as MITF, which was identified by HOMER as containing an enriched binding motif among SKM DEGs (Figure 6F). We filtered the predicted targets of TFEB to only those that showed prior evidence of TFEB binding via ChIP (Figure 7B; Table S7) (237/719 predicted targets, 33%)^83–86^ and found that most of these targets decreased in response to exercise at 3.5 hours post-exercise, with a stronger response in RE compared to EE (Figure S7A). One of these targets was TFEB itself, whose transcript significantly decreased 3.5 and 24 hours post-exercise in SKM (Figure S7B). Shifts in TFEB phosphorylation, specifically a modality-consistent decrease in phosphorylation at S109, S114, and S122 at 15 minutes post-exercise (Figure 7C), were used to predict its putative target genes. S122 is an mTOR substrate whose phosphorylation restricts TFEB to the cytosol^87^ and is commonly dephosphorylated by protein phosphatase 2A (PP2A).^88^ We observed an upregulation of multiple genes encoding PP2A subunits following exercise (Figure S7C), suggesting that exercise-induced PP2A may dephosphorylate TFEB and promote its nuclear translocation and transcriptional activity.^89^ Further, we observed that HSP90AA1, an upstream regulator of TFEB nuclear stabilization^90^, displayed acute transcript induction in response to both EE and RE in the blood (Figure S7D).

To build a more comprehensive picture of TFEB regulation, we performed an ORA on all of the ChIP-bound targets of TFEB (not just those predicted by SC-ION) which increased or decreased 3.5 hours post-exercise (Figure 7D). The significant terms included those related to TF activity, apoptotic process, cellular responses to starvation and stress, clock regulation and proteolysis, which are largely consistent with known roles of TFEB.^91,92^ Another notable upregulated pathway was lipid metabolism, which is consistent with reports of TFEB regulating metabolic flux during aerobic exercise^81^ and increased expression of PGC1a (*PPARGC1A)* in SKM observed at 3.5 hours post-exercise (Figure S2B). Further, we evaluated SC-ION-predicted targets regulated by TFEB and two other TFs with high network importance scores, CHCHD3 and RXRA, given their regulatory roles in mitochondrial cristae maintenance and lipid metabolism, respectively.^93,94^ This approach highlighted NFYA, a clock regulatory TF, and CEBPB, which regulates circadian autophagy,^95^ together implicating a role for TFEB in orchestrating circadian variation in cell reparative processes (Figure 7E).

We next sought to highlight putative metabolite regulators of TFEB. The branched chain amino acids, leucine, and isoleucine, decreased acutely in the blood with RE (Figure S7E), suggestive of mTOR signaling inhibition,^96^ and thus permissive TFEB activation. Spermidine, a polyamine and known upstream transcriptional regulator of TFEB,^97^ was predicted as a top regulator of TFEB. Interestingly, while the regulation of spermidine was inconsistent, we observed a modality-consistent post-exercise upregulation of the spermidine metabolite, N1-acetylspermidine (Figure S7E).^98^ Finally, lactatic acid was identified as a top candidate regulator of TFEB given its early upregulation with both modalities (Figure 7F). Lysine lactylation promotes TFEB stabilization by modifying lysine residues, thereby enhancing its nuclear retention and transcriptional activity in response to metabolic stress and autophagy.^99^ Taken together, these data build a working hypothesis that a combination of decreased mTOR and increased PP2A activity following exercise decreases TFEB phosphorylation, that, in concert with potential TFEB lactylation, allows it to enter the nucleus and activate its downstream transcriptional cascade (Figure 7G). This signaling cascade is further supported by *in vitro* observations of increased TFEB promoter activity immediately following myotube contraction.^82^

### Cellular communication network factor 1 is a novel candidate exerkine

**Figure 8 -:**
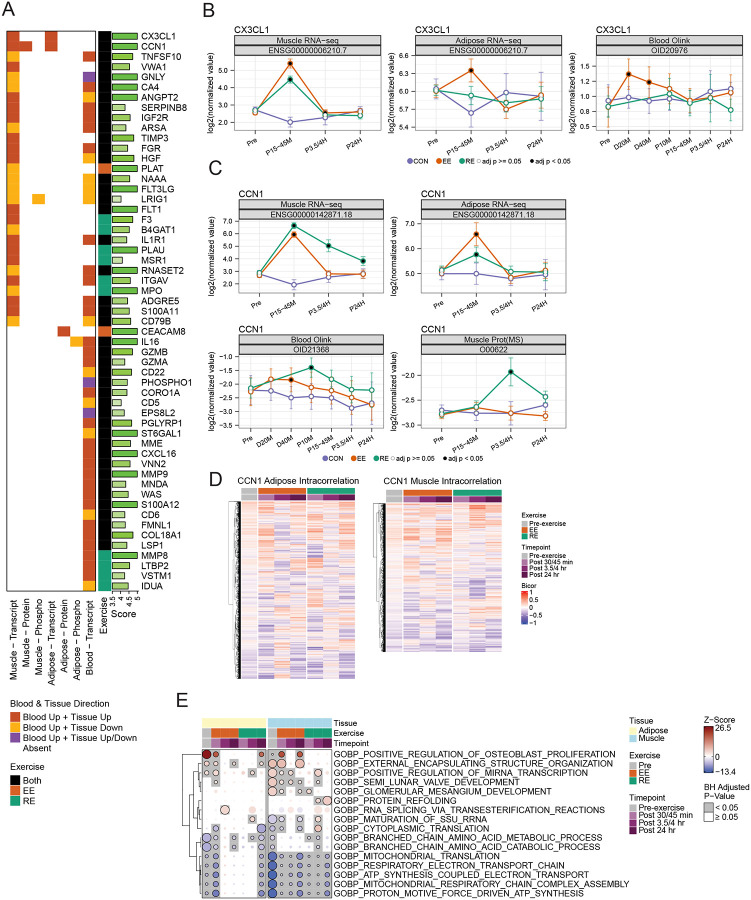
Cellular communication network factor 1 is a novel candidate exerkine A) Heatmap showing differentially regulated features across SKM, AT, and blood transcriptomics, proteomics, and phosphoproteomics (adjusted p < 0.1 at 15–45min, 3.5–4hr, or 24hr post exercise) that also exhibit significant upregulation in plasma proteomics (adjusted p < 0.1 at any timepoint, including during and post-exercise). Gene symbols from plasma Olink proteomics were used to map to corresponding features in tissue. “Blood up” indicates significant upregulation in plasma proteomics at one or more timepoints; “Tissue up” indicates significant upregulation in tissue omics at one or more timepoints. Horizontal green bars denote the extracellular score (range 0–5) using COMPARTMENTS (see Methods); only features with score > 4 are shown. “Both” in the Exercise column indicates features differentially regulated in both EE and RE. B and C) Temporal trajectories of Fractalkine (*CX3CL1*, B) and cellular communication network factor 1 (*CCN1*, C) transcript expression in AT and SKM, and protein abundance in SKM and plasma across timepoints. EE = Endurance Exercise, RE = Resistance Exercise, CON = control group, Pre = pre-exercise, D20M = during 20 minutes, D40M = during 40 minutes, P10M = 10 minutes post exercise, P15–45M = 15, 30, or 45 minutes post exercise (depending on tissue), P3.5–4H = 3.5 or 4 hours post exercise (depending on tissue), P24H = 24 hours post exercise. Data is shown as group-timepoint mean ± 95% confidence interval for the group mean with a black circle indicating model significance relative to control at adj. p-val<0.05. Plots indicate Ensembl ID for transcripts and Olink ID or Uniprot accession number for proteins. Exercise-induced changes in Fractalkine (*CX3CL1*, B) and cellular communication network factor 1 (*CCN1*, C) transcript expression in AT and SKM, and protein abundance in SKM and plasma across timepoints. Data is shown as group-timepoint mean ± 95% confidence interval with a black circle indicating significance relative to control at FDR<0.05. D: Heatmap showing intra-tissue correlation (biweight midcorrelation, r value) between *CCN1* transcript expression and all other genes in adipose and muscle. E: Gene sets associated with *CCN1* transcript expression within AT and SKM, stratified by exercise modality and timepoint. Biweight midcorrelation values were z-scaled across features, and pathway enrichment was assessed using CAMERA-PR.

To identify candidate exerkines – molecules secreted in response to an acute bout of exercise – we compared multi-omic data from SKM, AT, and blood to plasma proteomic data. To assess broadly coordinated changes, we matched any molecular changes in tissues (limited to transcriptomics, proteomics, and phosphoproteomics) to protein level changes in plasma at any time. Across all tissues, modalities, and timepoints, 168 features showed gene-matched tissue-level and plasma changes during exercise, primarily driven by transcriptomic changes during both EE and RE (Figures 8A and S8A; Table S8) in blood cells and SKM. Of these, 68% (115) were directionally concordant (i.e. both up or both down). Furthermore, 45% (n=75) demonstrated tissue changes at the same time as, or prior to, plasma changes.

Many of the observed proteins, including PXN (Figure S8B), TIMP3, and ANGPT2 (Figure 8A) regulate angiogenesis,^100–102^ while others, including interleukins and TNF superfamily proteins, are part of inflammatory or immune pathways.^103^ To distinguish likely exerkines from incidental changes in circulating plasma protein levels during exercise, the 168 candidate features were filtered to those with an extracellular compartment score greater than 4 and which actually increased in plasma with either EE or RE (Figure 8A), resulting in 55 primary candidates.^104^ All other candidates are shown in Figure S8B and Table S8.

While most genes/proteins showed changes in just one tissue-ome combination, several demonstrated multi-tissue and multi-omic changes. For example, fractalkine (*CX3CL1*), a previously described exerkine,^105^ demonstrated increased expression in SKM (RE and EE) and AT (EE only, Figure 8A and 8B). Cellular communication network factor 1 (CCN1), an angiogenic factor previously reported to be secreted by AT and SKM,^106–108^ demonstrated some of the largest changes induced by exercise. In plasma, CCN1 levels increased most noticeably at the end of the exercise bout in EE and immediately following exercise in RE (Figure 8C). Allowing for differences in measurement time between the tissues, plasma changes matched early changes in transcript levels both in AT and SKM. Effects in SKM-RE were more robust, with *CCN1* expression remaining elevated at all three post-exercise timepoints and protein changes seen at 3.5 hours post-exercise. These data suggest that both SKM and AT could contribute to circulating levels of CCN1 in exercise. Furthermore, paxillin, whose phosphorylation is promoted by CCN1,^109^ was differentially phosphorylated in both SKM and AT after exercise (Figure S8C), supporting an increase in CCN1 activity in both tissues.

Because CCN1 is also known to be produced by fibroblasts,^110^ we asked whether *CCN1* expression was correlated in SKM and AT after exercise, which might suggest the effect was not tissue specific, but rather mediated by circulating fibroblasts. While *CCN1* transcript levels were upregulated after exercise in both SKM and AT, baseline levels were not correlated among participants between the two tissues (Figure S8D). Following exercise, within-group correlations were modest and variable, suggesting that *CCN1* expression may be tissue-specific and differentially regulated depending on the exercise modality.

To examine the biological context of *CCN1* transcription within SKM and AT, we conducted gene-gene correlations between *CCN1* expression levels and the expression levels of other transcripts in each tissue before and after exercise. When evaluated across all transcripts, global correlation shifted after exercise in a modality-specific manner (Figure 8D). CAMERA-PR pathway enrichment analysis revealed that in all participants at pre-exercise, *CCN1* expression positively correlated with the expression of genes related to mRNA transcription and extracellular matrix in SKM, but was inversely correlated with mitochondrial- and metabolism-related genes in both SKM and AT (Figure 8E). Additionally, the associated biological pathways exhibited temporal and modality-specific alterations after exercise (Figure 8E). Critically, during EE, *CCN1* maintained strong associations with extracellular matrix transcripts, similar to baseline, whereas in RE this relationship was diminished, replaced by correlation to transcripts driving hypertrophy and protein refolding. Taken together, these data point to CCN1 as a novel exerkine candidate involved in ECM regulation during exercise that is connected to other structural processes in SKM during RE. These are likely angiogenic processes, given CCN1’s link to other angiogenic factors and the need for angiogenesis to support SKM growth.^111^

## Discussion

The Molecular Transducers of Physical Activity Consortium is simultaneously focused on building and sharing a molecular map of exercise response while also using that map to answer several pivotal questions about molecular responses to exercise. As such, while the present work represents a subset (pre-Covid suspension, acute exercise bout only) of the total data anticipated in MoTrPAC, we: 1) identify thousands of molecular features that change with acute bouts of EE or RE, 2) make available – both through programmatic packages and an online browser – the resulting dataset, and 3) detail novel patterns of exercise responses. To our knowledge, this represents the largest multi-tissue, multi-omic, randomized controlled trial of acute exercise available. Further, we highlight the crucial role of the non-exercising group in our data. While many multi-omic studies utilize pre-exercise samples as the only comparator, many molecular features shift over time due to fasting, circadian rhythms, and stress response from sampling; our data can be used to identify these effects.

By collecting multiple tissue specimens (blood, SKM, and ATt) from MoTrPAC participants, we were able to compare and contrast tissue-based molecular signatures of exercise. We observed that tissue responses during acute exercise, for either EE or RE, were more different than they were similar. Even restricted to just transcriptomics and metabolomics, which had balanced sample sizes across tissues, just 51 features among thousands were affected by exercise in all three tissues. Further, when temporal patterns of molecular response were analyzed using pathway enrichment, the data suggest that pathway engagement is more similar within tissue (regardless of temporal pattern) than between tissues. This is especially true at the transcript level, suggesting that established molecular pathways are largely tissue-specific, and tissues utilize elements of these pathways to respond to exercise on different timescales.

Still, some pathways were affected in multiple tissues and in both modalities, including mitochondrial pathways, VEGF and other angiogenic signalling pathways, and growth factor pathways. Given the known elements of the biological response to exercise, these pathways are expected to be engaged throughout the body. What emerged in the present data were details of the specific features and temporal responses in each tissue: for example, elements of the VEGF signaling pathway were engaged at early and intermediate timepoints post exercise in AT and blood, respectively, and across the post-exercise period in SKM.

One of the most valuable aspects of the MoTrPAC trial is the analysis and comparison of both EE and RE. While there are many shared molecular responses between EE and RE, there are also a large number of features, particularly in the blood and SKM, that are modality-specific responses. The density of MoTrPAC data meant that these effects could be resolved not just at the level of modality, but also in time. The heat shock protein transcript *HSPH1* is a clear example, peaking early in the setting of EE, but much later in RE, out to 24 hours, potentially responding to local tissue injury and inflammation in a delayed fashion. Data from MoTrPAC previously showed that Hsph1 protein abundance increased in multiple tissues during EE training of the rat.^33^ Thus heat shock responses are positioned as central regulators, not only as candidates for translating acute exercise to long term adaptation, but potentially as regulators of other key pathways, such as autophagy via TFEB.^90^

Indeed, across multiple facets of the data, the importance of autophagy and protein turnover became apparent, an effect more prominent in RE. TFEB’s role in autophagy is well described.^84,85,87,112^ Our data highlight in detail the central role that TFEB may play in activating the autophagy response in exercise. The role of autophagy and protein turnover/remodeling has been posited as a key element of the benefits conferred by exercise, particularly as a hypothetical link between exercise and cognitive benefits.^113,114^ However direct data for this link is limited. Using the present TFEB data as a map for other studies may help to identify the link between exercise and non-SKM organ health.

Understanding the communicative link between exercising SKM and other tissues is a key area of interest in the MoTrPAC study. In these data, we posit many plausible new exerkines, though *CCN1* provides a particularly striking example. We show here for the first time that CCN1 levels at the transcript and/or protein level shift in AT, SKM, and plasma. ECM remodeling and angiogenesis, pathways downstream of CCN1 and known to be critical to SKM,^111^ may extend to other tissues, though whether CCN1 is signalling in an autocrine, paracrine, or endocrine fashion is yet unclear; forthcoming rat data are likely to be informative. Interestingly, CCN1 biology is described in cardiac muscle and has been shown to respond to myocardial infarction, both in scar formation^115^ and angiogenesis.^116^ Further it has been described as a biomarker of adverse events in heart failure,^117^ presumably due to induction in response to injury. Given the present findings, it will be instructive to determine the effects of exercise on CCN1 biology in cardiovascular disease.

Our data have several important limitations. In several tissue-ome-group-timepoint buckets, the number of samples is lower than the full cohort of N = 175. This is related to a combination of temporal downsampling to limit biopsy number in participants, omic downsampling due to feasibility, assay limitations, and trial design. In some cases (< 2% of all non-epigenetic features), control group samples may be as low as N = 3. Nonetheless the paired nature of sampling (pre and post exercise) and the presence of the control group helps to resolve independent exercise effects. We anticipate validating and expanding these results when the results of the full MoTrPAC human trial are available. Further, while the sample size was among the largest in the field of acute exercise, it was still too limited to assess the effects of sex, age, or other baseline characteristics reliably. We anticipate also addressing these questions in the forthcoming larger study. Finally, our ability to cover the substantial number of effects observed in our data in the same detail given to the features described above is limited. To address this limitation, a number of companion papers explore the molecular responses in greater detail for each tissue (Ref blood, muscle, adipose papers, in preparation/submission) and for important omic classes (ref splicing paper in preparation/submission).

In sum, we provide the results of the largest multi-tissue, multi-omic trial of acute exercise, identifying a wide range of new insights to drive forward the field of exercise physiology at the molecular level. The results of the full randomized controlled trial (N = 1541), the pediatric cohort (N = 457), and the highly active study (N = 295) are forthcoming.^34,118^

## STAR Methods

### Experimental model and study participant details

#### IRB

The Molecular Transducers of Physical Activity Consortium (MoTrPAC) (NCT03960827) is a multicenter study designed to isolate the effects of structured exercise training on the molecular mechanisms underlying the health benefits of exercise and physical activity. Methods are described here in sufficient detail to interpret results, with references to supplemental material and prior publications for detailed descriptions. The present work includes data from prior to the suspension of the study in March 2020 due to the Covid-19 pandemic. The study was conducted in accordance with the Declaration of Helsinki and approved by the Institutional Review Board of Johns Hopkins University School of Medicine (IRB protocol # JHMIRB5; approval date: 05/06/2019). All MoTrPAC participants provided written informed consent for the MoTrPAC study indicating whether they agreed to open data sharing and at what level of sharing they wanted. Participants could choose to openly share all de-identified data, with the knowledge that they could be reidentified, and they could also choose to openly share limited de-identified individual level data, which is lower risk of re-identification. All analyses and resulting data and results are shared in compliance with the NIH Genomic Data Sharing (GDS) policy and DSMB requirements for the randomized study.

#### Participant Characteristics

Volunteers were screened to: (a) ensure they met all eligibility criteria; (b) acquire phenotypic assessments of the study population; and (c) verify they were healthy and medically cleared to be formally randomized into the study (cite Clinical Landscape).^34^ Participants were then randomized into intervention groups stratified by clinical site and completed a pre-intervention baseline acute test. A total of 176 participants completed the baseline acute test (EE=66, RE=73, and CON=37), and among those, 175 (99%) had at least one biospecimen sample collected. Participants then began 12 weeks of exercise training or control conditions. Upon completion of the respective interventions, participants repeated phenotypic testing and the acute test including biospecimen sampling. Due to the COVID-19 suspension, only 45 participants (26%) completed the post-intervention follow-up acute exercise test (some with less than 12 weeks of training), with 44 completing the post-intervention biospecimen collections. See *Clinical Landscape* for further detail.

#### Participant Assessments

As described in *Clinical Landscape and protocol paper*, prior to randomization, participant screening was completed via questionnaires, and measurements of anthropometrics, resting heart rate and blood pressure, fasted blood panel, cardiorespiratory fitness, muscular strength, and free-living activity and sedentary behavior.(ref Clinical landscape, in preparation/submission)^34^ Cardiorespiratory fitness was assessed using a cardiopulmonary exercise test (CPET) on a cycle ergometer (Lode Excalibur Sport Lode BV, Groningen, The Netherlands) with indirect calorimetry. Quadriceps strength was determined by isometric knee extension of the right leg at a 60° knee angle using a suitable strength device.^34^ Grip strength of the dominant hand was obtained using the Jamar Handheld Hydraulic Dynamometer (JLW Instruments, Chicago, IL). See *Clinical Landscape* for participant assessment results.

#### Acute Exercise Intervention

The pre-intervention baseline EE acute bouts (Figure 1) were composed of three parts: (1) a 5min warm up at 50% of the estimated power output to elicit 65% VO_2_peak, (2) 40min cycling at ~65% VO_2_peak, and (3) a 1min recovery at ~25 W. The pre-intervention baseline RE acute bouts were composed of a 5min warm-up at 50–60% heart rate reserve (HRR) followed by completion of three sets each of five upper (chest press, overhead press, seated row, triceps extension, biceps curl) and three lower body (leg press, leg curl, leg extension) exercises to volitional fatigue (~10RM) with 90sec rest between each set. Participants randomized to CON did not complete exercise during their acute tests. Participants rested supine for 40min to mirror the EE and RE acute test schedule. See *Clinical Landscape* for acute bout results.

#### Biospecimen Collection

To standardize conditions prior to the acute test, participants were instructed to comply with a variety of controls related to COX-inhibitors, biotin, caffeine, alcohol, exercise, and final nutrition consumption.^34^ Blood, muscle, and adipose samples were collected for the acute test at specific timepoints before, during, and after exercise (Figure 1B).

Participants arrived fasted in the morning, and rested supine for at least 30min prior to pre-exercise biospecimen collections. All participants had pre-exercise sampling from all three tissues. After the pre-exercise biospecimen collections, the subsequent collection timepoints varied for each training group and their randomized temporal profile (early, middle, late, all). Temporal profiles were used to reduce the burden of repeat sampling while still maintaining biochemical coverage of the full post-exercise period (Figure S1B, Table S1). During and after exercise, biospecimen collection varied by randomized group, tissue, and temporal group. At 20 and 40 minutes of exercise, blood was collected from only EE and CON groups due to limitations in safely obtaining blood during RE. Immediately post exercise, at 10 minutes, a blood sample was collected in all three groups. Finally, samples from all three tissues were collected at early (SKM at 15, blood at 30, and AT at 45 minutes post-exercise), intermediate (blood and SKM at 3.5, and AT at 4 hours post-exercise), and late (24 hours post-exercise in all tissues) timepoints. The post-exercise timepoint began at the completion of the 40 minutes of cycling, third set of leg extension, or 40 minutes of rest, depending on the randomized group. Except for the EE blood collection timepoints during exercise, participants rested supine or seated for biospecimen collections. The collection and processing protocols for each tissue were previously described in the human clinical protocol design paper.^34^ See *Clinical Landscape* for biospecimen collection success results.

The number of biospecimen samples that were profiled on any given assay varied by tissue, timepoint, and randomized intervention group for feasibility reasons. Multi-omic coverage is summarized in Figure 1C, Figure S1B, and Table S1.

#### Post training intervention data

As mentioned, 44 of the 175 individuals who began the trial did complete at least a portion of the training intervention period and the post-training acute bout session. These biospecimens were collected, and molecular data were generated for them. While these data are to be shared with the scientific community as detailed in the MoTrPAC Data Sharing Plan, the data are not analyzed here due to the low sample size and potential confounding between participant characteristics and groups of interest. Rather, the focus is on the baseline acute bout data. The full cohort dataset will allow exploration of multiple hypotheses related to training effects and is forthcoming.

### Molecular Quantification Methods

#### Whole Genome Sequencing (WGS)

In the present cohort, 174 of 175 participants had Illumina whole genome sequencing (WGS). DNA was extracted from cell pellets obtained from the whole blood collected in EDTA tubes using QIAamp DNA Mini Kit (QIAamp #51104) by following the manufacturer’s recommendations (Qiagen). WGS libraries were prepared using the Truseq DNA PCR-free Library Preparation Kit (Illumina # FC-121–3003) in accordance with the manufacturer’s instructions. Briefly, 1 μg of DNA was sheared using a Covaris LE220 sonicator (adaptive focused acoustics). DNA fragments underwent bead-based size selection and were subsequently end-repaired, adenylated, and ligated to Illumina sequencing adapters. Final libraries were quantified using the Qubit Fluorometer (Life Technologies) or Spectromax M2 (Molecular Devices) and Fragment Analyzer (Advanced Analytical) or Agilent 2100 BioAnalyzer. Libraries were sequenced to 30x coverage using 2×100bp paired-end run on a NovaSeq 6000 sequencer on an S4 flow cell according to the manufacturer’s instructions (Illumina). Sequenced reads were aligned to the GRCh38 reference genome (hs38DH, which includes alternative sequences, HLA regions, and decoy sequences) using the Burrows-Wheeler Aligner (BWA-MEM v0.7.15).^119^ Post-alignment processing included fixing mate pairs, base quality recalibration, and variant calling and filtration, all performed according to GATK best practices workflow that includes marking of duplicate reads by the use of Picard tools (v2.4.1),^120^ and base quality score recalibration (BQSR) via Genome Analysis Toolkit (GATK v3.5).^121,122^ Variant annotation was conducted using the Variant Effect Predictor (VEP),^123^ which provides information on variant allele frequencies from gnomAD,^124^ predicts the impact of nucleotide changes on protein sequences, and identifies variant-disease associations. Quality control of the data and variant calls were performed using Picard (CollectVariantCallingMetrics) and samtools.^120,125^ Joint genotyping was carried out on individual sample GVCFs to produce highly sensitive variant calls for the entire cohort.

#### ATAC-sequencing

ATAC-seq (Assay for Transposase Accessible Chromatin using sequencing) assays were divided by tissue, with SKM tissue assayed at Icahn School of Medicine at Mount Sinai and blood PBMCs assayed at Stanford University. Samples for ATAC PBMC processing were randomized as described in https://github.com/MoTrPAC/clinical-sample-batching. SKM samples were processed for ATAC-seq by study group (CON, EE, and RE). Eight to 12 samples were processed at a time for nuclear extraction and library preparation, and all the samples in the study group were sequenced together. For muscle, nuclei from aliquoted tissue samples (10 mg for Muscle tissues) were extracted using the Omni-ATAC protocol with modifications. Two tissue-specific consortium reference standards were included for sample processing QC. 500–600 ul 1X homogenization buffer with protease Inhibitor was added to the sample micronic tubes plus three 1.8 mm ceramic bead media. Tubes were vortexed for 5–10 seconds 3 times and kept on ice. The mixture was homogenized using Bead Ruptor Elite (Bead Mill Homogenizer) and Omni BR Cryo Cooling Unit at the settings: Tube 1.2 ml, Speed: 1.0, Cycles: 2, Time: 20 Sec, Dwell Time: 20 Sec, Temperature: 4–8°C. The homogenized mixture was filtered with Mini 20 um Pluristrainer and waited for 20–30 seconds. The homogenate tubes were centrifuged with filters attached briefly if the homogenate did not pass through the filter easily. Nuclei were stained with Trypan blue dye 0.4% and counted using Countess II Automated Cell Counter. Depending upon the counts, the number of nuclei to use for transposition was calculated. For Human Pre-covid muscle samples, 250K-300K nuclei were used. Calculated nuclei were added to 1 ml RSB in 2.0 ml Eppendorf tubes, mixed well and centrifuged at 510 RCF for 10 min on fixed angle centrifuge at 4°C. 70% supernatant was removed without disturbing pellet at the bottom and nuclei were again centrifuged at 510 RCF for 5 minutes. Almost all of the supernatant was removed. A 50 μl transposition mix was added to the pellet, and mixed by pipetting 6 times, then incubated at 37°C on a thermomixer with 1000 rpm for 30 minutes and immediately transferred to ice after incubation. The transposed DNA was purified using Zymo DNA Clean and Concentrator kit (Zymo research, D4014). The DNA product was amplified using NEBnext High-Fidelity 2x PCR Master Mix (NEB, M0541L) and custom indexed primers. Purified PCR libraries were amplified using Ampure XP beads, double-sided bead purification (sample to beads ratios 0.5X and 1.3X).

Whole blood was collected in a BD Vacutainer Cell Preparation Tube and processed for PBMC extraction and purification. Isolated PBMCs were frozen at −80°C in CoolCell Containers (Corning) and stored in liquid nitrogen. Frozen PBMCs were thawed in a 37°C water bath for 2–3 min. An aliquot of PBMC were stained with DAPI and counted using Countess III Automated Cell Counter. An aliquot of 50,000 cells was added to 1 ml of ATAC-RSB and spun at 1000 g for 10 minutes at 4°C. The supernatant was removed without disturbing the pellet. The nuclei pellet was resuspended by pipetting in 50 μL of transposition mixture (2.5μL Transposase, 25 μL 2x tagmentation buffer, 16.5 μL PBS, 0.5 μL 1% digitonin, 0.5 μL 10% Tween-20, 5 μL water) and incubated at 37°C on a thermomixer for 30 minutes with 1000 rpm shaking. The transposed DNA was purified using Qiagen MinElute Purification kits (Qiagen, 28006), and amplified using NEBnext High-Fidelity 2x PCR Master Mix (NEB, M0541L) and custom indexed primers. 90 μL SPRIselect beads (beads : DNA = 1.8) were used to purify the PCR reaction and remove excess primers and primer dimers.

#### ATAC-seq library sequencing and data processing

Pooled libraries were sequenced on an Illumina NovaSeq 6000 platform (Illumina, San Diego, CA, USA) using a paired-end 100 base-pair run configuration to a target depth of 25 million read pairs (50 million paired-end reads) per sample for muscle and to a target depth of 35 million read pairs for PBMCs. Reads were demultiplexed with bcl2fastq2 (v2.20.0) (Illumina, San Diego, CA, USA). Data was processed with the ENCODE ATAC-seq pipeline (v1.7.0) (https://github.com/ENCODE-DCC/atac-seq-pipeline). Samples from a single sex, group and exercise timepoint, e.g., endurance exercising males 30 minutes post exercise bout, were analyzed together as biological replicates in a single workflow. Briefly, adapters were trimmed with cutadapt (v2.5) and aligned to human genome hg38 with Bowtie 2 (v2.3.4.3).^126^ Duplicate reads and reads mapping to the mitochondrial chromosome were removed. Signal files and peak calls were generated using MACS2 v2.2.4,^127^ both from reads from each sample and pooled reads from all biological replicates. Pooled peaks were compared with the peaks called for each replicate individually using Irreproducibility Discovery Rate and thresholded to generate an optimal set of peaks. The cloud implementation of the ENCODE ATAC-seq pipeline and source code for the post-processing steps are available at https://github.com/MoTrPAC/motrpac-atac-seq-pipeline. Optimal peaks (overlap.optimal_peak.narrowPeak.bed.gz) from all workflows were concatenated, trimmed to 200 base pairs around the summit, and sorted and merged with bedtools v2.29.0 to generate a master peak list.^128^ This peak list was intersected with the filtered alignments from each sample using bedtools coverage with options -nonamecheck and -counts to generate a peak by sample matrix of raw counts. The remaining steps were applied separately on raw counts from each tissue. Peaks from non-autosomal chromosomes were removed, as well as peaks that did not have at least 10 read counts in six samples, which aided in increasing significant peak output in differential analysis. Principal component analysis (PCA) was conducted to find sample outliers to be excluded from downstream analysis using the *call_pca_outliers* command (found in the https://github.com/MoTrPAC/MotrpacRatTraining6mo/ R package).^33^ No PCA outliers were identified and, thus, no samples were excluded from the study. Filtered raw counts were then quantile-normalized with variancePartition::voomWithDreamWeights,^129^ which is akin to limma::voom,^130^ with the added ability to include random effects in the formula.

#### Methylation Capture sequencing

##### Data production

Plates for methylation capture were batched for methylation capture processing by exercise group. Samples were processed in subject-level batches of 4 to make 1 library pool, and all timepoints for a subject were pooled together when possible. Library pools (4 samples) were randomized into sequencing pools (40 total samples) to distribute the study groups.

Genomic DNA from muscle, adipose, and blood collected in EDTA tubes was extracted using the GenFind v3 kit (Beckman Coulter) in an automated workstation (Biomek Fxp) according to the manufacturer’s instructions. Two tissue specific control samples (provided by the Consortium) were included to monitor the sample processing QC. The DNA was quantified by Qubit assay (dsDNA BR assay, Thermo Fisher Scientific) and the quality was determined by the Nanodrop A260/280 and A260/230 ratios. A260/280 and A260/230 ratios were used to determine the quality of the DNA. DNA samples with a A260/A230 ratio above 1.8 were used for the assay.

The Methyl-Cap sequencing libraries were prepared using the Illumina TruSeq Methyl Capture EPIC Library Prep Kit (Cat# FC-151–1003) according to the manufacturer’s instructions (https://www.illumina.com/products/by-type/sequencing-kits/library-prep-kits/truseq-methyl-capture-epic.html). Briefly,1 μg of genomic DNA in 50 μL was fragmented using a Covaris E220 ultrasonicator (Covaris, USA) and the fragment size was assessed by running 1.0 μL of the samples on a Bioanalyzer High Sensitivity DNA chip (Agilent Technologies). Samples with DNA fragments between 100–300 bp with a peak between 155–175 bp were used for library preparation. Fragmented DNA was end repaired into dA-tailed fragments and ligated with custom xGen^™^ Methyl UDI-UMI Adapters (Integrated DNA Technologies). Adapter-ligated DNA was purified by magnetic bead separation. The DNA samples were then pooled in groups of 4 in equal volume (10 μL/samples, 40 μL final volume) and hybridized with Illumina EPIC probe sets (covering >3.3 million targeted CpG sites). The DNA fragments hybridized with probes were captured by streptavidin-magnetic beads and purified. The enriched DNA underwent a second round of hybridization and purification to ensure high specificity of the captured regions. Bisulfite conversion was performed on the captured DNA libraries and PCR amplified. The purified libraries were analyzed with the 2100 Bioanalyzer High Sensitivity DNA assay (Agilent Technologies) and quantified by Qubit assay (ThermoFisher). The libraries contained fragment size between 230 – 450 bp with a peak around 250 – 300 bp.

The libraries were pooled and 15% PhiX DNA (Illumina) was added to each library pool to boost diversity. DNA sequencing of paired-end reads (2 × 100 bp reads) were performed on an Illumina Novaseq 6000 system (Illumina Inc.) at a depth of approximately 60 million reads per library. In order to capture the 9-base UMIs, libraries were sequenced using 17 cycles for i7 index read and 8 cycles for the i5 index read.

##### Methylation data processing, quantification and QC

The 17 cycles for the i7 index read in the libraries consist of 8 cycles of sample i7 barcode index and 9 cycles of UMI. Using the mask options --use-bases-mask Y*,I8Y*,I*,Y* --mask-short-adapter-reads 0 --minimum-trimmed-read-length 0 in bcl2fastq command to extract the two read (R1,R2) FASTQ files, and index FASTQ files that contain the UMI information. The three FASTQ files are then fed into the methylcap processing pipeline as described in https://github.com/MoTrPAC/motrpac-methyl-capture-pipeline. Briefly, the pipeline first attaches the 9 cycles of the UMI index FASTQ file as part of the read names in both R1 and R2 FASTQ files and then uses trimgalore (via cutadapt v1.18)^131^ to remove the adapters. The FASTQ files are aligned with bismark (v0.20.0)^132^ and the resulting bam files are deduplicated using the bismark command deduplicate_bismark with option “--barcode”. The bismark coverage from the adjacent neighboring positions of the two opposite strands are merged with the bismark command coverage2cytosine with the option “--merge_CpG”.

NGScheckmate (v1.01) was applied to the deduplicated bam files from the Bismark pipeline to check the within-subject genotype consistency.^133^ The percentage of reads mapped to chrX and chrY are also checked against with the sex annotation of the sample. The inconsistent samples from genotype and sex checking and bismark QC metrics (two low read depth, two high %CHH) outliers were removed from further analysis.

For each tissue (EDTA, Muscle, Adipose) separately, the bismark coverage from all samples are combined into a single R data file after removing CpG sites with 5X coverage in less than 50% of the samples. To merge highly correlated nearby sites into a region, Markov Cluster Algorithm (MCL) is then applied on the bismark coverage R data file for each tissue separately,^33,134,135^ with the following decision algorithm: a) For each sample, compute nCpG10, the number of CpG sites with at least 10X coverage. b) Filter out samples with less than 20 percentile of the nCpG10. c) apply MCL on the CpG sites with at least 10X coverage on all of the remaining samples. d) also apply the CpG merging results from c) to the filtered out samples in b).

Principal component analysis (PCA) was conducted to find sample outliers to be excluded from downstream analysis using the *call_pca_outliers* command (found in the https://github.com/MoTrPAC/MotrpacRatTraining6mo/ R package). No additional outliers were detected from PCA in this ome.

##### Methylation Statistical analysis

To accommodate the within subject mixed effects model for the differential methylation analysis, the coverage of all of the CpG sites (or CpG regions with merged CpG sites obtained from the MCL) is applied to Malax to obtain the p-value.^136^ The statistical comparison of interest compares the change from pre-exercise to a during or post-exercise timepoint in one of the two exercise groups (RE and EE) to the same change in control. Due to the limited sample sizes in the methylation data, only sex and age are the additional covariates in the statistical model. In order to reduce the computational time, the CpG sites/regions are divided into 100 small data sets for the Malax analysis using cluster computers for parallel computing and the adjusted p-values are computed on the final merged dataset. After hypothesis testing using Malax, the change in absolute methylation percentage is calculated using edgeR.^137^

##### Transcriptomics

RNA Sequencing (RNA-Seq) was performed at Stanford University and the Icahn School of Medicine at Mount Sinai. Processing randomization for blood, muscle was done according to https://github.com/MoTrPAC/clinical-sample-batching. See below for adipose randomization considerations.

##### Extraction of total RNA

Tissues (~10 mg for muscle, ~50 mgs for adipose) were disrupted in Agencourt RNAdvance tissue lysis buffer (Beckman Coulter, Brea, CA) using a tissue ruptor (Omni International, Kennesaw, GA, #19–040E). Total RNA was extracted in a BiomekFX automation workstation according to the manufacturer’s instructions for tissue-specific extraction. Total RNA from 400 μL of blood collected in PAXgene tubes (BD Biosciences, Franklin Lakes, NJ, # 762165) was extracted using the Agencourt RNAdvance blood specific kit (Beckman Coulter). Two tissue-specific consortium reference standards were included to monitor the sample processing QC. The RNA was quantified by NanoDrop (ThermoFisher Scientific, # ND-ONE-W) and Qubit assay (ThermoFisher Scientific), and the quality was determined by either Bioanalyzer or Fragment Analyzer analysis.

##### mRNA Sequencing Library Preparation

Universal Plus mRNA-Seq kit from NuGEN/Tecan (# 9133) were used for generation of RNA-Seq libraries derived from poly(A)-selected RNA according to the manufacturer’s instructions. Universal Plus mRNA-Seq libraries contain dual (i7 and i5) 8 bp barcodes and an 8 bp unique molecular identifier (UMI), which enable deep multiplexing of NGS sequencing samples and accurate quantification of PCR duplication levels. Approximately 500ng of total RNA was used to generate the libraries for muscle, 300ng for adipose, and 250ng of total RNA was used for blood. The Universal Plus mRNA-Seq workflow consists of poly(A) RNA selection, RNA fragmentation and double-stranded cDNA generation using a mixture of random and oligo(dT) priming, end repair to generate blunt ends, adaptor ligation, strand selection, AnyDeplete workflow to remove unwanted ribosomal and globin transcripts, and PCR amplification to enrich final library species. All library preparations were performed using a Biomek i7 laboratory automation system (Beckman Coulter). Tissue-specific reference standards provided by the consortium were included with all RNA isolations to QC the RNA.

##### RNA Sequencing, quantification, and normalization

RNA sequencing, quantification, and normalization Pooled libraries were sequenced on an Illumina NovaSeq 6000 platform (Illumina, San Diego, CA, USA) to a target depth of 40 million read pairs (80 million paired-end reads) per sample using a paired-end 100 base pair run configuration. In order to capture the 8-base UMIs, libraries were sequenced using 16 cycles for the i7 index read and 8 cycles for the i5 index read. Reads were demultiplexed with bcl2fastq2 (v2.20.0) using options --use-bases-mask Y*,I8Y*,I*,Y* --mask-short-adapter-reads 0 --minimum-trimmed-read-length 0 (Illumina, San Diego, CA, USA), and UMIs in the index FASTQ files were attached to the read FASTQ files. Adapters were trimmed with cutadapt (v1.18),^126^ and trimmed reads shorter than 20 base pairs were removed. FastQC (v0.11.8) was used to generate pre-alignment QC metrics. STAR (v2.7.0d)^138^ was used to index and align reads to release 38 of the Ensembl Homo sapiens (hg38) genome and Gencode (Version 29). Default parameters were used for STAR’s genomeGenerate run mode; in STAR’s alignReads run mode, SAM attributes were specified as NH HI AS NM MD nM, and reads were removed if they did not contain high-confidence collapsed splice junctions (--outFilterType BySJout). RSEM (v1.3.1)^139^ was used to quantify transcriptome-coordinate-sorted alignments using a forward probability of 0.5 to indicate a non-strand-specific protocol. Bowtie 2 (v2.3.4.3)^140^ was used to index and align reads to globin, rRNA, and phix sequences in order to quantify the percent of reads that mapped to these contaminants and spike-ins. UCSC’s gtfToGenePred was used to convert the hg38 gene annotation (GTF) to a refFlat file in order to run Picard CollectRnaSeqMetrics (v2.18.16) with options MINIMUM_LENGTH=50 and RRNA_FRAGMENT_PERCENTAGE=0.3. UMIs were used to accurately quantify PCR duplicates with NuGEN’s “nodup.py” script (https://github.com/tecangenomics/nudup). QC metrics from every stage of the quantification pipeline were compiled, in part with multiQC (v1.6). The openWDL-based implementation of the RNA-Seq pipeline on Google Cloud Platform is available on Github (https://github.com/MoTrPAC/motrpac-rna-seq-pipeline). Filtering of lowly expressed genes and normalization were performed separately in each tissue. RSEM gene counts were used to remove lowly expressed genes, defined as having 0.5 or fewer counts per million in at least 10% of samples. These filtered raw counts were used as input for differential analysis with the variancePartition::dream,^129^ as described in the statistical analysis methods section. To generate normalized sample-level data for downstream visualization, filtered gene counts were TMM-normalized using edgeR::calcNormFactors, followed by conversion to log counts per million with edgeR::cpm.

Principal Component Analysis and calculation of the variance explained by variables of interest were used to identify and quantify potential batch effects. Based on this analysis, processing batch (muscle and blood, see below for adipose), Clinical Site (all tissues), percentage of UMI duplication, and RNA Integrity Number (RIN) technical effects were regressed out of the TMM-normalized counts via linear regression using limma::RemoveBatchEffect function in R.^141^ A design matrix including age, sex, and a combination of group and timepoint was used during batch effect removal to avoid removing variance attributable to biological effects.

##### Adipose tissue processing considerations

In the adipose tissue, RNA extraction was performed in separate batches according to exercise modality, so the extraction batch and exercise modality were perfectly collinear. This collinearity was identified at the RNA extraction step and samples were randomized prior to construction of cDNA libraries. Ultimately, under this processing implementation, differences in gene expression attributable to exercise group can be impossible to disentangle from RNA extraction batch effects, so the batch variable was not regressed out in the technical effect stage or included as a covariate in the differential analysis, and left as an experimental limitation.

##### RNA Quality Inclusion Criteria

In the blood transcriptomic data, 79/1032 processed adult-sedentary samples had RIN values under 5. Based on established guidelines and internal quality control assessments, any samples with a RIN score below 5 were excluded from further analysis due to concerns about potential degradation artifacts. Additional visualizations and summary figures supporting this decision are available in the quality control report at: https://github.com/MoTrPAC/MotrpacPreSuspensionAcute/tree/main/QC

#### Proteomics and Phosphoproteomics

##### Study design

LC-MS/MS analysis of 379 muscle samples encompassing baseline and 3 post-intervention timepoints from all three groups (control, endurance and resistance) was performed at the Broad Institute of MIT and Harvard (BI) and Pacific Northwest National Laboratories (PNNL). Samples were split evenly across the two sites, and a total of 14 samples were processed and analyzed at both sites to serve as cross-site replicates for evaluation of reproducibility. Additionally, 46 adipose tissue samples representing baseline and 4-hours post-intervention from all three groups were analyzed at PNNL.

##### Generation of common reference

For both tissue types, a tissue-specific common reference material was generated from bulk human samples. The common reference sample for muscle consisted of bulk tissue digest from 5 individuals at 2/3 ratio of female/male. Samples were split equally between BI and PNNL, digested at each site following sample processing protocol described below, then mixed all digests from both sites and centrally aliquoted at PNNL. Common reference for adipose tissue was generated at PNNL using bulk tissue from 6 individuals representing a 4:2 ratio of female:male. 250 μg aliquots of both tissue specific common reference samples were made to be included in each multiplex (described below) and additional aliquots are stored for inclusion in future MoTrPAC phases to facilitate data integration.

##### Sample processing

Proteomics analyses were performed using clinical proteomics protocols described previously.^142,143^ Muscle and adipose samples were lysed in ice-cold, freshly-prepared lysis buffer (8 M urea (Sigma-Aldrich, St. Louis, Missouri), 50 mM Tris pH 8.0, 75 mM sodium chloride, 1 mM EDTA, 2 μg/ml Aprotinin (Sigma-Aldrich, St. Louis, Missouri), 10 μg/ml Leupeptin (Roche CustomBiotech, Indianapolis, Indiana), 1 mM PMSF in EtOH, 10 mM sodium fluoride, 1% phosphatase inhibitor cocktail 2 and 3 (Sigma-Aldrich, St. Louis, Missouri), 10 mM Sodium Butyrate, 2 μM SAHA, and 10 mM nicotinamide and protein concentration was determined by BCA assay. Protein lysate concentrations were normalized within samples of the same tissue type, and protein was reduced with 5 mM dithiothreitol (DTT, Sigma-Aldrich) for 1 hour at 37°C with shaking at 1000 rpm on a thermomixer, alkylated with iodoacetamide (IAA, Sigma-Aldrich) in the dark for 45 minutes at 25°C with shaking at 1000 rpm, followed by dilution of 1:4 with Tris-HCl, pH 8.0 prior to adding digestion enzymes. Proteins were first digested with LysC endopeptidase (Wako Chemicals) at a 1:50 enzyme:substrate ratio (2 hours, 25 °C, 850 rpm), followed by digestion with trypsin (Promega) at a 1:50 enzyme:substrate ratio (or 1:10 ratio for adipose tissue; 14 hours, 25 °C, 850 rpm). The next day formic acid was added to a final concentration of 1% to quench the reaction. Digested peptides were desalted using Sep-Pac C18 columns (Waters), concentrated in a vacuum centrifuge, and a BCA assay was used to determine final peptide concentrations. 250μg aliquots of each sample were prepared, dried down by vacuum centrifugation and stored at −80°C.

Tandem mass tag (TMT) 16-plex isobaric labeling reagent (ThermoFisher Scientific) was used for this study. Samples were randomized across the first 15 channels of TMT 16-plexes, and the last channel (134N) of each multiplex was used for a common reference that was prepared prior to starting the study (see above). Randomization of samples across the plexes within each site was done using https://github.com/MoTrPAC/clinical-sample-batching, with the goal to have all timepoints per participant in the same plex, and uniform distribution of groups (endurance, resistance, control), sex and sample collection clinical site across the plexes. Peptide aliquots (250 μg per sample) were resuspended to a final concentration of 5 μg/μL in 200 mM HEPES, pH 8.5 for isobaric labeling. TMT reagent was added to each sample at a 1:2 peptide: TMT ratio, and labeling proceeded for 1 hour at 25°C with shaking at 400 rpm. The labeling reaction was diluted to a peptide concentration of 2 μg/μL using 62.5 μL of 200 mM HEPES and 20% ACN. 3 μL was removed from each sample to quantify labeling efficiency and mixing ratio. After labeling QC analysis, reactions were quenched with 5% hydroxylamine and samples within each multiplex were combined and desalted with Sep-Pac C18 columns (Waters).

Combined TMT multiplexed samples were then fractionated using high pH reversed phase chromatography on a 4.6mm ID × 250mm length Zorbax 300 Extend-C18 column (Agilent) with 5% ammonium formate/2% Acetonitrile as solvent A and 5% ammonium formate/90% acetonitrile as solvent B. Samples were fractionated with 96min separation gradient at flow rate of 1mL/min and fractions were collected at each minute onto a 96-well plate. Fractions are then concatenated into 24 fractions with the following scheme: fraction 1 = A1+C1+E1+G1, fraction 2 = A2+C2+E2+G2, fraction 3 = A3+C3+E3+G3, all the way to fraction 24 = B12+D12+F12+H12 following the same scheme. 5% of each fraction was removed for global proteome analysis, and the remaining 95% was further concatenated to 12 fractions for phosphopeptide enrichment using immobilized metal affinity chromatography (IMAC).

Phosphopeptide enrichment was performed through immobilized metal affinity chromatography (IMAC) using Fe 3+ -NTA-agarose beads, freshly prepared from Ni-NTA-agarose beads (Qiagen, Hilden, Germany) by sequential incubation in 100 mM EDTA to strip nickel, washing with HPLC water, and incubation in 10 mM iron (III) chloride). Peptide fractions were resuspended to 0.5 μg/uL in 80% ACN + 0.1% TFA and incubated with beads for 30 minutes in a thermomixer set to 1000 rpm at room temperature. After 30 minutes, beads were spun down (1 minute, 1000 rcf) and supernatant was removed and saved as flow-through for subsequent enrichments. Phosphopeptides were eluted off IMAC beads in 3x 75 μL of agarose bead elution buffer (500 mM K2HPO4, pH 7.0), desalted using C18 stage tips, eluted with 50% ACN, and lyophilized. Samples were then reconstituted in 3% ACN / 0.1% FA for LC-MS/MS analysis (9 μL reconstitution / 4 μL injection at the BI; 12 μL reconstitution / 5 μL injection at PNNL).

##### Data acquisition

###### Broad Institute:

Both proteome and phosphoproteome samples were analyzed on 75um ID Picofrit columns packed in-house with ReproSil-Pur 120 Å, C18-AQ, 1.9 μm beads to the length of 20–24cm. Online separation was performed on Easy nLC 1200 systems (ThermoFisher Scientific) with solvent A of 0.1% formic acid/3% acetonitrile and solvent B of 0.1% formic acid/90% acetonitrile, flow rate of 200nL/min and the following gradient: 2–6% B in 1min, 6–20% B in 52min, 20–35% B in 32min, 35–60% B in 9min, 60–90% B in 1min, followed by a 5min hold at 90%, and 9min hold at 50%. Proteome fractions were analyzed on a Q Exactive Plus mass spectrometer (ThrmoFisher Scientific) with MS1 scan across the 300–1800 m/z range at 70,000 resolution, AGC target of 3×10^6^ and maximum injection time of 5ms. MS2 scans of most abundant 12 ions were performed at 35,000 resolution with AGC target of 1×10^5^ and maximum injection time of 120ms, isolation window of 0.7m/z and normalized collision energy of 27. Phosphoproteome fractions were analyzed on Q Exactive HFX MS system (ThermoFisher Scientific) with the following parameters: MS1 scan across 350–1800 m/z mass range at 60,000 resolution, AGC target of 3×10^6^ and maximum injection time of 10ms. 20 most abundant ions are fragmented in MS2 scans with AGC of 1×10^5^, maximum injection time of 105ms, isolation width of 0.7 m/z and NCE of 29. In both methods dynamic exclusion was set to 45sec.

###### PNNL:

For mass spectrometry analysis of the global proteome of muscle samples, online separation was performed using a nanoAcquity M-Class UHPLC system (Waters) and a 25 cm × 75 μm i.d. picofrit column packed in-house with C18 silica (1.7 μm UPLC BEH particles, Waters Acquity) with solvent A of 0.1% formic acid/3% acetonitrile and solvent B of 0.1% formic acid/90% acetonitrile, flow rate of 200nL/min and the following gradient: 1% B for 8min, 8–20% B in 90min, 20–35% B in 13min, 35–75% B in 5min, 75–95% B in 3min, followed by a 6min hold at 90%, and 9min hold at 50%. Proteome fractions were analyzed on a Q Exactive Plus mass spectrometer (ThermoFisher Scientific) with MS1 scan across the 300–1800 m/z range at 60,000 resolution, AGC target of 3×10^6^ and maximum injection time of 20ms. MS2 scans of most abundant 12 ions were performed at 30,000 resolution with AGC target of 1×10^5^ and maximum injection time of 100ms, isolation window of 0.7m/z and normalized collision energy of 30.

For mass spectrometry analysis of the global proteome of adipose samples, online separation was performed using a Dionex Ultimate 3000 UHPLC system (ThermoFisher) and a 25 cm × 75 μm i.d. picofrit column packed in-house with C18 silica (1.7 μm UPLC BEH particles, Waters Acquity) with solvent A of 0.1% formic acid/3% acetonitrile and solvent B of 0.1% formic acid/90% acetonitrile, flow rate of 200nL/min and the following gradient: 1–8% B in 10min, 8–25% B in 90min, 25–35% B in 10min, 35–75% B in 5min, and 75–5% in 3min. Proteome fractions were analyzed on a Q Exactive HF-X Plus mass spectrometer (ThermoFisher Scientific) with MS1 scan across the 300–1800 m/z range at 60,000 resolution, AGC target of 3×10^6^ and maximum injection time of 20ms. MS2 scans of most abundant 12 ions were performed at 30,000 resolution with AGC target of 1×10^5^ and maximum injection time of 100ms, isolation window of 0.7m/z and normalized collision energy of 30.

For mass spectrometry analysis of the phosphoproteome of both sample types, online separation was performed using a Dionex Ultimate 3000 UHPLC system (ThermoFisher) and a 25 cm × 75 μm i.d. picofrit column packed in-house with C18 silica (1.7 μm UPLC BEH particles, Waters Acquity) with solvent A of 0.1% formic acid/3% acetonitrile and solvent B of 0.1% formic acid/90% acetonitrile, flow rate of 200nL/min and the following gradient: 1–8% B in 10min, 8–25% B in 90min, 25–35% B in 10min, 35–75% B in 5min, and 75–5% in 3min. Phosphoproteome fractions were analyzed on a Q Exactive HF-X Plus mass spectrometer (ThermoFisher Scientific) with MS1 scan across the 300–1800 m/z range at 60,000 resolution, AGC target of 3×10^6^ and maximum injection time of 20ms. MS2 scans of most abundant 12 ions were performed at 45,000 resolution with AGC target of 1×10^5^ and maximum injection time of 100ms, isolation window of 0.7m/z and normalized collision energy of 30.

##### Data searching

Proteome and phosphoproteome data from both BI and PNNL were searched against a composite protein database at the Bioinformatics Center (BIC) using the MSGF+ cloud-based pipeline previously described.^144^ This database comprised UniProt canonical sequences (downloaded 2022-09-13; 20383 sequences), UniProt human protein isoforms (downloaded 2022-09-13; 21982 sequences), and common contaminants (261 sequences), resulting in 42,626 sequences.

##### QC/Filtering/Normalization

The log2 Reporter ion intensity (RII) ratios to the common reference were used as quantitative values for all proteomics features (proteins and phosphosites). Datasets were filtered to remove features identified from contaminant proteins and decoy sequences. Datasets were visually evaluated for sample outliers by looking at top principal components, examining median feature abundance and distributions of RII ratio values across samples, and by quantifying the number of feature identifications within each sample. No outliers were detected in the muscle tissue dataset. In the adipose datasets (proteome and phosphoproteome), four samples were flagged as outliers based on inspection of the top principal components and evaluation of the raw quantitative results (median feature abundance <-1.0). These outlier samples were removed from the dataset, and the list of outlier samples can be found in Supplementary Table S1. The Log_2_ RII ratio values were normalized within each sample by median centering to zero. Principal Component Analysis and calculation of the variance explained by variables of interest were used to identify and quantify potential batch effects. Based on this analysis, TMT plex (muscle and adipose), Clinical Site (muscle and adipose), and Chemical Analysis Site (muscle only, where samples were analyzed at both BI and PNNL) batch effects were removed using Linear Models Implemented in the *limma::RemoveBatchEffect()* function in R.^141^ A design matrix including age, sex, and group_timepoint was used during batch effect removal in order to preserve the effect of all variables included in later statistical analysis. Correlations between technical replicates analyzed within and across CAS (where applicable) were calculated to evaluate intra- and inter-site reproducibility; the data from technical replicates were then averaged for downstream analysis. Finally, features with quantification in less of 30% of all samples were removed. For specific details of the process, see available code.

##### Affinity Proteomics

Plasma proteomics profiling was performed using the Olink (Uppsala, Sweden) assay. The Proximity Extension Assay (PEA) technology has been described.^145^ Briefly, a ~20μL plasma sample is first incubated with two distinct antibodies that bind proximal epitopes on the protein target. The two antibodies are conjugated with complementary DNA oligonucleotide sequences, which come in close proximity upon target binding and subsequently hybridize (immunoreaction). The sequence is then extended by DNA polymerase, creating a unique amplicon (i.e., barcode) for each individual protein, which is then amplified by polymerase chain reaction. Every sample is spiked with three internal quality controls: (1) “Immuno controls” are non-human antigens measured by Olink that assess technical variation in all three steps of the reaction; (2) “extension controls” are composed of an antibody coupled to a unique pair of DNA-tags that are always in proximity, to produce a constant signal independent of the immunoreaction. This control is used to adjust the signal from each sample with respect to extension and amplification; (3) “amplification/detection” control is a complete double-stranded DNA amplicon that does not require either proximity binding or extension to generate a signal, specifically monitoring only the amplification/detection step. Additionally, eight external controls are added to separate wells on each sample plate. Two pooled plasma or “external sample” controls, generated by pooling several μL of plasma from samples from healthy volunteers, are used to assess potential variation between runs and plates (i.e. to calculate inter-assay and intra-assay CVs). Three negative controls (buffer only) per plate are used to monitor background noise generated when DNA-tags come in close proximity without prior binding to the appropriate protein. These negative controls set the background level for each protein assay in order to calculate the limit of detection (LOD). Finally, each plate includes a Plate Control (PC) of a separate pooled EDTA plasma sample plated in triplicate; the median of the PC triplicates is used to normalize each assay and to compensate for potential variation between runs and plates. Data values for measurements below LOD are reported for all samples.

In total, 378 MoTrPAC sedentary adult samples were assayed across 9 plates in the same batch. No samples failed Olink standard quality control and median intra-assay and inter-assay CVs were 14.5% and 30.2%, respectively. Principal component analysis was performed and demonstrated minimal effects by plate and clinical site. Sample outliers were identified as those >3x the interquartile range for at least one of the first three principal components. Four samples (1.1%) from 3 different individuals were flagged as potential outliers and excluded from analyses. Final data are presented as NPX (normalized protein expression) values, Olink’s relative protein quantification unit on a log2 scale.

##### Metabolomics and Lipidomics

The metabolomic analysis was performed by investigators across multiple *Chemical Analysis Sites* that employed different technical platforms for data acquisition. At the highest level, these platforms were divided into two classes: *Targeted* and *Untargeted*. The data generated by the untargeted platforms were further divided into Named (confidently identified chemical entities) and Unnamed (confidently detected, but no chemical name annotation) compounds.

Targeted metabolomics data were generated at 3 analysis Sites: Duke University, the Mayo Clinic, and Emory University. Duke quantified metabolites belonging to the metabolite classes Acetyl-CoA, Keto Acids, and Nucleic Acids (“acoa”, “ka”, “nuc”), Mayo quantified amines and TCA intermediates (“amines” and “tca”), while Emory quantified Oxylipins (“oxylipneg”).

The untargeted metabolomics data were generated at 3 analysis Sites: the Broad Institute, the University of Michigan, and the Georgia Institute of Technology (Georgia Tech). The Broad Institute applied Hydrophilic interaction liquid chromatography (HILIC) in the positive ion mode (hilicpos), Michigan applied reverse phase liquid chromatography in both positive and negative ion modes (“rppos” and “rpneg”) and ion-pairing chromatography in the negative mode (“ionpneg”), and Georgia Tech performed lipidomics assays using reverse phase chromatography in both positive and negative ion modes (“lrppos” and “lrpneg”).

##### LC-MS/MS analysis of branched-chain keto acids

Duke University conducted targeted profiling of branched-chain keto acid metabolites. Plasma samples containing isotopically labeled ketoleucine (KIC)-d3, ketoisovalerate (KIV)-^13^C_5_ (from Cambridge Isotope Laboratories), and 3-methyl-2-oxovalerate (KMV)-d8 (from Toronto Research Chemicals, Canada) internal standards, were subjected to deproteinization with 3M perchloric acid. Tissue homogenates were prepared at 100 mg/ml in 3M perchloric acid and 200 μL was centrifuged.

Next, 200 μL of 25 M o-phenylenediamine (OPD) in 3M HCl were added to both the plasma and tissue supernatants. The samples were incubated at 80°C for 20 minutes. Keto acids were then extracted using ethyl acetate, following a previously described protocol.^146,147^ The extracts were dried under nitrogen, reconstituted in 200 mM ammonium acetate, and subjected to analysis on a Xevo TQ-S triple quadrupole mass spectrometer coupled to an Acquity UPLC (Waters), controlled by the MassLynx 4.1 operating system.

The analytical column used was a Waters Acquity UPLC BEH C18 Column (1.7 μm, 2.1 × 50 mm), maintained at 30°C. 10 μL of the sample were injected onto the column and eluted at a flow rate of 0.4 ml/min. The gradient consisted of 45% mobile phase A (5 mM ammonium acetate in water) and 55% mobile phase B (methanol) for 2 minutes. This was succeeded by a linear gradient to 95% B from 2 to 2.5 minutes, holding at 95% B for 0.7 minutes, returning to 45% A, and finally re-equilibrating the column at initial conditions for 1 minute. The total run time was 4.7 minutes.

In positive ion mode, mass transitions of m/z 203 → 161 (KIC), 206 → 161 (KIC-d3), 189 → 174 (KIV), 194 → 178 (KIV-^5^C_13_), 203 → 174 (KMV), and 211 → 177 (KMV-d8) were monitored. Endogenous keto acids were quantified using calibrators prepared by spiking dialyzed fetal bovine serum with authentic keto acids (Sigma-Aldrich).

##### Flow injection MS/MS analysis of acyl CoAs

Duke University conducted targeted profiling of acyl CoAs. Here, 500 μL of tissue homogenate, prepared at a concentration of 50 mg/ml in isopropanol/0.1 M KH2PO4 (1:1), underwent extraction with an equal volume of acetonitrile. The resulting mixture was then centrifuged at 14,000 × g for 10 minutes, following a previously established procedure.^148,149^

The supernatants were acidified with 0.25 ml of glacial acetic acid, and the acyl CoAs were subjected to additional purification through solid-phase extraction (SPE) using 2-(2-pyridyl) ethyl functionalized silica gel (Sigma-Aldrich).^150^ Prior to use, the SPE columns were conditioned with 1 ml of acetonitrile/isopropanol/water/glacial acetic acid (9/3/4/4 : v/v/v/v). After application and flow-through of the supernatant, the SPE columns underwent a washing step with 2 ml of acetonitrile/isopropanol/water/glacial acetic acid (9/3/4/4 : v/v/v/v). Acyl CoAs elution was achieved with 2 ml of methanol/250 mM ammonium formate (4/1 : v/v), followed by analysis using flow injection MS/MS in positive ion mode on a Xevo TQ-S triple quadrupole mass spectrometer (Waters). The mobile phase used was methanol/water (80/20, v/v) with 30 mM ammonium hydroxide. Spectra were acquired in the multichannel acquisition mode, monitoring the neutral loss of 507 amu (phosphoadenosine diphosphate) and scanning from m/z 750 to 1100. As an internal standard, heptadecanoyl CoA was employed.

Quantification of endogenous acyl CoAs was carried out using calibrators created by spiking tissue homogenates with authentic Acyl CoAs (Sigma-Aldrich) that covered saturated acyl chain lengths from C0 to C18. Empirical corrections for heavy isotope effects, particularly ^13^C, to the adjacent m+2 spectral peaks in a specific chain length cluster were made by referring to the observed spectra for the analytical standards.

##### LC-MS/MS analysis of nucleotides

Duke University conducted targeted profiling of nucleotide metabolites. 300 μL of tissue homogenates, prepared at a concentration of 50 mg/ml in 70% methanol, underwent spiking with nine internal standards: ^13^C^10^,^15^N^5^-adenosine monophosphate, ^13^C^10^,^15^N^5^-guanosine monophosphate, ^13^C^10^,^15^N^2^-uridine monophosphate, ^13^C^9^,^15^N^3^-cytidine monophosphate, ^13^C^10^-guanosine triphosphate, ^13^C^10^-uridine triphosphate, ^13^C^9^-cytidine triphosphate, ^13^C^10^-adenosine triphosphate, and nicotinamide-1, N^6^-ethenoadenine dinucleotide (eNAD) (all from Sigma-Aldrich).

Nucleotides were extracted using an equal volume of hexane, following the procedures previously outlines.^151,152^ After vortexing and centrifugation at 14,000 × g for 5 minutes, the bottom layer was subjected to another centrifugation. Chromatographic separation and MS analysis of the supernatants were performed using an Acquity UPLC system (Waters) coupled to a Xevo TQ-XS quadrupole mass spectrometer (Waters).

The analytical column employed was a Chromolith FastGradient RP-18e 50–2mm column (EMD Millipore, Billerica, MA, USA), that was maintained at 40°C. The injection volume was 2 μL. Nucleotides were separated using a mobile phase A consisting of 95% water, 5% methanol, and 5 mM dimethylhexylamine adjusted to pH 7.5 with acetic acid. Mobile phase B comprised 20% water, 80% methanol, and 10 mM dimethylhexylamine. The flow rate was set to 0.3 ml/min. The 22-minute gradient (t=0, %B=0; t=1.2, %B=0; t=22, %B=40) was followed by a 3-minute wash and 7-minute equilibration. Nucleotides were detected in the negative ion multiple reaction monitoring (MRM) mode based on characteristic fragmentation reactions. Endogenous nucleotides were quantified using calibrators prepared by spiked-in authentic nucleotides obtained from Sigma-Aldrich.

##### LC-MS/MS analysis of amino metabolites

The Mayo Clinic conducted LC-MS-based targeted profiling of amino acids and amino metabolites, as previously outlined.^153,154^ In summary, either 20 ml of plasma samples or 5 mg of tissue homogenates were supplemented with an internal standard solution comprising isotopically labeled amino acids (U-^13^C^4^ L-aspartic acid, U-^13^C_3_ alanine, U-^13^C_4_ L-threonine, U-^13^C L-proline, U-^13^C_6_ tyrosine, U-^13^C_5_ valine, U-^13^C_6_ leucine, U-^13^C_6_ phenylalanine, U-^13^C_3_ serine, U-^13^C_5_ glutamine, U-^13^C_2_ glycine, U-^13^C_5_ glutamate, U-^13^C_6_,^15^N_2_ lysine, U-^13^C_5_,^15^N methionine, 1,1U-^13^C_2_ homocysteine, U-^13^C_6_ arginine, U-^13^C_5_ ornithine, ^13^C_4_ asparagine, ^13^C_2_ ethanolamine, D_3_ sarcosine, D_6_ 4-aminobutyric acid).

The supernatant was promptly derivatized using 6-aminoquinolyl-N-hydroxysuccinimidyl carbamate with a MassTrak kit (Waters). A 10-point calibration standard curve underwent a similar derivatization procedure after the addition of internal standards. Both the derivatized standards and samples were subjected to analysis using a Quantum Ultra triple quadrupole mass spectrometer (ThermoFischer) coupled with an Acquity liquid chromatography system (Waters). Data acquisition utilized selected ion monitoring (SRM) in positive ion mode. The concentrations of 42 analytes in each unknown sample were calculated against their respective calibration curves.

##### GC-MS analysis of TCA metabolites

The Mayo Clinic conducted GC-MS-targeted profiling of tricarboxylic acid (TCA) metabolites, as previously detailed,^155,156^ with some modifications. In summary, 5 mg of tissue were homogenized in 1X PBS using an Omni bead ruptor (Omni International, Kennesaw, GA), followed by the addition of 20 μL of an internal solution containing U-^13^C labeled analytes (^13^C_3_ sodium lactate, ^13^C_4_ succinic acid, ^13^C_4_ fumaric acid, ^13^C_4_ alpha-ketoglutaric acid, ^13^C_4_ malic acid, ^13^C_4_ aspartic acid, ^13^C_5_ 2-hydroxyglutaric acid, ^13^C_5_ glutamic acid, ^13^C_6_ citric acid, ^13^C_2_,^15^N glycine, ^13^C_2_ sodium pyruvate). For plasma, 50 μL were used.

The proteins were precipitated out using 300 μL of a mixture of chilled methanol and acetonitrile solution. After drying the supernatant in the speedvac, the sample was derivatized first with ethoxime and then with MtBSTFA + 1% tBDMCS (N-Methyl-N-(t-Butyldimethylsilyl)-Trifluoroacetamide + 1% t-Butyldimethylchlorosilane) and then analyzed on an Agilent 5977B GC/MS (Santa Clara, California) under single ion monitoring conditions using electron ionization. Concentrations of lactic acid (m/z 261.2), fumaric acid (m/z 287.1), succinic acid (m/z 289.1), ketoglutaric acid (m/z 360.2), malic acid (m/z 419.3), aspartic acid (m/z 418.2), 2-hydroxyglutaratic acid (m/z 433.2), cis-aconitic acid (m/z 459.3), citric acid (m/z 591.4), isocitric acid (m/z 591.4), and glutamic acid (m/z 432.4) were measured against 7-point calibration curves that underwent the same derivatization procedure.

##### Targeted lipidomics of low-level lipids

Lipid targeted profiling was conducted at Emory University following established methodologies as previously described.^157,158^ In summary, 20 mg of powdered tissue samples were homogenized in 100 μL PBS using Bead Ruptor (Omni International, Kennesaw, GA). Homogenized tissue samples (or plasma samples) were diluted with 300 μL 20% methanol and spiked with a 1% BHT solution to a final BHT concentration of 0.1% and pH of 3.0 by acetic acid addition. After centrifugation (10 minutes, 14000 rpm), the supernatants were transferred to 96-well plates for further extraction.

The supernatants were loaded onto Isolute C18 solid-phase extraction (SPE) columns that had been conditioned with 1000 μL ethyl acetate and 1000 μL 5% methanol. The SPE columns were washed with 800 μL water and 800 μL hexane, and the oxylipins were then eluted with 400 μL methyl formate. The SPE process was automated using a Biotage Extrahera (Uppsala, Sweden). The eluate was dried with nitrogen and reconstituted with 200 μL acetonitrile before LC-MS analysis. Sample blanks, pooled extract samples used as quality controls (QC), and consortium reference samples were prepared for analysis using the same methods. All external standards were purchased from Cayman Chemical (Ann Arbor, Michigan) at a final concentration in the range 0.01–20 μg/ml and consisted of: prostaglandin E2 ethanolamide (Catalog No. 100007212), oleoyl ethanolamide (Catalog No. 90265), palmitoyl ethanolamide (Catalog No. 10965), arachidonoyl ethanolamide (Catalog No. 1007270), docosahexaenoyl ethanolamide (Catalog No. 10007534), linoleoyl ethanolamide (Catalog No. 90155), stearoyl ethanolamide (Catalog No. 90245), oxy-arachidonoyl ethanolamide (Catalog No. 10008642), 2-arachidonyl glycerol (Catalog No. 62160), docosatetraenoyl ethanolamide (Catalog No. 90215), α-linolenoyl ethanolamide (Catalog No. 902150), oleamide (Catalog No. 90375), dihomo-γ-linolenoyl ethanolamide (Catalog No. 09235), decosanoyl ethanolamide (Catalog No. 10005823), 9,10 DiHOME (Catalog No. 53400), prostaglandin E2–1-glyceryl ester (Catalog No. 14010), 20-HETE (Catalog No. 10007269), 9-HETE (Catalog No. 34400), 14,15 DiHET (Catalog No. 10007267), 5(S)-HETE (Catalog No. 34210), 12(R)-HETE (Catalog No. 10007247), 11(12)-DiHET (Catalog No. 10007266), 5,6-DiHET (Catalog No. 10007264), thromboxane B2 (Catalog No. 10007237), 12(13)-EpOME (Catalog No. 52450), 13 HODE (Catalog No. 38600), prostaglandin F2α (Catalog No. 10007221), 14(15)-EET (Catalog No. 10007263), 8(9)-EET (Catalog No. 10007261), 11(12)-EET (Catalog No. 10007262), leukotriene B4 (Catalog No. 20110), 8(9)-DiHET (Catalog No. 10007265), 13-OxoODE (Catalog No. 38620), 13(S)-HpODE (Catalog No. 48610), 9(S)-HpODE (Catalog No. 48410), 9(S)-HODE (Catalog No. 38410), resolvin D3 (Catalog No. 13834), resolvin E1 (Catalog No. 10007848), resolvin D1 (Catalog No. 10012554), resolvin D2 (Catalog No. 10007279), 9(S)HOTrE (Catalog No. 39420), 13(S)HOTrE (Catalog No. 39620), 8-iso Progstaglandin F2α (Catalog No. 25903), maresin 1 (Catalog No. 10878), maresin 2 (Catalog No. 16369).

LC-MS data were acquired using an Agilent 1290 Infinity II chromatograph from Agilent (Santa Clara, CA), equipped with a ThermoFisher Scientific AccucoreTM C18 column (100 mm × 4.6, 2.6 μm particle size), and coupled to Agilent 6495 mass spectrometer for polarity switch multiple reaction monitoring (MRM) scan. The mobile phases consisted of water with 10 mM ammonium acetate (mobile phase A) and acetonitrile with 10 mM ammonium acetate (mobile phase B). The chromatographic gradient program was: 0.5 minutes with 95% A; 1 minute to 2 minutes with 65% A; 2.1 minutes to 5.0 minutes with 45% A; 7 minutes to 20 minutes with 25% A; and 21.1 minutes until 25 minutes with 95% A. The flow rate was set at 0.40 ml/min. The column temperature was maintained at 35°C, and the injection volume was 6 μL. For MS analysis, the MRM analysis is operated at gas temperature of 290 °C, gas flow of 14 L/min, Nebulizer of 20 psi, sheath gas temperature of 300 °C, sheath gas flow of 11 L/min, capillary of 3000 V for both positive and negative ion mode, nozzle voltage of 1500 V for both positive and negative ion mode, high pressure RF of iFunnel parameters of 150V for both positive and negative ion mode, and low pressure of RF of iFunnel parameters of 60 V for both positive and negative ion mode. Skyline (version 25.1.0.142)^159^ was utilized to process raw LC-MS data. Standard curves were constructed for each oxylipin/ethanolamide and scrutinized to ensure that all concentration points fell within the linear portion of the curve with an R-squared value not less than 0.9. Additionally, features exhibiting a high coefficient of variation (CV) among the quality control (QC) samples were eliminated from the dataset. Pearson correlation among the QCs for each tissue type was computed using the Hmisc R library, and the figures documented in the QC report were generated and visualized with the corrplot R library.^160,161^

##### Hydrophilic interaction LC-MS metabolomics

The untargeted analysis of polar metabolites in the positive ionization mode was conducted at the Broad Institute of MIT and Harvard. The LC-MS system consisted of a Shimadzu Nexera X2 UHPLC (Shimadzu Corp., Kyoto, Japan) coupled to a Q-Exactive hybrid quadrupole Orbitrap mass spectrometer (Thermo Fisher Scientific). Plasma samples (10 μL) were extracted using 90 μL of 74.9/24.9/0.2 v/v/v acetonitrile/methanol/formic acid containing valine-d8 and phenylalanine-d8 internal standards. Following centrifugation, the supernatants were directly injected onto a 150 × 2 mm, 3 μm Atlantis HILIC column (Waters). Tissue (10 mg) homogenization was performed at 4°C using a TissueLyser II (QAIGEN) bead mill set to two 2 min intervals at 20 Hz in 300 μL of 10/67.4/22.4/0.018 v/v/v/v water/acetonitrile/methanol/formic acid valine-d8 and phenylalanine-d8 internal standards. The column was eluted isocratically at a flow rate of 250 μL/min with 5% mobile phase A (10 mM ammonium formate and 0.1% formic acid in water) for 0.5 minute, followed by a linear gradient to 40% mobile phase B (acetonitrile with 0.1% formic acid) over 10 minutes, then held at 40% B for 4.5 minutes. MS analyses utilized electrospray ionization in the positive ion mode, employing full scan analysis over 70–800 m/z at 70,000 resolution and 3 Hz data acquisition rate. Various MS settings, including sheath gas, auxiliary gas, spray voltage, and others, were specified for optimal performance. Data quality assurance was performed by confirming LC-MS system performance with a mixture of >140 well-characterized synthetic reference compounds, daily evaluation of internal standard signals, and the analysis of four pairs of pooled extract samples per sample type inserted in the analysis queue at regular intervals. One sample from each pair was used to correct for instrument drift using “nearest neighbor” scaling while the second reference sample served as a passive QC for determination of the analytical coefficient of variation of every identified metabolite and unknown. Raw data processing involved the use of TraceFinder software (v3.3, Thermo Fisher Scientific) for targeted peak integration and manual review, as well as Progenesis QI (v3.0, Nonlinear Dynamics, Waters) for peak detection and integration of both identified and unknown metabolites. Metabolite identities were confirmed using authentic reference standards.

##### Reversed Phase-High Performance & Ion-pairing LC metabolomics

Reversed-phase and ion pairing LC-MS profiling of polar metabolites was conducted at the University of Michigan. LC-MS grade solvents and mobile phase additives were procured from Sigma-Aldrich, while chemical standards were obtained from either Sigma-Aldrich or Cambridge Isotope Labs. Plasma (50 μL aliquot) was extracted by addition of 200 μL of 1:1:1 v:v methanol:acetonitrile:acetone containing the following internal standards diluted from stock solutions to yield the specified concentrations: D_4_-succinic acid, 12.5 μM; :D_3_-malic acid,12.5 μM; D_5_ -glutamic acid,12.5 μM; D_10_ -leucine,12.5 μM; D_5_-tryptophan,12.5 μM;D_5_-phenylalanine,12.5 μM; D_3_-caffeine,12.5 μM; D_8_-lysine,12.5 μM; D_4_-chenodeoxycholic acid,12.5 μM; phosphatidylcholine(17:0/17:0),2.5 μM; phosphatidylcholine(19:0/19:0),2.5 μM; D_31_-palmitic acid,12.5 μM; D_35_-stearic acid,12.5 μM; gibberellic acid, 2.5 μM; epibrassinolide, 2.5 μM. The extraction solvent also contained a 1:400 dilution of Cambridge Isotope carnitine/acylcarnitine mix NSK-B, resulting in the following concentrations of carnitine/acylcarnitine internal standards: D_9_-L-carnitine, 380 nM; D_3_-L-acetylcarnitine, 95 nM; D_3_-L-propionylcarnitine, 19 nM; D_3_-L-butyrylcarnitine, 19 nM; D_9_-L-isovalerylcarnitine, 19 nM; D_3_-L-octanoylcarnitine, 19 nM; D_9_-L-myristoylcarnitine, 19 nM; D_3_-L-palmitoylcarnitine, 38 nM. After addition of solvent, samples were vortexed, incubated on ice for 10 minutes, and then centrifuged at 15,000 × g for 5 minutes. 150 μL supernatant were retrieved from the pellet, transferred to a glass autosampler vial with a 250 uL footed insert, and dried under a constant stream of nitrogen gas at ambient temperature. A QC sample was created by pooling residual supernatant from multiple samples. This QC sample underwent the same drying and reconstitution process as described for individual samples. Dried supernatants were stored at −80 C until ready for instrumental analysis. On the day of analysis, dried samples were reconstituted in 37.5 μL of 8:2 v:v water:methanol, vortexed thoroughly, and submitted for LC-MS.

Frozen tissue samples (skeletal muscle and adipose) were rapidly weighed into pre-tared, pre-chilled Eppendorf tubes and extracted in 1:1:1:1 v:v methanol:acetonitrile:acetone:water containing the following internal standards diluted from stock solutions to yield the specified concentrations: ^13^C_3_-lactic acid, 12.5 μM; ^13^C_5_-oxoglutaric acid, 125 nM;^13^C_5_-citric acid,1.25 μM; ^13^C_4_-succinic acid,125 nM; ^13^C_4_-malic acid, 125 nM; U-^13^C amino acid mix (Cambridge Isotope CLM-1548–1), 2.5 μg/mL; ^13^C_5_-glutamine, 6.25 μM;^15^N_2_-asparagine, 1.25 μM;^15^N_2_-tryptophan, 1.25 μM; ^13^C_6_-glucose, 62.5 μM; D_4_-thymine, 1 μM; ^15^N-anthranillic acid, 1 μM; gibberellic acid,1 μM; epibrassinolide, 1 μM. The extraction solvent also contained a 1:400 dilution of Cambridge Isotope carnitine/acylcarnitine mix NSK-B, resulting in the following concentrations of carnitine/acylcarnitine internal standards: D_9_-L-carnitine, 380 nM; D_3_-L-acetylcarnitine, 95 nM; D_3_-L-propionylcarnitine, 19 nM; D_3_-L-butyrylcarnitine, 19 nM; D_9_-L-isovalerylcarnitine, 19 nM; D_3_-L-octanoylcarnitine, 19 nM; D_9_-L-myristoylcarnitine, 19 nM; D_3_-L-palmitoylcarnitine, 38 nM. Sample extraction was performed by adding chilled extraction solvent to tissue sample at a ratio of 1 ml solvent to 50 mg wet tissue mass. Immediately after solvent addition, the sample was homogenized using a Branson 450 probe sonicator. Subsequently, the tubes were mixed several times by inversion and then incubated on ice for 10 minutes. Following incubation, the samples were centrifuged and 300 μL of the supernatant was carefully transferred to two autosampler vials with flat-bottom inserts and dried under a constant stream of nitrogen gas. A QC sample was created by pooling residual supernatants from multiple samples. This QC sample underwent the same drying and reconstitution process as described for individual samples. Dried supernatants were stored at −80 C until ready for instrumental analysis. On the day of analysis, samples were reconstituted in 60 μL of 8:2 v:v water:methanol and then submitted to LC-MS.

Reversed phase LC-MS samples were analyzed on an Agilent 1290 Infinity II / 6545 qTOF MS system with a JetStream electrospray ionization (ESI) source (Agilent Technologies, Santa Clara, California) using a Waters Acquity HSS T3 column, 1.8 μm 2.1 × 100 mm equipped with a matched Vanguard precolumn (Waters Corporation). Mobile phase A was 100% water with 0.1% formic acid and mobile phase B was 100% methanol with 0.025% formic acid. The gradient was as follows: Linear ramp from 0% to 100% B from 0–10 minutes, hold 100% B until 17 minutes, linear return to 0% B from 17 to 17.1 minutes, hold 0% B until 20 minutes. The flow rate was 0.45 ml/min, the column temperature was 55°C, and the injection volume was 5 μL. Each sample was analyzed twice, once in positive and once in negative ion mode MS, scan rate 2 spectra/sec, mass range 50–1200 m/z. Source parameters were: drying gas temperature 350°C, drying gas flow rate 10 L/min, nebulizer pressure 30 psig, sheath gas temperature 350°C and flow 11 L/minute, capillary voltage 3500 V, internal reference mass correction enabled. A QC sample run was performed at minimum every tenth injection.

Ion-pair LC-MS samples were analyzed on an identically-configured LC-MS system using an Agilent Zorbax Extend C18 1.8 μm RRHD column, 2.1 × 150 mm ID, equipped with a matched guard column. Mobile phase A was 97% water, 3% methanol. Mobile phase B was 100% methanol. Both mobile phases contained 15 mM tributylamine and 10 mM acetic acid. Mobile phase C was 100% acetonitrile. Elution was carried out using a linear gradient followed by a multi-step column wash including automated (valve-controlled) backflushing, detailed as follows: 0–2min, 0%B; 2–11 min, linear ramp from 0–99%B; 12–16 min, 99%B, 16–17.5min, 99–0%B. At 17.55 minutes, the 10-port valve was switched to reverse flow (back-flush) through the column. From 17.55–20.45 min the solvent was ramped from 99%B to 99%C. From 20.45–20.95 min the flow rate was ramped up to 0.8 mL/min, which was held until 22.45 min, then ramped down to 0.6mL/min by 22.65 min. From 22.65–23.45 min the solvent was ramped from 99% to 0% C while flow was simultaneously ramped down from 0.6–0.4mL/min. From 23.45 to 29.35 min the flow was ramped from 0.4 to 0.25mL/min; the 10-port valve was returned to restore forward flow through the column at 25 min.. Column temperature was 35°C and the injection volume was 5 μL. MS acquisition was performed in negative ion mode, scan rate 2 spectra/sec, mass range 50–1200 m/z. Source parameters were: drying gas temperature 250°C, drying gas flow rate 13 L/min, nebulizer pressure 35 psig, sheath gas temp 325°C and flow 12 L/min, capillary voltage 3500V, internal reference mass correction enabled. A QC sample run was performed at minimum every tenth injection.

Iterative MS/MS data was acquired for both reverse phase and ion pairing methods using the pooled sample material to enable compound identification. Eight repeated LC-MS/MS runs of the QC sample were performed at three different collision energies (10, 20, and 40) with iterative MS/MS acquisition enabled. The software excluded precursor ions from MS/MS acquisition within 0.5 minute of their MS/MS acquisition time in prior runs, resulting in deeper MS/MS coverage of lower-abundance precursor ions.

Feature detection and alignment was performed utilizing a hybrid targeted/untargeted approach. Targeted compound detection and relative quantitation was performed by automatic integration followed by manual inspection and correction using Profinder v8.0 (Agilent Technologies, Santa Clara, CA.) Non-targeted feature detection was performed using custom scripts that automate operation of the “find by molecular feature” workflow of the Agilent Masshunter Qualitative Analysis (v7) software package. Feature alignment and recursive feature detection were performed using Agilent Mass Profiler Pro (v8.0) and Masshunter Qualitative Analysis (“find by formula” workflow), yielding an aligned table including m/z, RT, and peak areas for all features.

###### Data Cleaning and Degeneracy Removal:

The untargeted features and named metabolites were merged to generate a combined feature table. Features missing from over 50% of all samples in a batch or over 30% of QC samples were then removed prior to downstream normalization procedure. Next, the software package Binner was utilized to remove redundancy and degeneracy in the data.^162^ Briefly, Binner first performs RT-based binning, followed by clustering of features by Pearson’s correlation coefficient, and then assigns annotations for isotopes, adducts or in-source fragments by searching for known mass differences between highly correlated features.

###### Normalization and Quality Control:

Data were then normalized using a Systematic Error Removal Using Random Forest (SERRF) approach,^163^ which helps correct for drift in peak intensity over the batch using data from the QC sample runs. When necessary to correct for residual drift, peak area normalization to closest-matching internal standard was also applied to selected compounds. Both SERRF correction and internal standard normalization were implemented in R. Parameters were set to minimize batch effects and other observable drifts, as visualized using principal component analysis score plots of the full dataset. Normalization performance was also validated by examining relative standard deviation values for additional QC samples not included in the drift correction calculations.

###### Compound identification:

Metabolites from the targeted analysis workflow were identified with high confidence (MSI level 1)^164^ by matching retention time (+/− 0.1 minute), mass (+/− 10 ppm) and isotope profile (peak height and spacing) to authentic standards. MS/MS data corresponding to unidentified features of interest from the untargeted analysis were searched against a spectral library (NIST 2020 MS/MS spectral database or other public spectral databases) to generate putative identifications (MSI level 2) or compound-class level annotations (MSI level 3) as described previously.^165^

##### LC-MS/MS untargeted lipidomics

###### Sample preparation:

Non-targeted lipid analysis was conducted at the Georgia Institute of Technology. Powdered tissue samples (10 mg) were extracted in 400 μL isopropanol containing stable isotope-labeled internal standards (IS) with bead homogenization with 2mm zirconium oxide beads (Next Advance) using a TissueLyser II (10min, 30 Hz). Samples were then centrifuged (5 min, 21,100xg), and supernatants were transferred to autosampler vials. Plasma samples (25 μL) were extracted by mixing with 75 μL isopropanol containing the IS mix followed by centrifugation. Sample blanks, pooled extract samples used as quality controls (QC), and consortium reference samples, were prepared for analysis using the same methods. The IS mix consisted of PC (15:0–18:1(d7)), Catalog No. 791637; PE (15:0–18:1(d7)), Catalog No. 791638; PS (15:0–18:1(d7)), Catalog No. 791639; PG(15:0–18:1(d7)), Catalog No. 791640; PI(15:0–18:1(d7)), Catalog No. 791641; LPC(18:1(d7)), Catalog No. 791643; LPE(18:1(d7)); Catalog No. 791644; Chol Ester (18:1(d7)), Catalog No. 700185; DG(15:0–18:1(d7)), Catalog No. 791647; TG(15:0–18:1(d7)-15:0), Catalog No. 791648; SM(18:1(d9)), Catalog No. 791649; Cholesterol (d7), Catalog No. 700041. All internal standards were purchased from Avanti Polar Lipids (Alabaster, Alabama) and added to the extraction solvent at a final concentration in the 0.1–8 μg/ml range.

###### Data collection:

Lipid LC-MS data were acquired using a Vanquish (ThermoFisher Scientific) chromatograph fitted with a ThermoFisher Scientific AccucoreTM C30 column (2.1 × 150 mm, 2.6 μm particle size), coupled to a high-resolution accurate mass Q-Exactive HF Orbitrap mass spectrometer (ThermoFisher Scientific) for both positive and negative ionization modes. For positive mode analysis, the mobile phases were 40:60 water:acetonitrile with 10 mM ammonium formate and 0.1% formic acid (mobile phase A), and 10:90 acetonitrile:isopropyl alcohol, with 10 mM ammonium formate and 0.1% formic acid (mobile phase B). For negative mode analysis, the mobile phases were 40:60 water:acetonitrile with 10 mM ammonium acetate (mobile phase A), and 10:90 acetonitrile:isopropyl alcohol, with 10 mM ammonium acetate (mobile phase B). The chromatographic method used for both ionization modes was the following gradient program: 0 minutes 80% A; 1 minute 40% A; 5 minutes 30% A; 5.5 minutes 15% A; 8 minutes 10% A; held 8.2 minutes to 10.5 minutes 0% A; 10.7 minutes 80% A; and held until 12 minutes. The flow rate was set at 0.40 ml/min. The column temperature was set to 50°C, and the injection volume was 2 μL.

For analysis of the organic phase the electrospray ionization source was operated at a vaporizer temperature of 425°C, a spray voltage of 3.0 kV for positive ionization mode and 2.8 kV for negative ionization mode, sheath, auxiliary, and sweep gas flows of 60, 18, and 4 (arbitrary units), respectively, and capillary temperature of 275°C. The instrument acquired full MS data with 240,000 resolution over the 150–2000 m/z range. LC-MS/MS experiments were acquired using a DDA strategy. MS2 spectra were collected with a resolution of 120,000 and the dd-MS2 were collected at a resolution of 30,000 and an isolation window of 0.4 m/z with a loop count of top 7. Stepped normalized collision energies of 10%, 30%, and 50% fragmented selected precursors in the collision cell. Dynamic exclusion was set at 7 seconds and ions with charges greater than 2 were omitted.

###### Data processing:

Data processing steps included peak detection, spectral alignment, grouping of isotopic peaks and adduct ions, drift correction, and gap filling. Compound Discoverer V3.3 (ThermoFisher Scientific) was used to process the raw LC-MS data. Drift correction was performed on each individual feature, where a Systematic Error Removal using Random Forest (SERRF) method builds a model using the pooled QC sample peak areas across the batch and was then used to correct the peak area for that specific feature in the samples. Detected features were filtered with background and QC filters. Features with abundance lower than 5x the background signal in the sample blanks and that were not present in at least 50% of the QC pooled injections with a coefficient of variance (CV) lower than 80% (not drift corrected) and 50% (drift corrected) were removed from the dataset. Lipid annotations were accomplished based on accurate mass and relative isotopic abundances (to assign elemental formula), retention time (to assign lipid class), and MS2 fragmentation pattern matching to local spectral databases built from curated experimental data. Lipid nomenclature followed that described previously.^166^

###### Quality control procedures:

System suitability was assessed prior to the analysis of each batch. A performance baseline for a clean instrument was established before any experiments were conducted. The mass spectrometers were mass calibrated, mass accuracy and mass resolution were checked to be within manufacturer specifications, and signal-to-noise ratios for the suite of IS checked to be at least 75% of the clean baseline values. For LC-MS assays, an IS mix consisting of 12 standards was injected to establish baseline separation parameters for each new column. The performance of the LC gradient was assessed by inspection of the column back pressure trace, which had to be stable within an acceptable range (less than 30% change). Each IS mix component was visually evaluated for chromatographic peak shape, retention time (lower than 0.2 minute drift from baseline values) and FWHM lower than 125% of the baseline measurements. The CV of the average signal intensity and CV of the IS (<=15%) in pooled samples were also checked. These pooled QC samples were used to correct for instrument sensitivity drift over the various batches using a procedure similar to that described by the Human Serum Metabolome (HUSERMET) Consortium.(Dunn et al. 2011) To evaluate the quality of the data for the samples themselves, the IS signals across the batch were monitored, PCA modeling for all samples and features before and after drift correction was conducted, and Pearson correlations calculated between each sample and the median of the QC samples.

##### Metabolomics/Lipidomics Data filtering and normalization

The untargeted metabolomics datasets were categorized as either “named”, for chemical compounds confidently identified, or “unnamed”, for compounds with specific chemical properties but without a standard chemical name. While the preprocessing steps were performed on the named and unnamed portions together, only the named portions were utilized for differential analysis. For each dataset i.e. each assay for each tissue type, the following steps are performed:

- Average rows that have the same metabolite ID.
- Merge the “named” and “unnamed” subparts of the untargeted datasets.
- Convert negative and zero values to NAs.
- Remove features with > 20% missing values.
- Features with < 20% missing values are imputed either using K-Nearest Neighbor imputation (for datasets with > 12 features) or half-minimum imputation (for datasets with < 12 features).
- All data are log2-transformed, and the untargeted data are median-MAD normalized if neither sample medians nor upper quartiles were significantly associated with sex or sex-stratified training group (Kruskal-Wallis p-value < 0.01). Note that for all log2 calculations, 1 is added to each value before log-normalization. This allows metabolite values that fall between 0 and 1 to have a positive log2 value.

Outlier detection was performed by examining the boxplot of each Principal Component and extending its whiskers to the predefined multiplier above and below the interquartile range (5x the IQR). Samples outside this range are flagged. All outliers were reviewed by Metabolomics CAS, and only confirmed technical outliers were removed.

##### Redundant Metabolite/Lipid Management

To address metabolites measured on multiple platforms (e.g. a metabolite measured on the HILIC positive and RP positive platforms), and metabolites with the same corresponding RefMet ID (e.g. alpha-Aminoadipic-acid, Aminoadipic acid both correspond to RefMet name ‘Aminoadipic acid’), we utilize the set of internal standards described above to make a decision on which platform’s measurement of a given feature to include in further analysis. For each tissue, based on whichever platform has the lowest coefficient of variation across all included reference standards for a given refmet id, that metabolite was chosen. The other platforms or metabolites, for this tissue, corresponding to this refmet id were removed from further downstream analysis. Data for all metabolites removed, including information about the coefficient of variation in the standards, normalized data, or differential analysis results, is available in the R Package(see below), but is not loaded by default.

### Statistical analysis

#### Differential analysis

To model the effects of both exercise modality and time, relative to non exercising control, each measured molecular feature was treated as an outcome in a linear mixed effects model accounting for fixed effects of exercise group (RE, EE, or CON), timepoint, as well as demographic and technical covariates (see covariate selection below for more info). Participant identification was treated as a random effect. For each molecular feature, a cell-means model is fit to estimate the mean of each exercise group-timepoint combination, and all hypothesis tests are done comparing the means of the fixed effect group-timepoint combinations. In order to model the effects of exercise against non-exercising control, a difference-in-changes model was used which compared the change from pre-exercise to a during or post-exercise timepoint in one of the two exercise groups to the same change in control. This effect is sometimes referred to as a “delta-delta” or “difference-in-differences” model.

#### Model selection via simulation

In order to determine the optimal statistical package/model for these data – given MoTrPAC’s unique sampling design (see ‘acute exercise intervention’ above) – a simulation study was conducted to determine the impact of various approaches to account for correlation in repeated measurements. The simulation evaluated type 1 error, power, and bias for multiple plausible modeling approaches, to identify those that would obtain higher power while maintaining nominal type 1 error rates. For a subset of molecular features in the datasets, mean, covariance, skew, and kurtosis were summarized over the relevant timepoints in the acute exercise bout within subgroups defined by sex and exercise type. Then, these summary values were used in combination with the “covsim” R package to generate non-normally distributed simulated data.^167^ Additionally, sample size and missingness patterns were aligned in the simulated data to match those of the observed data. Using a total of 5 million simulated instances where data were generated under the null hypothesis (i.e., no change in the mean values of the outcome over the exercise timepoints) or alternative hypothesis (i.e., the mean value of the outcome differs between at least two timepoints), type 1 error rate and power, respectively, were evaluated for seven analytic strategies: (1) pairwise t-tests between timepoints of interest, (2) ordinary least squares regression, (3) differential expression for repeated measures (i.e., dream) with random intercepts,^129^ (4) linear mixed models with random intercepts, (5) mixed models for repeated measures using an unstructured covariance matrix, and (6) generalized linear models using generalized estimating equations (GEEs) and a first degree autoregressive correlation structure. These analyses were replicated for adipose, blood, and muscle samples. The dream function obtained type 1 error rates 5.2, 5.4, and 5.2% for adipose, blood, and muscle analyses, respectively, with higher power than all other methods apart from generalized linear models with GEEs. Although generalized linear models with GEEs had higher power than dream, they also had higher type 1 error with 7.7%, 6.0%, and 6.5% type 1 error rates for adipose, blood, and muscle analyses, respectively. Therefore, due to stability of type 1 error and relatively high power compared to other approaches, the *dream* function from the variancePartition R package was selected for the primary analyses for every omic platform except Methyl-Cap (see methylation capture sequencing methods above for more details).^129^

#### Covariate selection

Covariates were selected through a combination of a priori knowledge about factors influencing molecular levels as well through empiric screening. Factors were considered for model inclusion by correlating the principal components of each tissue-ome feature set to demographic and technical factors using variancePartition::canCorPairs.^129^ Visualizations and computational analysis that describe this process for the decisions for covariate selection can be found at: https://github.com/MoTrPAC/MotrpacPreSuspensionAcute/tree/main/QC. Ultimately, the following fixed effect covariates were included in the models for every omic platform, except Methyl Cap (see methods above): group and timepoint in a cell means model, clinical site, age, sex, and BMI. ‘Participant id’ was included as a random effect in every model. Each ome then had ome-specific covariates selected as described in prior sections.

A targeted analysis was conducted to assess whether and how race, ethnicity, and genetic ancestry could be incorporated, given prior evidence that these factors can influence omic data.^168,169^ We evaluated multiple models that included patient-reported race and/or ethnicity, as well as principal components derived from whole-genome sequencing (WGS), to determine their impact on model fit using the BIC. However, no combinations of individual or joint inclusion of race, ethnicity, or WGS-derived principal components led to an improvement in average model BIC across all features for a platform.

The final set of covariates included in each model for each tissue and ome can be found in the R Package *MotrpacHumanPreSuspensionAnalysis*, and in Supplementary Table 2.

#### Missingness

Data missingness varied for multiple reasons including experimental design (i.e. temporal randomization which randomized some participants to have samples obtained at a subset of timepoints, see Table S1 and assay limitations (metabolomics and proteomics can have missingness as described in their individual sections). For some omic sets, imputation to alleviate missingness could be performed (metabolomics) but for others was not, including proteomics. Analysis demonstrated proteomics missingness was approximately at random (data not shown).

Given the above issues affecting the ultimate sample size for effect estimation, for a feature to be included in the analysis, a minimum number of 3 participants were required to have a paired pre-exercise sample for all groups and all during/post-exercise timepoints. Thus, all group comparisons (e.g. RE vs CON at 3.5 hours post-exercise) required 3 participants with matched pre- and post-exercise samples in each group. This requirement was satisfied in all omes and tissues except a subset of MS-acquired proteomics and phosphoproteomics features in SKM and AT. Features not meeting this criterion in any group-timepoint set were excluded from differential analysis entirely, though raw and QC-normalized values are available.

#### Specific omic-level statistical considerations

Models for all omes other than methylation were fit using ‘variancePartition::dream’.^129^ For the transcriptomics and ATAC-seq, which are measured as numbers of counts, the mean-variance relationship was measured using ‘variancePartition::voomWithDreamWeights’ as previously described.^129^ For all other omes the normalized values were used directly as input to the statistical model.

The full implementation of the statistical models for each of non-methylation datasets can be found in the R Package *MotrpacHumanPreSuspensionAnalysis*. Methylation data was processed separately, and the methods can be found in the methylation methods section.

#### Contrast types

The specific contrasts made in the statistical analysis include three categories of comparison: difference-in-changes relative to control, group-specific, and resistance vs endurance. All comparisons were structured using ‘variancePartition::makeContrastsDream’.^129^

The difference-in-changes model as described at the opening of this section represents the primary DA analysis, and any individual feature mentioned as statistically significantly changing due to exercise will be referring to the difference-in-changes results unless otherwise specifically stated.

The next category of contrasts is a group-specific comparison, which compares a given post-intervention timepoint to pre-exercise within a given group, without comparing to the control group. This contrast was implemented to more easily quantify effect sizes within each group independently, but are not used as a general endpoint.

Finally, the RE vs EE is very similar to the difference-in-changes model, but sets the RE group as a matched control to the EE group. In this comparison, the change in response to exercise at a given timepoint in one exercise group is directly compared to the corresponding change in the other exercise group. This contrast can directly test the hypothesis that the EE effect is different from the RE effect. There are no samples obtained during RE bouts, so those timepoints in blood were excluded from this comparison.

#### Significance thresholds

For each of the above contrasts, p-values were adjusted for multiple comparisons for each unique contrast-group-tissue-ome-timepoint combination separately using the Benjamini-Hochberg method to control False Discovery Rate (FDR).^170^ Features were considered significant at a FDR of 0.05 unless otherwise stated.

#### Human feature to gene mapping

The feature-to-gene map links each feature tested in differential analysis to a gene, using Ensembl version 105 (mapped to GENCODE 39)^171^ as the gene identifier source. Proteomics feature IDs (UniProt IDs) were mapped to gene symbols and Entrez IDs using UniProt’s mapping files.^172^ Epigenomics features were mapped to the nearest gene using the ChIPseeker::annotatePeak()^173,174^ function with Homo sapiens Ensembl release 105 gene annotations. Gene symbols, Entrez IDs, and Ensembl IDs were assigned to features using biomaRt version 2.58.2 (Bioconductor 3.18).^175–177^

For ATACseq and methyl capture features, relationship to gene and custom annotation provide gene proximity information and custom ChIPseeker annotations, respectively. Relationship to gene values indicate the distance of the feature from the closest gene, with 0 indicating overlap. Custom annotation categories include Distal Intergenic, Promoter (<=1kb), Exon, Promoter (1–2kb), Downstream (<5kb), Upstream (<5kb), 5' UTR, Intron, 3' UTR, and Overlaps Gene.

Metabolite features were mapped to KEGG IDs using KEGGREST, RefMet REST API, or web scraping from the Metabolomics Workbench.^178,179^

### Enrichment analysis

#### Preparation of differential analysis results

The differential analysis results tables for each combination of tissue and ome were converted to matrices of z-scores with either gene symbols, RefMet metabolite/lipid IDs, or phosphorylation flanking sequences as rows and contrasts as columns. These matrices serve as input for the enrichment analyses. For the transcriptomics, TMT proteomics, and Olink proteomics results, transcripts and proteins were first mapped to gene symbols. To resolve cases where multiple features mapped to a single gene, only the most extreme z-score for each combination of tissue, contrast, and gene was retained. Metabolite and lipid identifiers were standardized using the Metabolomic Workbench Reference List of Metabolite Names (RefMet) database.^180^ For the CAMERA-PR enrichment of phosphoproteomics results, since peptides could be phosphorylated at multiple positions, phosphorylation sites were separated into single sites with identical row information (e.g., protein_S1;S2 becomes protein_S1 and protein_S2, both with the same data). For any combinations of contrast and phosphosite that were not uniquely defined, only the most extreme z-score (maximum absolute value) was selected for inclusion in the matrix. Singly phosphorylated sites were required to use the curated kinase–substrate relationship information available from PhosphoSitePlus (PSP),^181^ described below.

#### Gene set selection

Gene sets were obtained from the MitoCarta3.0 database, CellMarker 2.0 database,^182^ and the C2-CP (excluding KEGG_LEGACY) and C5 collections of the human Molecular Signatures Database (MSigDB; v2023.2.Hs)^182–184^ Metabolites and lipids were grouped according to chemical subclasses from the RefMet database.^180^ This includes subclasses such as “Acyl carnitines” and “Saturated fatty acids”.Flanking sequences for protein phosphorylation sites were grouped according to their known human protein kinases provided in the *Kinase_Substrate_Dataset* file from PhosphoSitePlus (PSP; v6.7.1.1; https://www.phosphosite.org/staticDownloads.action; last modified 2023-11-17).^181^ In addition to these kinase sets, sets from the directional Post-Translational Modification Signatures Database (PTMsigDB) were included for analysis.^40^

For each combination of tissue and ome, molecular signatures were filtered to only those genes, metabolites/lipids, or flanking sequences that appeared in the differential analysis results. After filtering, all molecular signatures were required to contain at least 5 features, with no restriction on the maximum size of sets. Additionally, gene sets were only kept if they retained at least 70% of their original genes to increase the likelihood that the genes that remain in a given set are accurately described by the set label.

#### Analysis of Molecular Signatures

Analysis of molecular signatures was carried out with the pre-ranked Correlation Adjusted MEan RAnk (CAMERA-PR) gene set test using the z-score matrices described in the “Preparation of differential analysis results” section to summarize the differential analysis results for each contrast at the level of molecular signatures.^40,185^ Z-scores were selected as the input statistics primarily to satisfy the normality assumption of CAMERA-PR, and the analysis was carried out with the *cameraPR.matrix* function from the TMSig R/Bioconductor package.^186,187^

#### Over-representation analysis (ORA)

Over-representation analysis (ORA) was conducted using the run_ORA function from the *MotrpacHumanPreSuspensionAnalysis* R package (v0.0.1.53) which uses the hypergeometric test for statistical significance. The input consisted of differentially expressed genes (adjusted p-value < 0.05) identified from one or more omic layers in a particular tissue. The background gene set included all genes detected in each of the ome(s) and tissue(s) under consideration.

#### Statistical significance thresholds

For both CAMERA-PR and ORA results, p-values were adjusted within each combination of tissue, ome, contrast, and broad molecular signature collection (MitoCarta3.0, CellMarker 2.0, C2, C5, RefMet, PSP, and PTMsigDB) using the Bejamini-Hochberg method. Gene sets and RefMet subclasses were declared significant if their adjusted p-values were less than 0.05. This threshold was raised to 0.1 for kinase sets.

#### Visualization methods

Bubble heatmaps were generated from the enrichment analysis results using the *enrichmap* function from the TMSig R/Bioconductor package.^186^

### Clustering techniques

#### Fuzzy C-Means Clustering

The z-score matrices described in the Enrichment Analysis Methods were used as input for fuzzy c-means (FCM) clustering.^188,189^ For each tissue, the matrices for the different omes, excluding global proteomics and phosphoproteomics in adipose (as these were only measured at two timepoints) were stacked to form a single matrix with tissue-specific timepoints as columns and features being a row. For blood, the “during exercise” contrasts were also excluded. The rows of this matrix were then divided by their sample standard deviations, which were calculated after two columns of zeros were temporarily included to represent the pre-exercise timepoints; these zero columns were discarded before clustering. The Mfuzz *R* package was used to perform FCM clustering with the parameter *m* set to the value of *Mfuzz::mestimate* for each tissue.^190,191^ The minimum distance between centroids was used as the cluster validity index to determine the optimal number of clusters for each tissue. A scree/elbow plot of these distances for a range of cluster numbers (3–14) was generated for each tissue, and the final cluster numbers were determined through manual inspection: 13 for both adipose and blood, and 12 for muscle. For visualization purposes, hard clustering was performed by assigning each feature to the cluster with the highest membership probability; features with probabilities below 0.3 are not clustered or displayed. Cluster numbers are assigned arbitrarily.

#### Analysis of clusters

In order to uncover the biological relevance of each cluster, non-parametric CAMERA-PR as well as ORA was applied to the matrix of cluster membership probabilities separately for each tissue and ome, though CAMERA-PR results are available only though the R package.^185^ The same molecular signatures described in the “Enrichment Analysis” Methods were tested. The non-parametric version of CAMERA-PR is a modification of the two-sample Wilcoxon–Mann–Whitney rank sum test. As with the parametric version, it accounts for inter-molecular correlation to control the false positive rate. Upper-tailed tests were used to determine which molecular signatures were highly ranked according to their membership probabilities (i.e., which molecular signatures closely followed the cluster centroids).

#### PLIER methods

To delve into cross-tissue temporal trends in acute exercise response, we applied PLIER,^58^ which takes an expression matrix of features vs samples as input and outputs patterns of activity, in the form of latent variables (LVs), and connects these patterns with user-provided prior information, such as reactome or KEGG pathways, or structural classifications in the case of metabolomics data. We use the PLIER package R function *num.pc* to identify the number of significant principle components for the singular value decomposition (SVD) of the data and specify double this number as the desired LV count. If *num.pc* is computationally infeasible or produces a value > 50, we use a default of 100 LVs for a PLIER run, with rigorous testing of alternative LV depth to ensure coverage of the significant discoverable exercise patterns. Both the cross-tissue RNA analysis and the cross-tissue metabolomics analysis specified the default 100 LVs. PLIER works to identify a set of LVs that best account for all patterns identified in the dataset, optimizing individual LV trajectories over the samples and feature associations to minimize the difference between normalized input data and PLIER LV output, while also accounting for feature associations with prior knowledge in the form of pathway enrichments or metabolite classifications.

We ran PLIER separately on the cross-tissue RNA seq data and on the cross-tissue metabolomics data. For each ome separately studied, we generated a cross-tissue matrix of exercise response, selecting the subset of features that were present in all three tissues, then z-scoring the normalized data matrix for the given ome for each tissue. Following by-tissue z-scoring, the z-scored tissue matrices are concatenated into one large matrix with rows corresponding to the consensus features across all tissues, and columns the cumulative samples from each tissue. Significance of an LV to differentiate resistance or endurance exercise from control at a given timepoint relative to pre-exercise is determined by comparing the differences in LV value post-exercise vs pre-exercise for each subject at that timepoint separately between resistance and control and endurance and control within each tissue and adjusting for multiple hypotheses using the *compare_means* R function.^192^

#### Transcription Factor Motif Enrichment

Transcription factor motif enrichment analysis was performed on sets of DEGs for each tissue, timepoint and exercise modality combination. DEGs for motif enrichment analysis were selected for each tissue by satisfying an unadjusted p-value threshold of 0.05. Motif enrichment analysis was carried out by findMotifs.pl (HOMER v4.11.1)^73^ on a set of 472 TF motifs provided by the HOMER package. Enrichment was performed on a subset of the proximal promoter region of each DEG, ranging from 300 bp upstream to 50 bp downstream of the transcription start site (TSS), and normalized for GC%. All HOMER runs used the same background set of all gene promoters. The search lengths of the motifs were 8, 10, and 12 bp. Downstream analysis of HOMER results were conducted using the knownResults.txt output file of enrichments of the 472 inputted TF motifs using Benjamini-Hochberg FDR-corrected p-values for significance of TF enrichment.

#### Spatiotemporal Clustering and Inference of Omics Networks

Spatiotemporal Clustering and Inference of Omics Networks (SC-ION, https://github.com/nmclark2/SCION)^78^ was applied to the dataset to infer unsupervised multi-omics networks of exercise response. The source code for SC-ION was adapted for the *MotrpacHumanPreSuspensionAnalysis* R package such that the SC-ION analysis can be repeated or repurposed for additional data within this study (see Package assembly and distribution). The exact parameters used to generate results for the figures presented in the manuscript are found in the figures folder in the MotrpacPreSuspensionAcute repository.

#### Selecting regulator and target data

SC-ION takes as input two data matrices where one matrix is assigned as the “regulator” matrix and the other is assigned as the “target” matrix. Two different schemes for regulator/target matrices were considered. In the first scheme (Muscle-only network), phosphosites on transcription factors (TFs) in muscle were used as the regulators, and DA transcripts in the muscle were used as the targets. In the second scheme (Blood-to-muscle network), metabolites in blood were considered regulators, and DA transcripts in muscle were again the targets. In both schemes, EE and RE samples were separated, and one network was inferred for each modality. Control samples were excluded from the SC-ION analysis. The final output is therefore four separate networks: Muscle-only EE, Muscle-only RE, Blood-to-muscle EE, and Blood-to-muscle RE.

#### Preparation of input data

For the muscle-only network, the normalized values for the muscle phosphoproteome were used as the regulator matrix. Since SC-ION cannot incorporate missing values, the muscle phosphoproteome was filtered for phosphosites with no more than 40% missing values, and then missing values were imputed with Multiple Imputation by Chained Equations (MICE)(DOI:10.18637/jss.v045.i03) by using the average imputed value from 15 multiple imputations. This imputed matrix was filtered for phosphosites located on TFs using “The Human Transcription Factors” database (https://humantfs.ccbr.utoronto.ca/). For the blood-to-muscle network, the normalized values for the blood metabolites were used as the regulator matrix. For both networks, the normalized values for the muscle transcriptome were used as the target matrix. The target matrix was filtered for DA transcripts (adjusted p-value < 0.05 in any exercise-with-control contrast). All matrices were split into EE and RE samples prior to running SC-ION.

#### Clustering

SC-ION incorporates an optional clustering step to group together features with similar expression profiles prior to inferring the network. For this implementation, c-means clustering (see C-means clustering) was used to cluster only the regulator and target features used as inputs to each network.

#### Edge trimming

All of the edges in the inferred networks are assigned a weight which indicates the relative confidence in the prediction. To trim the networks based on edge weights, a permutation analysis was performed by randomly shuffling the regulator and target matrices and running SC-ION 100 times. The edge weights were then ranked and assigned a *p*-value based on a permutation test. The *p*-values were then adjusted using Benjamini-Hochberg (BH). The networks were originally trimmed to edge weights with a BH-adjusted *p*-value < 0.05 as determined by the permutation test. However, this resulted in overly dense networks, so a more conservative edge weight cutoff (edge weight > 0.1) was chosen based on the edge weight distribution.

#### Importance score calculation

Following network inference, all edges from the two EE networks were merged using a union (keeping all edges) to form a combined EE network (same for the combined RE network). A Network Motif Score (NMS) was calculated for each feature in the combined EE and combined RE network.^78^ Feed-forward loop (FFL), diamond, and 3-chain motifs were used as input for the NMS (Figure 7A). The total number of motifs, as well as the features within each motif, were determined using the NetMatchStar application in Cytoscape.^193^ This resulted in two NMS, one for EE and one for RE. Further, an average NMS was calculated to determine the overall importance of each feature in the combined EE+RE network.

#### Visualization

The combined EE and RE networks were merged using a union to form the final, combined EE+RE network, which is reported along with the NMS in Table S7. All network visualization on the combined EE+RE network was performed in Cytoscape.

#### Secreted factor analysis

Differential abundance results were aligned according to relative timepoint and tissue. First, DA features from RNAseq, MS proteomics, and phosphoproteomics were identified after filtering for features that had an FDR adjusted p-value of < 0.1. No limitations were placed on direction of effect. DA features were mapped to their respective gene symbol using the human feature-to-gene map as described above. This gene symbol was then used to connect DA features in muscle, adipose, or blood cells to the circulating plasma proteins as measured by Olink. If DA features in the tissues matched DA protein features in the plasma. These features were then annotated with their extracellular likelihood using subcellular localization database (“COMPARTMENTS”).^104^ COMPARTMENTS provides location scores ranging from 0 to 5, with higher scores indicating greater confidence in localization. The “All channels integrated” file for human proteins was obtained from (https://compartments.jensenlab.org/Search). To calculate the extracellular score, we selected the highest score among the following compartments associated with extracellular localization: *Extracellular region*, *Extracellular space*, *Extracellular exosome*, and *Extracellular vesicle*.

For CCN1 intra- and inter-tissue correlation (i.e,. Muscle and adipose), biweight midcorrelation was conducted. For gene set enrichment analysis on CCN1-associated transcripts in muscle and adipose, CAMERA-PR was conducted on tissue transcripts correlating with its CCN1 expression, using correlation coefficient (i.e., bicor) as the metric.

### QUANTIFICATION AND STATISTICAL ANALYSIS

#### Statistical parameter reporting

All relevant statistical parameters for the analyses performed in this study such as sample size (*N*), center and spread (e.g. mean/median, standard deviation/error), statistical methodology (e.g. mixed-effects linear model) and significance cutoffs (e.g. adjusted *p*-value < 0.05) are reported in the main text, figure legends, STAR Methods, and/or supplementary information. Where appropriate, methods used to determine the validity of certain statistical assumptions are discussed.

#### Statistical limitations

This report presents analyses and results for 206 participants randomized before the suspension of the MoTrPAC study due to the COVID-19 pandemic. The main post-suspension MoTrPAC study will include over 1,500 participants randomized under a slightly modified protocol.^34^ The current paper focuses on evaluating feasibility and generating hypotheses for the main study. Results should be interpreted with caution for several reasons: 1) small sample sizes reduce the power to detect even moderate effects;^194^ 2) simple randomization of small groups can create imbalances in both known and unknown confounders;^195,196^ and 3) NIH initiatives have long emphasized caution in interpreting small studies to enhance reproducibility.^197^

Although blocked randomization with site-based stratification was employed, discrepancies in regulatory approvals, start-up times, and pandemic-related interruptions resulted in imbalanced and small sample sizes. Specific limitations in the baseline (pre-intervention) data include: 1) subgroup sample sizes based on intervention group, sex, and timepoint as small as 4 participants; 2) limited generalizability, as 50% of controls with biosamples were randomized at two of the ten sites, and 74% of participants at four sites; and 3) “sex differences” may reflect “body composition differences” due to insufficient data to disentangle sex from body composition, and variations in DXA machine operators and brands across sites.

Testing for heterogeneity of response in small subgroups was largely avoided. As Brookes et al. show,^198^ interaction effects must be at least double the main effect to achieve 80% power. While the EE and RE groups each had around 70 participants with biosamples, providing 80% power to detect a 0.5 effect size (difference in means/SD) using a two-sample t-test (alpha = 0.05, two-sided), for tests of interaction effects to have 80% power an interaction effect size > 1 would be required, which is considered large.^199^ Consequently, heterogeneity of response was explored only descriptively within the larger randomized groups (EE/RE), with some inferential statistics (e.g., confidence intervals, p-values) emphasizing interval estimation. Our aim was to present the results from a hypothesis-generating perspective, following Ioannidis's cautionary guidance,^194^ and to lay a solid foundation for future analyses in the main MoTrPAC study.

#### Additional resources

##### Package assembly and distribution

The *MotrpacHumanPreSuspensionAnalysis* R package (https://github.com/MoTrPAC/MotrpacHumanPreSuspensionAnalysis) contains functions to generate visualizations, as well as data objects for differential abundance analysis, summary statistics for normalized expression data, feature to gene mapping, and molecular signature datasets as described in the methods.

The MotrpacHumanPreSuspensionData R package (https://github.com/MoTrPAC/MotrpacHumanPreSuspensionData) is a package containing individual level data, which cannot be made openly available and can be accessed with approval from a data access committee. See motrpac-data.org for details.

Data used in the preparation of this article were obtained from the Molecular Transducers of Physical Activity Consortium (MoTrPAC) database, which is available for public access at motrpac-data.org. The specific version released is version 1.3 of the human precovid sed adu.

Lastly, the MotrpacPreSuspensionAcute GitHub repository (https://github.com/MoTrPAC/MotrpacPreSuspensionAcute) contains code and individual parameters for each figure panel, utilizing the MotrpacHumanPreSuspensionAnalysis and MotrpacHumanPreSuspensionData packages.

## Supplementary Material

Supplement 1 Table S1. Participant numbers, related to Figure 1

Supplement 2 Table S2. Detailed differential analysis results, related to Figure 2

Supplement 3 Table S3. Overrepresentation analysis results from c-means clusters, related to Figure 3

Supplement 4 Table S4. Cross tissue RNA and Metabolomics PLIER results, related to Figure 4

Supplement 5 Table S5. Direct comparison of EE vs. RE, related to Figure 5

Supplement 6 Table S6. Homer analysis results, related to Figure 6

Supplement 7 Table S7. SC-ION results, related to Figure 7

Supplement 8 Table S8. Exerkine candidate list, related to Figure 8

1 **Figure S1. Participant genotypes, sample-ome overlap distribution and tissue-ome covariate structure, related to** Figure 1 A. First two principal components of genetic ancestry. Participants from MoTrPAC are plotted using triangles, while participants from 1000 Genomes are plotted using points. Categorical ancestry according to 1000 Genomes is indicated by color. MoTrPAC participants are not categorized by ancestry.
B. Matrix of omic data availability. Each row of the heatmap represents a participant, with each participant represented once in each of the three tissues. If data is available for a particular participant for a given tissue-ome-time point combination, this is indicated by a colored cell in the matrix. A grey cell indicates no omic data available for the sample collected. A white cell indicates that no sample was collected in that participant for that tissue-time point.
C. Canonical correlation analysis of the first five principal components of ome-tissue combinations to key MoTrPAC experimental covariates. Colors in each block represent covariance between each principal component and each experimental parameter. Metabolite platforms were combined into one matrix and subset to participant-timepoint combinations present in all metabolomic platforms. Principal component labels indicate percentage of total omic variance explained by each principal component.
D. Percentages of variance explained by experimental design parameters for each feature in every available ome-tissue combination. After fitting a linear mixed model for each feature with complete data, the percentage of variance explained per covariate per feature is represented on the y axis. Residual variance is not visualized in this plot. A grey cell (adipose ATAC, blood phosphoproteomics) indicates no omic data where a sample was collected.
**Figure S2. Distribution and overlap of differential multi-omic features across tissues, related to** Figure 2 A. Barplot summarizing the classification of molecular features by type of differential response across tissues and omes. Features were grouped into three categories based on significance patterns in exercise versus control comparisons: (1) Control-driven exercise effects; (2) Exercise effects; and (3) Non-exercise induced changes. This categorization is intended to distinguish biologically meaningful exercise responses from potential false positives from otherwise uncontrolled experimental designs. Each feature upregulated relative to control is plotted above the axis, and each downregulated relative to control is plotted below the axis.
B. Representative examples of molecular features classified into categories based on their differential response patterns across timepoints and conditions. Alloisoleucine (Adipose metabolomics) illustrates non-exercise induced changes. *PPARGC1A* (Muscle RNA-seq) represents a robust exercise effect. HSPA4 (Muscle Phosphoproteomics) and *PER2* (Muscle RNA-seq) exemplifies a control-driven effect. Pre = pre-exercise, P15–45M = 15, 30, 45 minutes post exercise (depending on tissue), P3.5–4H = 3.5 or 4 hours post exercise (depending on tissue, P24H = 24 hours post exercise. Data is shown as group-time point mean ± 95% confidence interval with a black circle indicating significance relative to control at adj. p-val<0.05. Plots indicate Ensembl for RNA or Uniprot IDs for proteins where appropriate.
C. UpSet plots by ome across tissues: ATAC-seq, MethylCap-seq, transcriptomics, proteomics, phosphoproteomics, and metabolomics. Each plot displays the overlap of significant features (adjusted p-value < 0.05) across three tissues (AT, SKM, and blood) regardless of exercise modality or timepoint. Vertical bars represent the number of features unique to or shared among the tissues, as shown in the matrix below each plot. Numbers on top of the bars indicate the total number of features in that set.
D. Over-representation analysis of DA phosphosites unique or shared between the AT and SKM using PTMsigDB.
E. Temporal trajectories of select DA features shared between all three tissues. Pre = pre-exercise, D20M = during 20 minutes, D40M = during 40 minutes, P10M = 10 minutes post exercise, P15–45M = 15, 30, 45 minutes post exercise (depending on tissue), P3.5–4H = 3.5 or 4 hours post exercise (depending on tissue, P24H = 24 hours post exercise. Points represent group means ± standard error; black points circles denote comparisons to control with adjusted p-values < 0.05. Plots indicate Ensembl for RNA.
F. Panel of three UpSet plots, one for each tissue (AT, SKM, and blood), illustrating the intersection of all significant features (adjusted p-value < 0.05) across epigenome (ATAC-seq, MethyCap-seq), transcriptome, proteome, and phosphoproteome (metabolomics data was not included). Features were mapped to gene symbols to enable direct comparison across omics layers. For each tissue, vertical bars represent the number of features unique to, or shared among, the indicated omes as shown by the matrix below each plot. Features that were DA at any time point and in either modality were considered for matching across omic layers.
**Figure S3. Multi-tissue metabolomic enrichments and gene heatmaps of significant C means trajectory pathways, related to** Figure 3 A. Pathway Enrichment for each tissue-timepoint in metabolomic RefMet classes via Pre-ranked Correlation-Adjusted MEan-RAnk (CAMERA-PR).
B.-D. Multi-tissue heatmaps of Z-scores for transcriptomic features belonging to the GOBP: PROCESS UTILIZING AUTOPHAGIC MECHANISM. Columns represent time points grouped by tissue and exercise modality. The right-hand annotation indicates the c-means cluster assignment for each gene in adipose, blood, and muscle, with colors corresponding to the cluster profiles in (Figure 3C,3D, and 3F). (B) Features assigned to blood cluster 4 but not muscle clusters 12. (C) Features assigned to blood cluster 5 but not muscle cluster 12. (D) Features assigned to muscle cluster 10.
**Figure S4. Top features for highlighted latent variables in PLIER analysis, related to** Figure 4 A.-H. Cross-tissue PLIER results for RNAseq data.
I.-O. Cross-tissue PLIER results for metabolomics data. Heatmaps indicate within-tissue z-scores of a given feature for each participant at every tissue-time point. The top 10 features associated with each LV are shown.
**Figure S5. Differential feature overlap per modality, tissue, and ome with focus on oppositely regulated features in EE and RE, related to** Figure 5 A. UpSet plot depicting the overlap of significant molecular features across the tissue-modality combinations (regardless of timepoints). Vertical bars indicate the number of features that are unique or shared, while colors represent the omic contribution. Numbers on top of the bars indicate the total number of features in that set. Black dots in the lower matrix indicate the tissue-modality membership for each intersection set.
B. Trajectories of HSPH1. Pre = pre-exercise, P15–45M = 15, 30, 45 minutes post exercise (depending on tissue), P3.5–4H = 3.5 or 4 hours post exercise (depending on tissue, P24H = 24 hours post exercise. Data is shown as group-time point mean ± 95% confidence interval with a black circle indicating significance relative to control at adj. p-value<0.05. HSPH1 = Heat Shock Protein Family H (Hsp110) Member 1. Plot indicates Ensembl ID.
C. Oppositely regulated features in the two exercise modalities (relative to control, adj. p-value<0.05), that were also significant in a direct comparison between EE and RE (adj. p-value<0.05) in SKM. Point color indicates assay and symbol indicates time point.
D. Trajectories of selected features in SKM included in figures 5E and S5C. Pre = pre-exercise, P15–45M = 15, 30, 45 minutes post exercise (depending on tissue), P3.5–4H = 3.5 or 4 hours post exercise (depending on tissue, P24H = 24 hours post exercise. Data is shown as group-time point mean ± 95% confidence interval with a black circle indicating significance relative to control at adj. p-value<0.05. CBFA2T3 = CBFA2/RUNX1 translocation partner 3, MYOG = myogenin, ZBTB4 = Zinc Finger And BTB Domain Containing 4 NOL3 = Nucleolar Protein 3, MYOT = myotilin, lncRNA = long non coding RNA. Plots indicate Ensembl for RNA or Uniprot IDs for proteins where appropriate.
**Figure S6. Differential transcription factor expression, related to** Figure 6 Bubble heatmap of TFs with significant responses satisfying a threshold of adj p-val < 1e-05 at the RNAseq level to EE or RE in at least one time point/modality. Color reflects L2FC of response for each time point/modality comparison and size of dot reflects adjusted p-value. The plot is divided in half to more easily view the large number of TFs with significant responses.
**Figure S7. TFEB pathway regulation in response to EE and RE, related to** Figure 7 A. Heatmap of RNA-seq log2 fold-change of the TFEB-bound targets (based on available ChIP data) in EE and RE.
B. Temporal expression of TFEB transcript in response to exercise across the three tissues. Data are shown as group-time point mean ± 95% confidence interval with a black circle indicating significance relative to control at FDR<0.05.
C. Heatmap of RNA-seq expression of PP2A subunits quantified in both blood and SKM.
D. Temporal expression of HSP90AA1 transcript in response to exercise across the three tissues. Data are shown as group-time point mean ± 95% confidence interval with a black circle indicating significance relative to control at FDR<0.05.
E. Heatmap of log2 fold-change of metabolites upstream of *TFEB*.
**Figure S8. Extended secreted factor analysis and cross-tissue CCN1 correlation, related to** Figure 8 A. UpSet plots showing global and modality-specific features differentially regulated in tissues (adjusted p < 0.1) that are also differentially regulated in plasma proteomes (adjusted p < 0.1). For AT and SKM, gene symbols from transcriptomic, proteomic, and phosphoproteomic layers were concatenated; for blood, transcriptomic data were used. Post-exercise time points were collapsed across modalities. Edges indicate the number of features contributing to each set, and intersecting lines represent shared differential abundance across multiple tissues or layers.
B. Heatmap showing differentially regulated features across muscle, adipose, and blood transcriptomes, proteomes, and phosphoproteomes (adjusted p < 0.1 at post 15–45 min, 3.5–4 hr, and 24 hr post exercise) that also exhibit significant upregulation in plasma proteomes (adjusted p < 0.1 at any time point, including during and post-exercise) that were not included in the Figure 8A. Gene symbols from plasma proteomes were used to map to corresponding features in tissue transcriptomes and phosphoproteomes. “Blood up” and “Blood down” indicate significant upregulation and downregulation in blood proteomes at one or more time points, respectively; “Tissue up” and “Tissue down” indicate significant upregulation or downregulation in tissue omics at one or more time points, respectively. Horizontal green bars denote the extracellular score (range 0–5) from COMPARTMENTS (see Methods); “Both” in the Exercise column indicates features differentially regulated in both EE and RE.
C. Temporal trajectory of PXN phosphorylation in AT and SKM.. EE = Endurance Exercise, RE = Resistance Exercise, CON = control group, Pre = pre-exercise, D20M = during 20 minutes, D40M = during 40 minutes, P10M = 10 minutes post exercise, P15–45M = 15, 30, or 45 minutes post exercise (depending on tissue), P3.5–4H = 3.5 or 4 hours post exercise (depending on tissue), P24H = 24 hours post exercise. Data is shown as group-time point mean ± 95% confidence interval for the group mean with a black circle indicating model significance relative to control at adj. p-val<0.1. Plots indicate Uniprot accession number for PXN along with the specific phosphosite.
D. Correlation plots of *CCN1* transcript expression in each group (CON, EE, RE) between adipose and muscle at each time point (Pre-exercise, post 30/45 min, post 3.5/4 hr, and post 24 hr). Pearson correlation and p-values are assessed for each comparison.

Document S1. Figures S1–S8.

## Figures and Tables

**Table 1.** Participant Characteristics

Characteristic	Overall N = 175	EE N = 65	RE N = 73	CON N = 37
**Demographics**				
Sex				
Female	126 (72%)	46 (71%)	49 (67%)	31 (84%)
Male	49 (28%)	19 (29%)	24 (33%)	6 (16%)
Age (yrs)	41 (15)	42 (14)	40 (15)	42 (15)
Height (cm)	168 (9)	168 (9)	168 (10)	167 (8)
Weight (kg)	76 (14)	76 (13)	77 (14)	73 (13)
BMI (kg/m^2^)	26.9 (4.0)	27.0 (4.0)	27.2 (4.0)	26.2 (3.9)
Waist Circumference (cm)	92 (12)	93 (11)	93 (12)	89 (13)
Systolic BP (mmHg)	116 (12)	117 (11)	116 (12)	114 (11)
Diastolic BP (mmHg)	72 (8)	73 (9)	72 (8)	72 (8)
Hemoglobin A1c (%)	5.30 (0.30)	5.29 (0.32)	5.31 (0.32)	5.29 (0.21)
eGFR (mL/min/1.73 m^2^)	101 (20)	103 (18)	101 (22)	99 (19)
**Cardiopulmonary Exercise Test (CPET)**				
VO_2_peak (L)	1.86 (0.65)	1.90 (0.68)	1.89 (0.65)	1.73 (0.56)
VO_2_peak (mL/min/kg)	24 (7)	25 (7)	25 (7)	24 (6)
Workload (Watts)	152 (50)	155 (53)	153 (52)	142 (40)
O_2_ Pulse (ml/beat)	10.6 (3.3)	10.9 (3.5)	10.8 (3.4)	10.0 (2.8)
RER	1.16 (0.08)	1.17 (0.07)	1.15 (0.08)	1.16 (0.08)
**Strength Tests**				
Isometric Knee Extension (Nm)	148 (57)	145 (54)	156 (60)	137 (53)
Hand Grip (kg)	30 (10)	30 (10)	31 (12)	28 (7)

Values are mean (SD) for continuous variables and n (%) for categorical variables. Abbreviations: EE = endurance exercise; RE = resistance exercise; CON = control; BMI = body mass index; BP = blood pressure; eGFR = estimated glomerular filtration rate; CPET = cardiopulmonary exercise testing, and RER = Respiratory Exchange Ratio.
