# Supplementary material for: Multi-Omic, Multi-Tissue Responses to Acute Exercise in Sedentary Adults: Findings from the Molecular Transducers of Physical Activity Consortium
Source: bioRxiv. 2026 Mar 2:2026.02.27.702183. Preprint. [Version 1] doi: 10.64898/2026.02.27.702183 (PMC13184684; doi:10.64898/2026.02.27.702183)
Supplement: 1 — Figure S1. Participant genotypes, sample-ome overlap distribution and tissue-ome covariate structure, related to Figure 1 A. First two principal components of genetic ancestry. Participants from MoTrPAC are plotted using triangles, while participants from 1000 Genomes are plotted using points. Categorical ancestry according to 1000 Genomes is indicated by color. MoTrPAC participants are not categorized by ancestry. B. Matrix of omic data availability. Each row of the heatmap represents a participant, with each participant represented once in each of the three tissues. If data is available for a particular participant for a given tissue-ome-time point combination, this is indicated by a colored cell in the matrix. A grey cell indicates no omic data available for the sample collected. A white cell indicates that no sample was collected in that participant for that tissue-time point. C. Canonical correlation analysis of the first five principal components of ome-tissue combinations to key MoTrPAC experimental covariates. Colors in each block represent covariance between each principal component and each experimental parameter. Metabolite platforms were combined into one matrix and subset to participant-timepoint combinations present in all metabolomic platforms. Principal component labels indicate percentage of total omic variance explained by each principal component. D. Percentages of variance explained by experimental design parameters for each feature in every available ome-tissue combination. After fitting a linear mixed model for each feature with complete data, the percentage of variance explained per covariate per feature is represented on the y axis. Residual variance is not visualized in this plot. A grey cell (adipose ATAC, blood phosphoproteomics) indicates no omic data where a sample was collected. Figure S2. Distribution and overlap of differential multi-omic features across tissues, related to Figure 2 A. Barplot summarizing the classification of molecul [file NIHPP2026.02.27.702183V1-supplement-1.pdf]

bioRxiv preprint doi: https://doi.org/10.64898/2026.02.27.702183; this version posted March 3, 2026. The copyright holder for this preprint (which was not certified by peer review) is the author/funder, who has granted bioRxiv a license to display the preprint in perpetuity. It is made available under aCC-BY 4.0 International license.

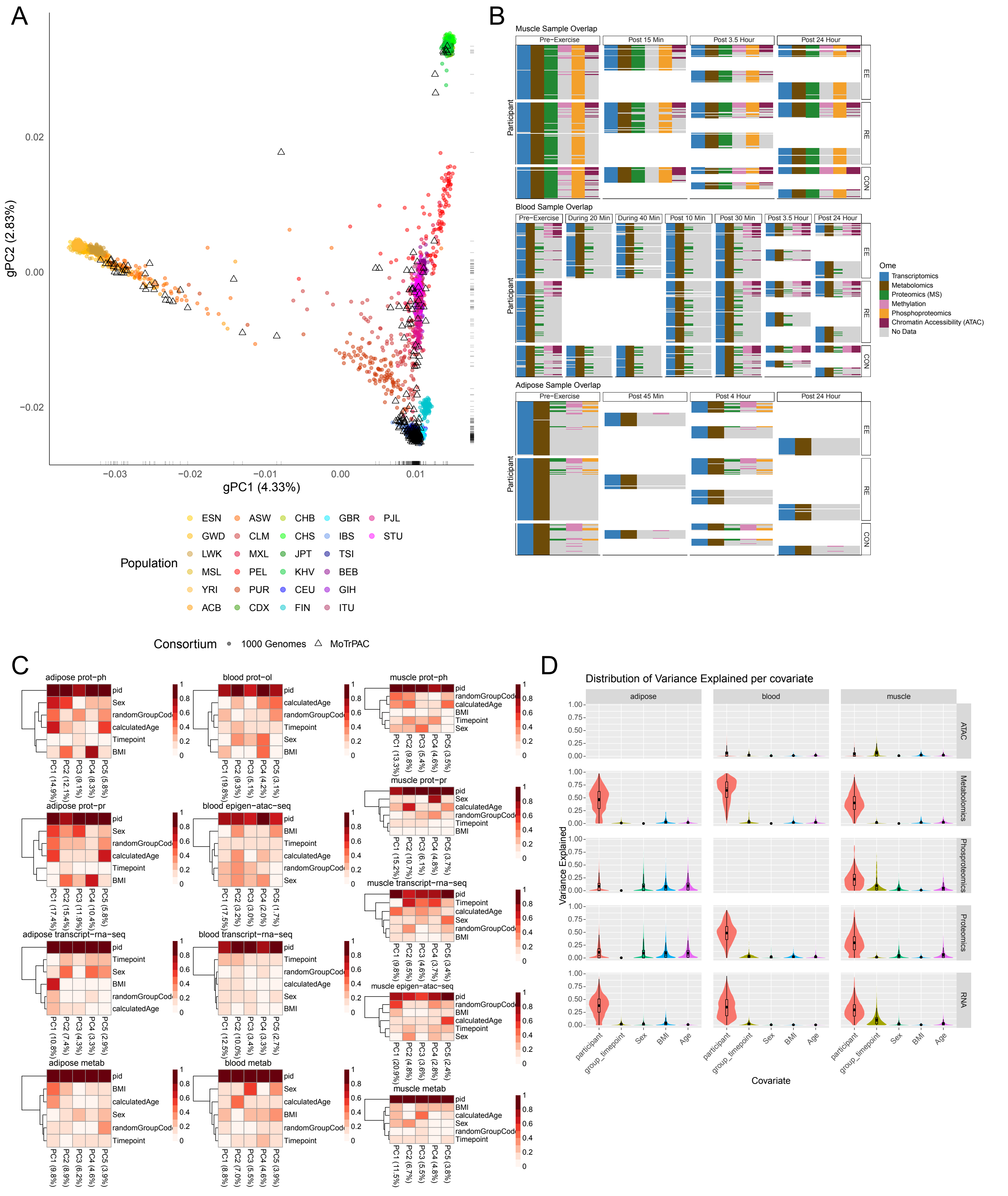

Figure S1. Participant genotypes, sample-ome overlap distribution and tissue-ome covariate structure, related to Figure 1

A. First two principal components of genetic ancestry. Participants from MoTrPAC are plotted using triangles, while participants from 1000 Genomes are plotted using points. Categorical ancestry

bioRxiv preprint doi: https://doi.org/10.64898/2026.02.27.702183; this version posted March 3, 2026. The copyright holder for this preprint (which was not certified by peer review) is the author/funder, who has granted bioRxiv a license to display the preprint in perpetuity. It is made available under aCC-BY 4.0 International license.

according to 1000 Genomes is indicated by color. MoTrPAC participants are not categorized by ancestry.

B. Matrix of omic data availability. Each row of the heatmap represents a participant, with each participant represented once in each of the three tissues. If data is available for a particular participant for a given tissue-ome-time point combination, this is indicated by a colored cell in the matrix. A grey cell indicates no omic data available for the sample collected. A white cell indicates that no sample was collected in that participant for that tissue-time point.

C. Canonical correlation analysis of the first five principal components of ome-tissue combinations to key MoTrPAC experimental covariates. Colors in each block represent covariance between each principal component and each experimental parameter. Metabolite platforms were combined into one matrix and subset to participant-timepoint combinations present in all metabolomic platforms. Principal component labels indicate percentage of total omic variance explained by each principal component.

D. Percentages of variance explained by experimental design parameters for each feature in every available ome-tissue combination. After fitting a linear mixed model for each feature with complete data, the percentage of variance explained per covariate per feature is represented on the y axis. Residual variance is not visualized in this plot. A grey cell (adipose ATAC, blood phosphoproteomics) indicates no omic data where a sample was collected.

bioRxiv preprint doi: https://doi.org/10.64898/2026.02.27.702183; this version posted March 3, 2026. The copyright holder for this preprint (which was not certified by peer review) is the author/funder, who has granted bioRxiv a license to display the preprint in perpetuity. It is made available under aCC-BY 4.0 International license.

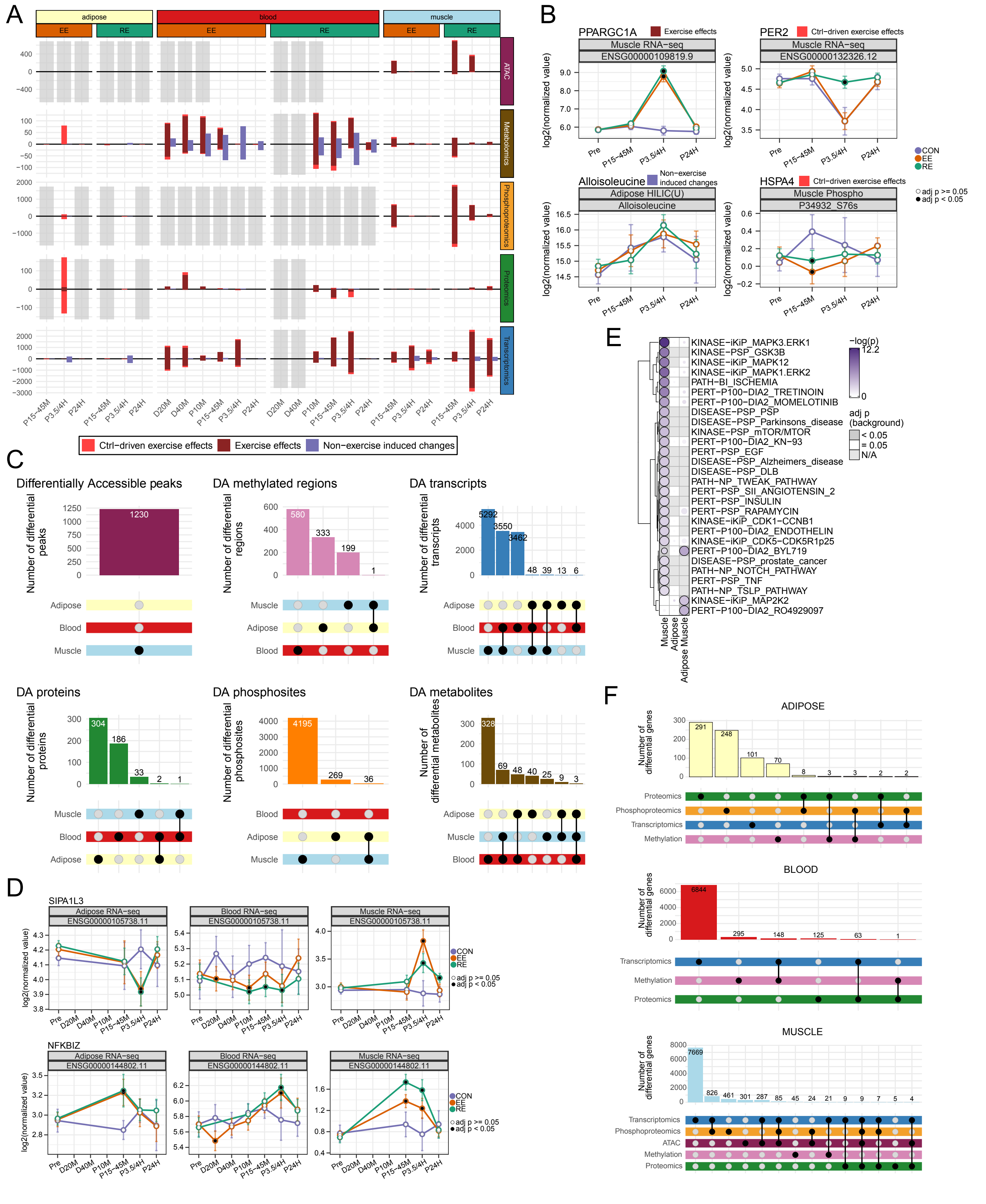

Figure S2. Distribution and overlap of differential multi-omic features across tissues, related to Figure 2

A. Barplot summarizing the classification of molecular features by type of differential response across tissues and omes. Features were grouped into three categories based on significance patterns in

bioRxiv preprint doi: https://doi.org/10.64898/2026.02.27.702183; this version posted March 3, 2026. The copyright holder for this preprint (which was not certified by peer review) is the author/funder, who has granted bioRxiv a license to display the preprint in perpetuity. It is made available under aCC-BY 4.0 International license.

exercise versus control comparisons: (1) Control-driven exercise effects; (2) Exercise effects; and (3) Non-exercise induced changes. This categorization is intended to distinguish biologically meaningful exercise responses from potential false positives from otherwise uncontrolled experimental designs. Each feature upregulated relative to control is plotted above the axis, and each downregulated relative to control is plotted below the axis.

B. Representative examples of molecular features classified into categories based on their differential response patterns across timepoints and conditions. Alloisoleucine (Adipose metabolomics) illustrates non-exercise induced changes. *PPARGC1A* (Muscle RNA-seq) represents a robust exercise effect. HSPA4 (Muscle Phosphoproteomics) and *PER2* (Muscle RNA-seq) exemplifies a control-driven effect. Pre = pre-exercise, P15-45M = 15, 30, 45 minutes post exercise (depending on tissue), P3.5-4H = 3.5 or 4 hours post exercise (depending on tissue, P24H = 24 hours post exercise. Data is shown as group-time point mean ± 95% confidence interval with a black circle indicating significance relative to control at adj. p-val<0.05. Plots indicate Ensembl for RNA or Uniprot IDs for proteins where appropriate.

C. UpSet plots by ome across tissues: ATAC-seq, MethylCap-seq, transcriptomics, proteomics, phosphoproteomics, and metabolomics. Each plot displays the overlap of significant features (adjusted p-value < 0.05) across three tissues (AT, SKM, and blood) regardless of exercise modality or timepoint. Vertical bars represent the number of features unique to or shared among the tissues, as shown in the matrix below each plot. Numbers on top of the bars indicate the total number of features in that set.

D. Over-representation analysis of DA phosphosites unique or shared between the AT and SKM using PTMsigDB.

E. Temporal trajectories of select DA features shared between all three tissues. Pre = pre-exercise, D20M = during 20 minutes, D40M = during 40 minutes, P10M = 10 minutes post exercise, P15-45M = 15, 30, 45 minutes post exercise (depending on tissue), P3.5-4H = 3.5 or 4 hours post exercise (depending on tissue, P24H = 24 hours post exercise. Points represent group means ± standard error; black points circles denote comparisons to control with adjusted p-values < 0.05. Plots indicate Ensembl for RNA.

F. Panel of three UpSet plots, one for each tissue (AT, SKM, and blood), illustrating the intersection of all significant features (adjusted p-value < 0.05) across epigenome (ATAC-seq, MethyCap-seq), transcriptome, proteome, and phosphoproteome (metabolomics data was not included). Features were mapped to gene symbols to enable direct comparison across omics layers. For each tissue, vertical bars represent the number of features unique to, or shared among, the indicated omes as shown by the matrix below each plot. Features that were DA at any time point and in either modality were considered for matching across omic layers.

bioRxiv preprint doi: https://doi.org/10.64898/2026.02.27.702183; this version posted March 3, 2026. The copyright holder for this preprint (which was not certified by peer review) is the author/funder, who has granted bioRxiv a license to display the preprint in perpetuity. It is made available under aCC-BY 4.0 International license.

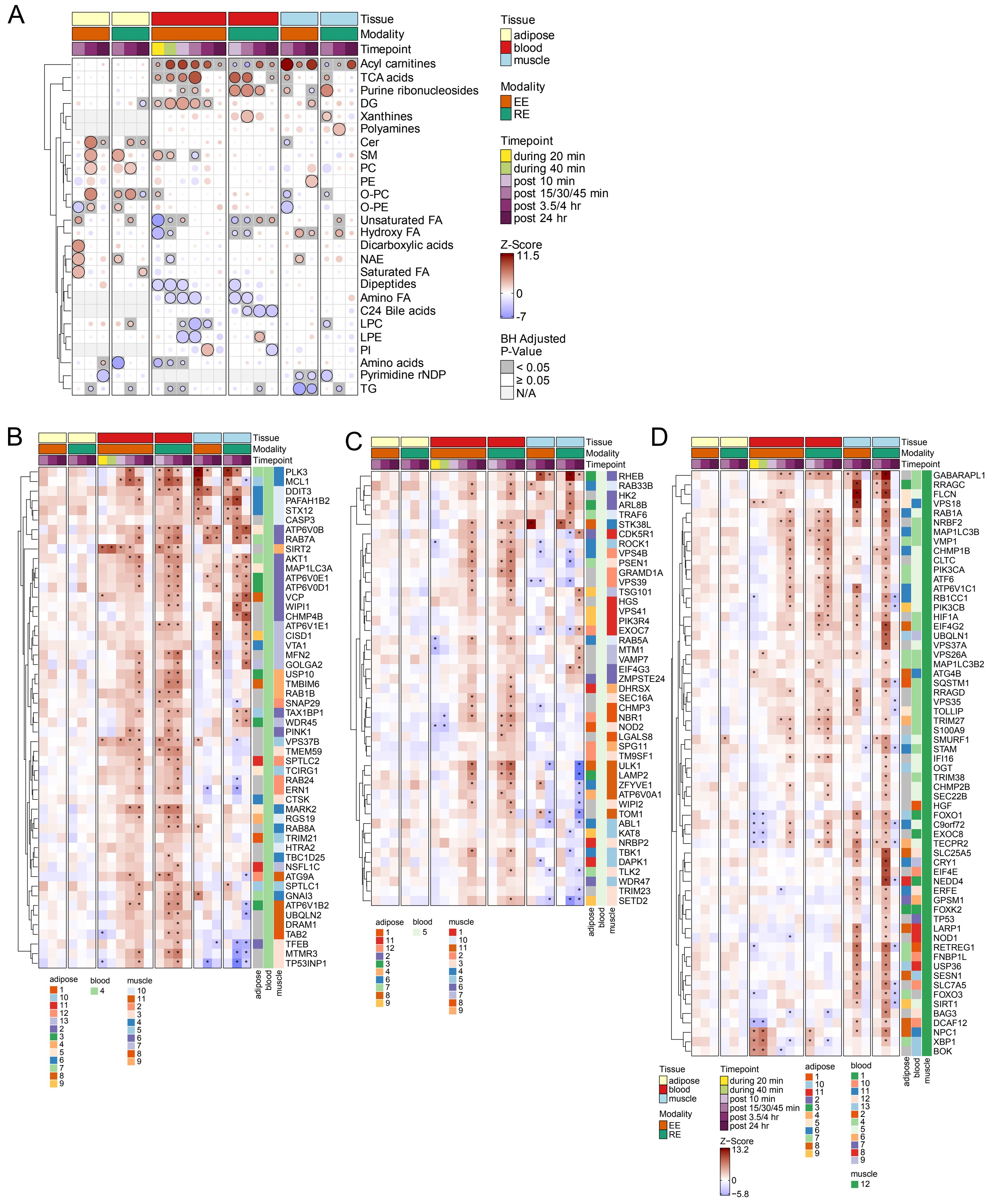

Figure S3. Multi-tissue metabolomic enrichments and gene heatmaps of significant C means trajectory pathways, related to Figure 3

A. Pathway Enrichment for each tissue-timepoint in metabolomic RefMet classes via Pre-ranked Correlation-Adjusted MEan-RAnk (CAMERA-PR).

bioRxiv preprint doi: https://doi.org/10.64898/2026.02.27.702183; this version posted March 3, 2026. The copyright holder for this preprint (which was not certified by peer review) is the author/funder, who has granted bioRxiv a license to display the preprint in perpetuity. It is made available under aCC-BY 4.0 International license.

B.-D. Multi-tissue heatmaps of Z-scores for transcriptomic features belonging to the GOBP: PROCESS UTILIZING AUTOPHAGIC MECHANISM. Columns represent time points grouped by tissue and exercise modality. The right-hand annotation indicates the c-means cluster assignment for each gene in adipose, blood, and muscle, with colors corresponding to the cluster profiles in (Figure 3C,3D, and 3F). (B) Features assigned to blood cluster 4 but not muscle clusters 12. (C) Features assigned to blood cluster 5 but not muscle cluster 12. (D) Features assigned to muscle cluster 10.

bioRxiv preprint doi: https://doi.org/10.64898/2026.02.27.702183; this version posted March 3, 2026. The copyright holder for this preprint (which was not certified by peer review) is the author/funder, who has granted bioRxiv a license to display the preprint in perpetuity. It is made available under aCC-BY 4.0 International license.

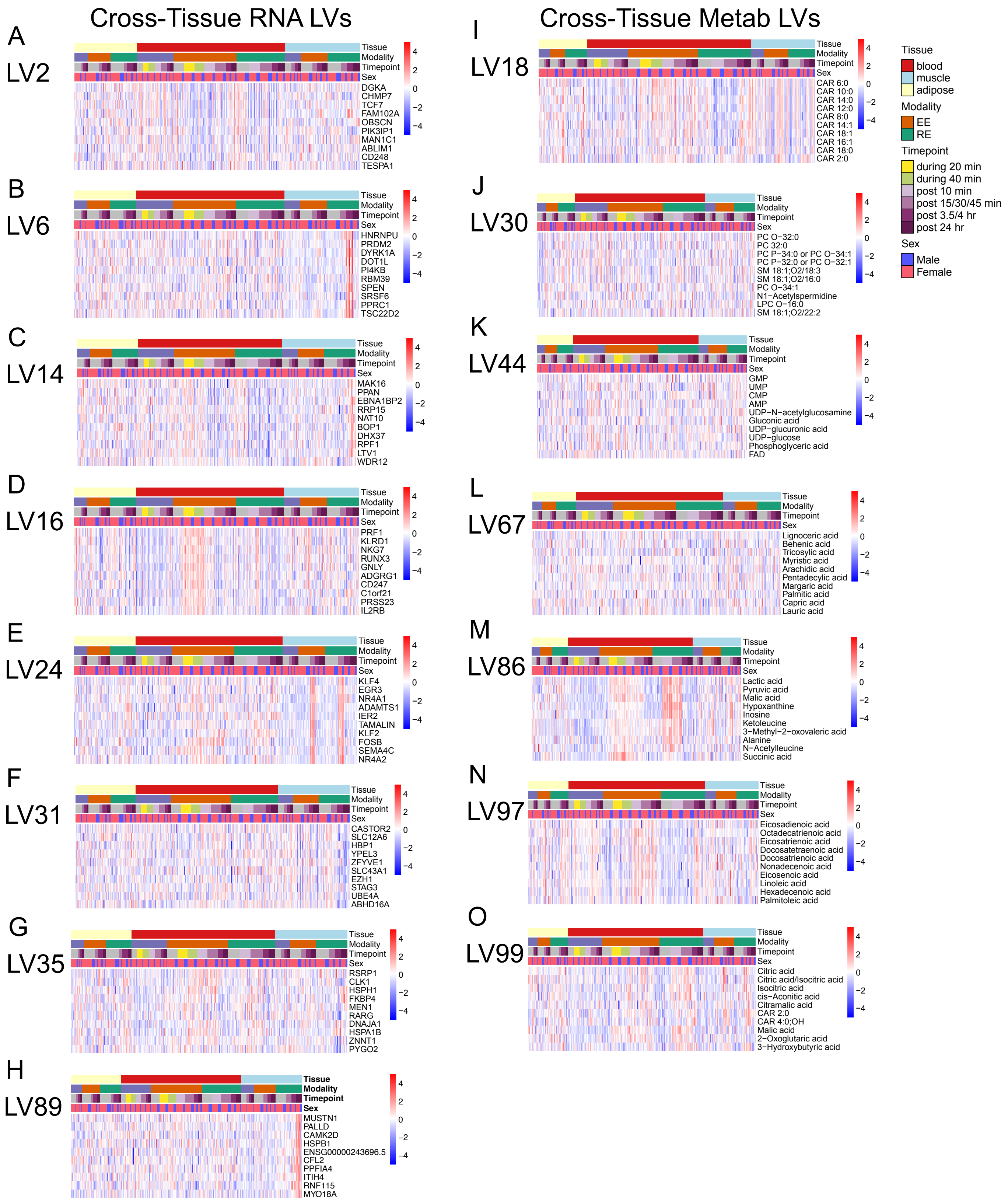

Figure S4. Top features for highlighted latent variables in PLIER analysis, related to Figure 4

A.-H. Cross-tissue PLIER results for RNAseq data.

I.-O. Cross-tissue PLIER results for metabolomics data. Heatmaps indicate within-tissue z-scores of a given feature for each participant at every tissue-time point. The top 10 features associated with each LV are shown.

bioRxiv preprint doi: https://doi.org/10.64898/2026.02.27.702183; this version posted March 3, 2026. The copyright holder for this preprint (which was not certified by peer review) is the author/funder, who has granted bioRxiv a license to display the preprint in perpetuity. It is made available under aCC-BY 4.0 International license.

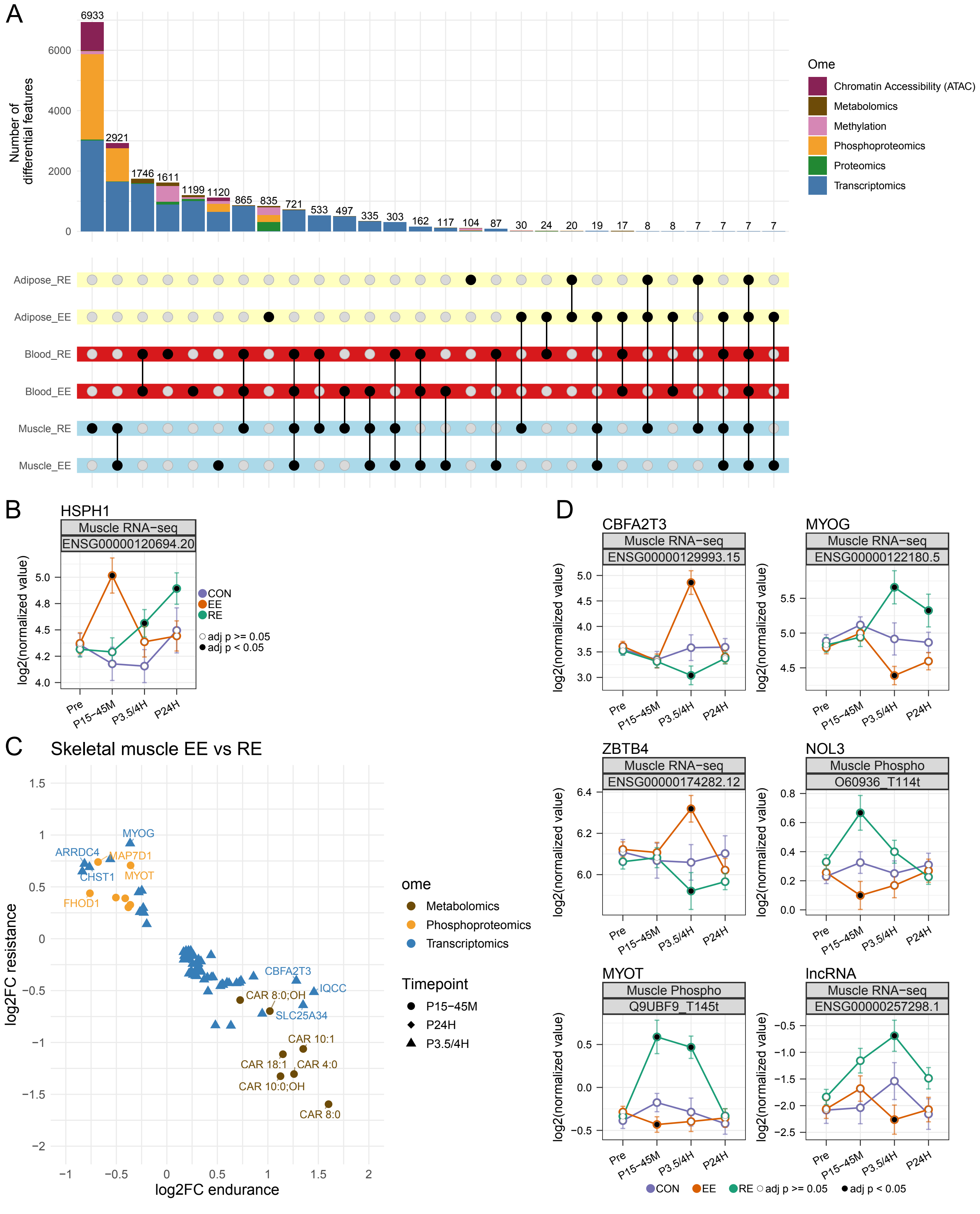

Figure S5. Differential feature overlap per modality, tissue, and ome with focus on oppositely regulated features in EE and RE, related to Figure 5

A. UpSet plot depicting the overlap of significant molecular features across the tissue-modality combinations (regardless of timepoints). Vertical bars indicate the number of features that are unique or

bioRxiv preprint doi: https://doi.org/10.64898/2026.02.27.702183; this version posted March 3, 2026. The copyright holder for this preprint (which was not certified by peer review) is the author/funder, who has granted bioRxiv a license to display the preprint in perpetuity. It is made available under aCC-BY 4.0 International license.

shared, while colors represent the omic contribution. Numbers on top of the bars indicate the total number of features in that set. Black dots in the lower matrix indicate the tissue-modality membership for each intersection set.

B. Trajectories of HSPH1. Pre = pre-exercise, P15-45M = 15, 30, 45 minutes post exercise (depending on tissue), P3.5-4H = 3.5 or 4 hours post exercise (depending on tissue, P24H = 24 hours post exercise. Data is shown as group-time point mean ± 95% confidence interval with a black circle indicating significance relative to control at adj. p-value<0.05. HSPH1 = Heat Shock Protein Family H (Hsp110) Member 1. Plot indicates Ensembl ID.
C. Oppositely regulated features in the two exercise modalities (relative to control, adj. p-value<0.05), that were also significant in a direct comparison between EE and RE (adj. p-value<0.05) in SKM. Point color indicates assay and symbol indicates time point.
D. Trajectories of selected features in SKM included in figures 5E and S5C. Pre = pre-exercise, P15-45M = 15, 30, 45 minutes post exercise (depending on tissue), P3.5-4H = 3.5 or 4 hours post exercise (depending on tissue, P24H = 24 hours post exercise. Data is shown as group-time point mean ± 95% confidence interval with a black circle indicating significance relative to control at adj. p-value<0.05. CBFA2T3 = CBFA2/RUNX1 translocation partner 3, MYOG = myogenin, ZBTB4 = Zinc Finger And BTB Domain Containing 4 NOL3 = Nucleolar Protein 3, MYOT = myotilin, lncRNA = long non coding RNA. Plots indicate Ensembl for RNA or Uniprot IDs for proteins where appropriate.

bioRxiv preprint doi: https://doi.org/10.64898/2026.02.27.702183; this version posted March 3, 2026. The copyright holder for this preprint (which was not certified by peer review) is the author/funder, who has granted bioRxiv a license to display the preprint in perpetuity. It is made available under aCC-BY 4.0 International license.

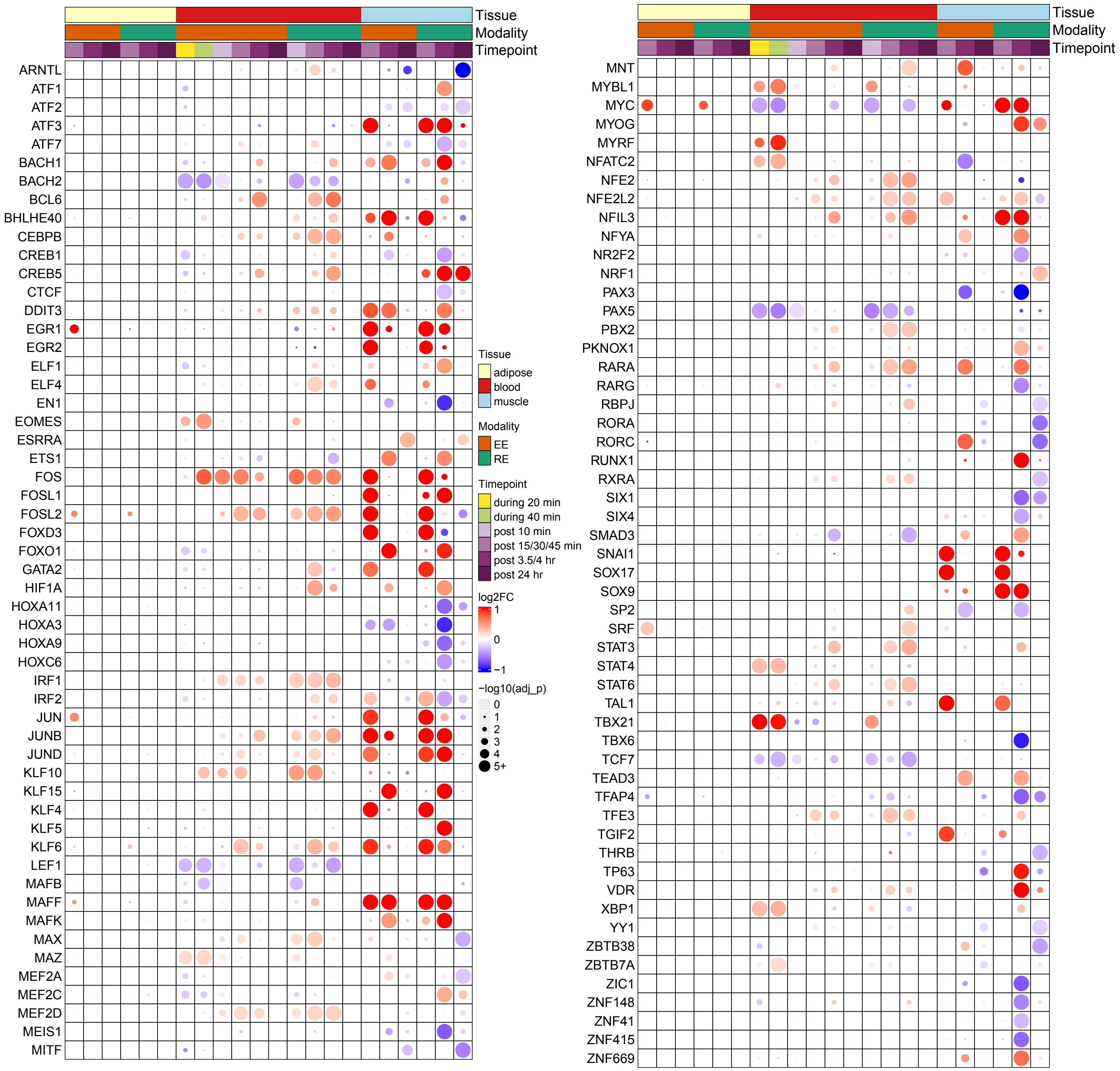

Figure S6. Differential transcription factor expression, related to Figure 6

Bubble heatmap of TFs with significant responses satisfying a threshold of adj p-val < 1e-05 at the RNAseq level to EE or RE in at least one time point/modality. Color reflects L2FC of response for each time point/modality comparison and size of dot reflects adjusted p-value. The plot is divided in half to more easily view the large number of TFs with significant responses.

bioRxiv preprint doi: https://doi.org/10.64898/2026.02.27.702183; this version posted March 3, 2026. The copyright holder for this preprint (which was not certified by peer review) is the author/funder, who has granted bioRxiv a license to display the preprint in perpetuity. It is made available under aCC-BY 4.0 International license.

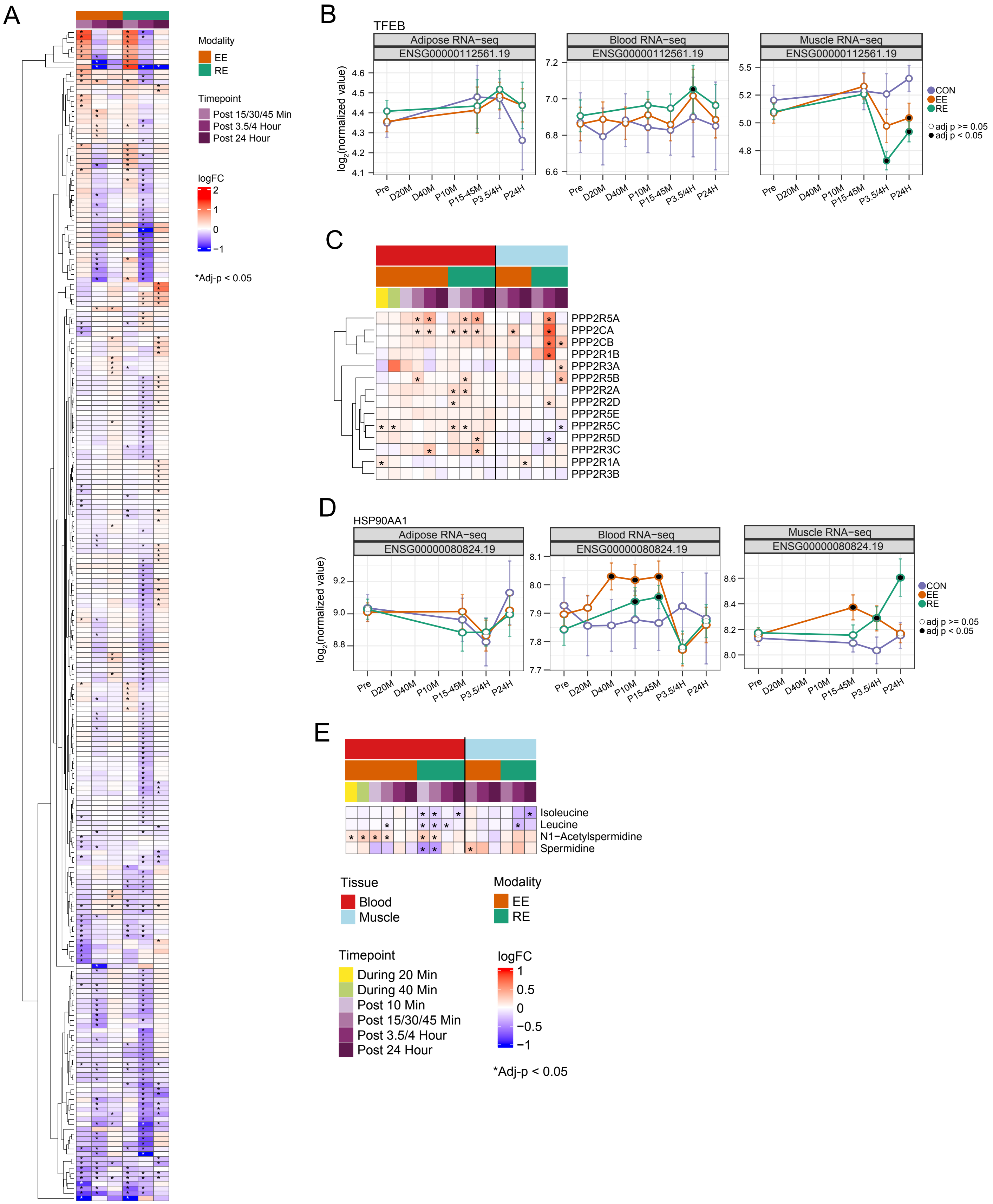

Figure S7. TFEB pathway regulation in response to EE and RE, related to Figure 7

A. Heatmap of RNA-seq log2 fold-change of the TFEB-bound targets (based on available ChIP data) in EE and RE.

bioRxiv preprint doi: https://doi.org/10.64898/2026.02.27.702183; this version posted March 3, 2026. The copyright holder for this preprint (which was not certified by peer review) is the author/funder, who has granted bioRxiv a license to display the preprint in perpetuity. It is made available under aCC-BY 4.0 International license.

B. Temporal expression of TFEB transcript in response to exercise across the three tissues. Data are shown as group-time point mean ± 95% confidence interval with a black circle indicating significance relative to control at FDR<0.05.
C. Heatmap of RNA-seq expression of PP2A subunits quantified in both blood and SKM.
D. Temporal expression of HSP90AA1 transcript in response to exercise across the three tissues. Data are shown as group-time point mean ± 95% confidence interval with a black circle indicating significance relative to control at FDR<0.05.
E. Heatmap of log2 fold-change of metabolites upstream of *TFEB*.

bioRxiv preprint doi: https://doi.org/10.64898/2026.02.27.702183; this version posted March 3, 2026. The copyright holder for this preprint (which was not certified by peer review) is the author/funder, who has granted bioRxiv a license to display the preprint in perpetuity. It is made available under aCC-BY 4.0 International license.

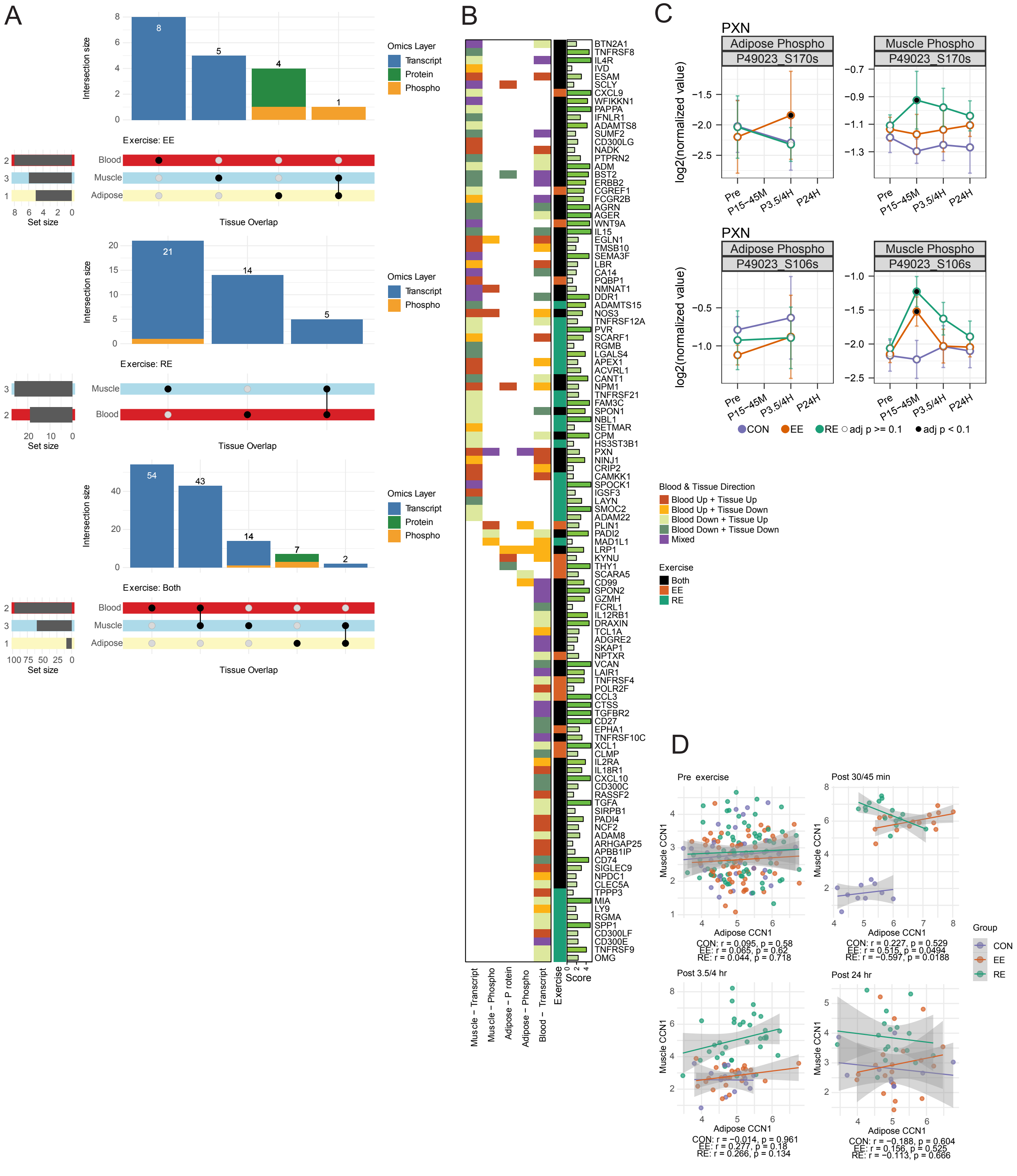

Figure S8. Extended secreted factor analysis and cross-tissue CCN1 correlation, related to Figure 8

A. UpSet plots showing global and modality-specific features differentially regulated in tissues (adjusted p < 0.1) that are also differentially regulated in plasma proteomes (adjusted p < 0.1). For AT and SKM, gene symbols from transcriptomic, proteomic, and phosphoproteomic layers were concatenated; for blood, transcriptomic data were used. Post-exercise time points were collapsed across modalities.

bioRxiv preprint doi: https://doi.org/10.64898/2026.02.27.702183; this version posted March 3, 2026. The copyright holder for this preprint (which was not certified by peer review) is the author/funder, who has granted bioRxiv a license to display the preprint in perpetuity. It is made available under aCC-BY 4.0 International license.

Edges indicate the number of features contributing to each set, and intersecting lines represent shared differential abundance across multiple tissues or layers.

B. Heatmap showing differentially regulated features across muscle, adipose, and blood transcriptomes, proteomes, and phosphoproteomes (adjusted $p < 0.1$ at post 15-45 min, 3.5-4 hr, and 24 hr post exercise) that also exhibit significant upregulation in plasma proteomes (adjusted $p < 0.1$ at any time point, including during and post-exercise) that were not included in the Figure 8A. Gene symbols from plasma proteomes were used to map to corresponding features in tissue transcriptomes and phosphoproteomes. “Blood up” and "Blood down" indicate significant upregulation and downregulation in blood proteomes at one or more time points, respectively; “Tissue up” and "Tissue down" indicate significant upregulation or downregulation in tissue omics at one or more time points, respectively. Horizontal green bars denote the extracellular score (range 0–5) from COMPARTMENTS (see Methods); “Both" in the Exercise column indicates features differentially regulated in both EE and RE.

C. Temporal trajectory of PXN phosphorylation in AT and SKM. . EE = Endurance Exercise, RE = Resistance Exercise, CON = control group, Pre = pre-exercise, D20M = during 20 minutes, D40M = during 40 minutes, P10M = 10 minutes post exercise, P15-45M = 15, 30, or 45 minutes post exercise (depending on tissue), P3.5-4H = 3.5 or 4 hours post exercise (depending on tissue), P24H = 24 hours post exercise. Data is shown as group-time point mean ± 95% confidence interval for the group mean with a black circle indicating model significance relative to control at adj. p-val<0.1. Plots indicate Uniprot accession number for PXN along with the specific phosphosite.

D. Correlation plots of *CCN1* transcript expression in each group (CON, EE, RE) between adipose and muscle at each time point (Pre-exercise, post 30/45 min, post 3.5/4 hr, and post 24 hr). Pearson correlation and p-values are assessed for each comparison.
